# Quantifying Transcriptome Turnover on Phylogenies by Modeling Gene Expression as a Binary Trait

**DOI:** 10.1093/molbev/msaf106

**Published:** 2025-05-27

**Authors:** Ammon Thompson, Michael R May, Ben R Hopkins, Nerisa Riedl, Olga Barmina, Benjamin J Liebeskind, Li Zhao, David Begun, Artyom Kopp

**Affiliations:** Department of Evolution and Ecology, University of California, Davis, CA, USA; Department of Evolution and Ecology, University of California, Davis, CA, USA; Department of Evolution and Ecology, University of California, Davis, CA, USA; Department of Evolution and Ecology, University of California, Davis, CA, USA; Department of Evolution and Ecology, University of California, Davis, CA, USA; Center for Systems and Synthetic Biology, Department of Molecular Biosciences, University of Texas at Austin, Austin, TX, USA; Department of Evolution and Ecology, University of California, Davis, CA, USA; Laboratory of Evolutionary Genetics and Genomics, The Rockefeller University, New York, NY, USA; Department of Evolution and Ecology, University of California, Davis, CA, USA; Department of Evolution and Ecology, University of California, Davis, CA, USA

**Keywords:** evolution, gene expression, transcriptome, phylogenetics, Drosophila, testis, accessory glands

## Abstract

Changes in gene expression are a key driver of phenotypic evolution, leading to a persistent interest in the evolution of transcriptomes. Traditionally, gene expression is modeled as a continuous trait, leaving qualitative transitions largely unexplored. In this paper, we detail the development of new Bayesian inference techniques to study the evolutionary turnover of organ-specific transcriptomes, which we define as instances where orthologous genes gain or lose expression in a particular organ. To test these techniques, we analyze the transcriptomes of 2 male reproductive organs, testes and accessory glands, across 11 species of the Drosophila melanogaster species group. We first discretize gene expression states by estimating the probability that each gene is expressed in each organ and species. We then define a phylogenetic model of correlated transcriptome evolution in 2 or more organs and fit it to the expression state data. Inferences under this model imply that many genes have gained and lost expression in each organ, and that the 2 organs experienced accelerated transcriptome turnover on different branches of the Drosophila phylogeny.

## Introduction

Phenotypic evolution, broadly speaking, can result from 3 general mechanisms: gains and losses of genes; mutations in the coding sequences of genes, leading to changes in protein functions; and regulatory mutations, which lead to changes in gene expression. While the exact balance between these modes of genetic change in driving the origin of new phenotypes is a debated topic, it is clear that regulatory evolution plays a prominent role (King and Wilson 1975; Hoekstra and Coyne 2007; Wray 2007; Carroll 2008; Stern and Orgogozo 2008 ; Courtier-Orgogozo et al. 2020). Across a diverse range of taxa and traits, changes in the expression of orthologous genes are pivotal in creating major phenotypic differences within and between species (e.g. Rebeiz et al. 2009; Fraser et al. 2010; Loehlin et al. 2019; Kowalczyk et al. 2022; Marand et al. 2023).

RNA sequencing (RNA-seq) technology has enabled quantification of gene expression levels for the entire transcriptome across diverse biological conditions and developmental stages (Mortazavi et al. 2008; Wang et al. 2009; Marguerat and Bähler 2010; Hrdlickova et al. 2017). As a result, RNA-seq has become essential for studying regulatory evolution, providing comprehensive and quantitative data for inferring large- and small-scale changes in transcriptomes associated with adaptive evolution (Harrison et al. 2012; Wittkopp and Kalay 2012; Todd et al. 2016). Transcriptome-level studies of gene expression evolution have largely focused on quantitative differences between species, modeling a gene’s expression as a continuous trait. Indeed, most evolutionary changes in gene expression are of a quantitative nature (Rifkin et al. 2003; Brawand et al. 2011; Harrison et al. 2012; Romero et al. 2012; Coolon et al. 2014; Nourmohammad et al. 2017; Cardoso-Moreira et al. 2019). By contrast, qualitative changes, manifested as discrete gain and loss of gene expression in a particular tissue over evolutionary time (“transcriptome turnover”), have received comparatively little attention (Mika et al. 2021, 2022). Consequently, questions about the rates of transcriptome turnover, the consistency of these rates over time and among lineages, and the degree of correlation in turnover rates between different organs remain unresolved. This may be an important deficit in our understanding of phenotypic evolution given the many examples where gain or loss of gene expression in an organ has a profound impact on its structure or function (Glassford et al. 2015; Hu et al. 2018; Kellenberger et al. 2023; Molina-Gil et al. 2023).

Studying gene expression at a qualitative level brings with it two significant advantages. The first is biological. Relative to quantitative changes, deactivation of a gene, or activation of an ancestrally inactive gene, in a particular organ might be expected to have a more profound impact on phenotypic evolution (Harlin-Cognato et al. 2006; Wray 2007; Carroll 2008; Tanaka et al. 2011; Glassford et al. 2015; Thompson et al. 2016, 2018; Martinson et al. 2017; Hu et al. 2018). The generality of this idea, which is currently supported by anecdotal examples, needs to be tested systematically at the genome-wide level. The second advantage is technical. If inferred reliably, discrete expression states should be less sensitive to technical factors such as sequencing depth and library size, differences in cell type composition caused by dissection variability, and the well-documented problems of comparing compositional values such as TPM or FPKM between tissues and species (Brawand et al. 2011; Dillies et al. 2013; Li et al. 2014; Musser and Wagner 2015). While the effect of some of these variables on cross-species and cross-tissue quantitative analyses can in principle be minimized through normalization approaches, selecting an appropriate evolutionary model for data under complex transformations and normalization schemes can be challenging to implement and interpret (Vandesompele et al. 2002; de Jonge et al. 2007; Brawand et al. 2011; Dillies et al. 2013; Chen et al. 2014; Quinn et al. 2018; Cardoso-Moreira et al. 2019; Zhou et al. 2019; Dimayacyac et al. 2023; Mantica et al. 2024).

The main obstacle to investigating transcriptome turnover on a genome-wide scale is the difficulty of translating the continuous RNA-seq data (TPM or FPKM) into discrete (ON/OFF) gene expression states. Many genes that play important roles in development can be expressed at low levels in just a few cells, making their expression difficult to detect reliably. Conversely, some genes that are not actively expressed in a particular tissue may nevertheless be detected at non-zero levels in a transcriptome sample due to technical artifacts or non-specific transcription that occurs throughout the genome (Singh and Petrov 2007; Struhl 2007; Schwanhäusser et al. 2011; Jensen et al. 2013; Wagner et al. 2013; Artieri and Fraser 2014; Kin et al. 2015; Lenive et al. 2016; Ham et al. 2020). Historically, gene expression states in transcriptomes have been discretized using hard expression thresholds; for example, genes present above 1–3 TPM may be considered actively expressed, while those below 1 TPM are assigned as inactive (Mortazavi et al. 2008; Hebenstreit et al. 2011; Wagner et al. 2013; Huang et al. 2015; Cridland et al. 2020; Mika et al. 2021, 2022).

In some cases, specific expression cut-offs are well justified on mechanistic biological grounds implied by chromatin marks associated with active or repressed transcription (Singh and Petrov 2007; Ernst et al. 2011; Hart et al. 2013; Wagner et al. 2013). However, fixed cut-offs on relative expression levels are poorly suited to cross-species and cross-tissue comparisons, since it is far from clear that a single expression threshold can accurately distinguish between active expression and transcriptional noise in all genes, species, and tissues, especially when the species are distantly related and/or the tissues differ strongly in transcriptome complexity. A change in expression of a small subset of highly expressed genes will alter the relative expression of the rest of the distribution thus shifting the threshold without any changes in expression among the rest of the genes. In fact, our recent work has shown that the boundary between active expression and noise is different even among tissues that are physiologically similar (e.g. different primate brain regions; Thompson et al. 2020).

To develop a more objective and generally applicable approach to discretizing gene expression states, we recently created and validated a method, *zigzag* (Thompson et al. 2020), for inferring gene expression states from replicated RNA-seq datasets. *zigzag* uses Markov chain Monte Carlo to estimate the posterior probability of active expression (the “ON” state) for each gene in each tissue using a well-defined statistical model and testable prior assumptions, enabling easy validation. *zigzag* achieves this by learning universal landmarks in transcriptome datasets that distinguish between active and inactive genes (Hebenstreit et al. 2011; Hart et al. 2013; Wagner et al. 2013; Huang et al. 2015; Thompson et al. 2020; Costa et al. 2022). This method was shown to be sensitive enough to correctly classify expression states of transcription factors expressed in only a few cells in the drosophila testes while classifying smell and taste receptor genes as largely inactive in the same tissue despite detecting reads mapping to these genes (Thompson et al. 2020). This tool allows researchers to either use the probabilities in downstream analyses or set thresholds based on the probabilities which, unlike thresholds on relative measures, are directly comparable between species and tissues. The ability to classify gene expression states probabilistically using *zigzag* opens the way for investigating the evolutionary turnover of tissue-specific transcriptomes (i.e. the transition of conserved genes between OFF and ON expression states in each tissue) using well-established phylogenetic models of discrete-trait evolution.

In this study, we present a pipeline that integrates zigzag with phylogenetic comparative methods to infer the evolutionary dynamics of transcriptome turnover. To test this pipeline, we used RNA-seq datasets from 2 male reproductive organs, testes and seminal fluid-producing accessory glands (AGs), across 11 Drosophila species. We chose these organs as the test subject for our approach because previous studies have shown that the male reproductive system evolves faster than other tissues at the level of both protein sequence (Begun et al. 2000; Kern et al. 2004; Haerty et al. 2007; Patlar et al. 2021) and gene expression (Meiklejohn et al. 2003; Ranz et al. 2003; Rifkin et al. 2003; Zhang et al. 2007; Pal et al. 2023), suggesting that we may be able to estimate the rate of transcriptome turnover even with a limited number of taxa.

With this analysis pipeline we were able to obtain estimates of the rate of transriptome turnover for each organ, how much those rates correlate between the 2 organs, the degree of punctuated evolution or “burstiness” of turnover, and how much rates vary among different gene families and functional categories. Our results suggest that qualitative gains and losses of gene expression are fairly common in these organs, and that the rates of turnover vary over time. We discuss the possible implications of widespread activation and silencing of genes for organ evolution. We also discuss a number of important challenges and pitfalls associated with this approach.

## Results

### Transcriptomes of Testes and Accessory Glands in 11 *Drosophila* Species

**Fig. 1.**
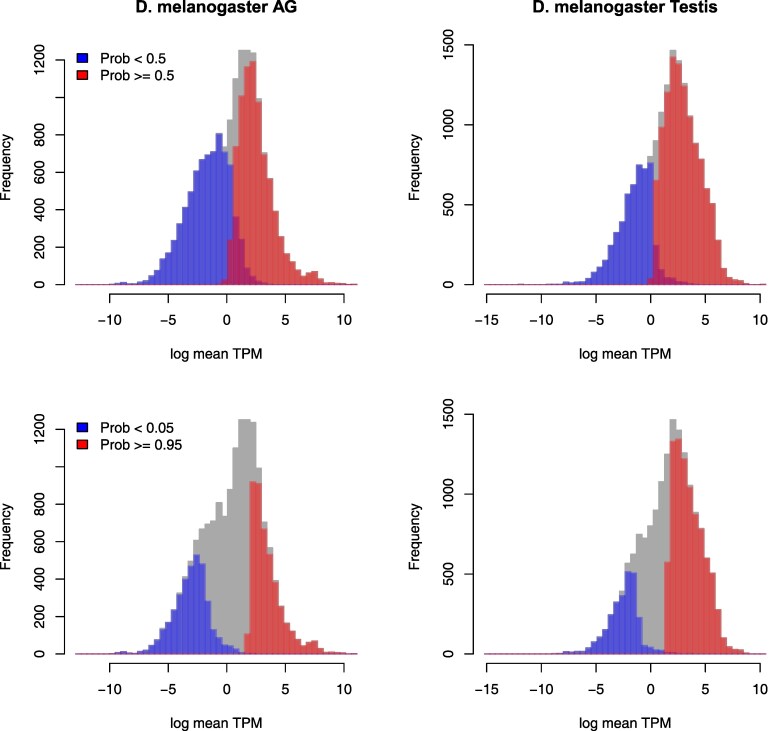
Probabilistic estimation of discrete expression states from continuous RNA-seq data using *zigzag* at 2 different probability cutoffs. Top row: genes with posterior probabilities of active expression P<0.5 are assigned to the inactive state (blue), while those with P>0.5 are assigned to the active state (red). Bottom row: P<0.05 are inactive and P>0.95 are active.; genes with intermediate probabilities are not assigned to either active or inactive states. Similar estimation was performed for all species, separately for each tissue, under a range of probability thresholds. Gray shows the combined frequency of genes classified as active, inactive or neither. As the probability cut-offs become more stringent, the overlap between active and inactive distributions is reduced, but a larger fraction of genes remain unclassified.

We sequenced the transcriptomes of testes and accessory glands in 11 species of the *Drosophila melanogaster* species group (see Materials and Methods). We used *zigzag* (Thompson et al. 2020) to assign genes to active or inactive expression states in the testes and accessory glands of each species under multiple probability thresholds. *zigzag* jointly estimates a probability of active expression for all genes thus producing a distribution of gene-specific marginal posterior probabilities of active expression. All genes with probabilities above an upper threshold were classified as active while those below a lower threshold were considered inactive. As an example, Fig. 1 (top) shows the estimation performed for *D. melanogaster* at P<0.5 for the inactive and P>0.5 for the active state.

**Fig. 2.**
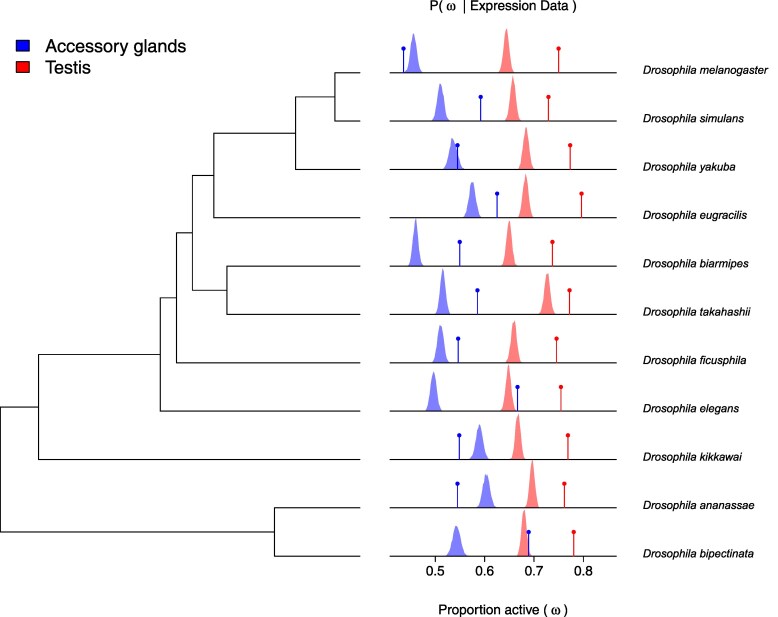
Posterior distributions of the weight active parameter in the *zigzag* model (*ω*), which measures the proportion of all protein-coding genes that are actively expressed in the AG (blue) and testes (red) of each species; species phylogeny is shown on the left. Vertical lines indicate the expected proportion of single-copy genes (out of n=8,660) that are active in each organ, calculated from the posterior probabilities of active expression of each gene in each species. If the expression states of genes were, on average, the same for single-copy and non-single-copy genes, the peaks of the weight active posterior distributions would coincide with the vertical lines. In most cases, however, a higher proportion of single-copy genes is estimated to be active, compared to the total protein-coding genome.

Inference under the *zigzag* model suggests that a larger proportion of the genome is expressed in the testes compared to the AG (Fig. 2), which supports recent findings (Cridland et al. 2020). The inferred proportion of expressed genes is 44–59% in the AG and 63–74% in the testis (lowest 2.5% quantile to highest 97.5% quantile among species posterior distributions). In most cases, a higher proportion of single-copy genes (gene families containing a single gene in all species) are actively expressed, compared to the rest of the genome (Fig. 2). The single-copy set is likely enriched for housekeeping genes, which are broadly expressed and tend to have less copy number variation in populations than other genes (Dopman and Hartl 2007; Henrichsen et al. 2009).

The fraction of transcripts coming from inactive genes is similar in the 2 organs. Averaging over the posterior distribution of *zigzag*’s latent mixture model implies that inactive genes contribute <1% of the total mRNA in each transcriptome. Specifically, the percentage ranged from 0.3 to 0.5% of the AG transcriptome and 0.2–0.3% of the testis transcriptome among all species. We estimated the threshold level of expression at which a gene is, on average, as likely to be active as inactive (probability of active expression P=0.5). Depending on the species, this threshold ranges from 1.0 to 2.1 TPM in the AG and 1.0 to 1.8 TPM in the testis. For reference, in a homogeneous cell mixture, if the number of mRNA transcripts in each cell is on the order of $10^{5}-10^{6}$ (Islam et al. 2014), then a gene with average expression of 1 TPM will have one or more transcripts in about 10–60% of cells—assuming binomial sampling. Our variable threshold estimates, which are similar to those obtained by other methods in other tissues and organisms (Wagner et al. 2013; Costa et al. 2022), confirm that no single “hard” cut-off of TPM or FPKM values would be equally appropriate for all species and tissues (Thompson et al. 2020).

**Fig. 3.**
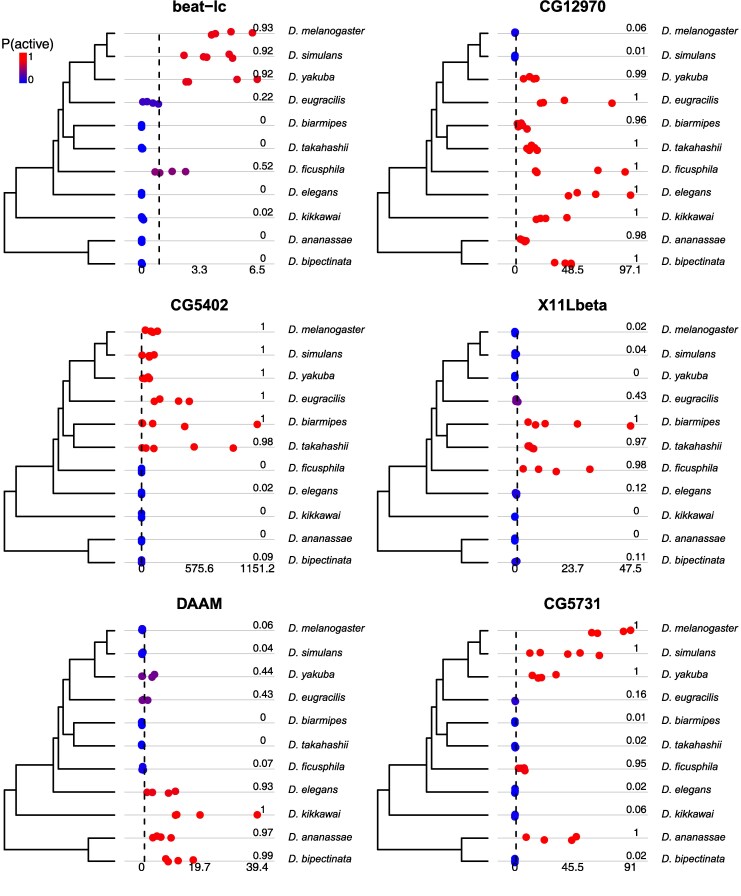
Comparative expression levels and expression state estimates for 6 genes in accessory glands that show one or more evolutionary changes in expression state among the 11 species. Dot plots show the estimated TPM values for each of the 4–5 biological replicate RNA-seq libraries in each species, with the range of TPM values indicated under the dot plots. TPM=1 is indicated by a vertical dashed line. Hot and cold color gradient and numbers to the right of the dots show posterior probability of active expression under the *zigzag* model.

Inference under the model also supports previous findings that the testis and AG transcriptomes are highly overlapping (Cridland et al. 2020). Under a more stringent threshold where genes with probability of expression between 0.1 and 0.9 were classified as unknown, we found that the AGs and testes share between 4,681 and 6,682 actively expressed (P>0.9) genes, depending on the species. The 2 organs differ in the number of exclusive genes, i.e. those actively expressed in one but not the other organ. The AGs express between 81 and 189 exclusive genes per species, while the testes are more variable with 287 and 1,887 exclusive genes per species (Table 1). This supports previous studies in *D. melanogaster*, *D. yakuba*, and *D. simulans* showing that the testes express an elevated number of tissue-specific genes relative to other tissues, including the AGs (Cridland et al. 2020; Takashima et al. 2023). These are likely minimum estimates due to our conservative threshold for active and inactive expression calls. Our *zigzag* analyses confirm that the set of genes that are active in a transcriptome vary not just among organs but between species (Fig. 3).

**Table 1.** The transcriptomes of testes and accessory glands

	AG	Testis
% active protein-coding genes	44–59%	63–74%
% transcripts from inactive genes	0.3–0.5%	0.2–0.3%
TPM of genes with P=0.5 active	1.0–2.1 TPM	1.0–1.8 TPM
Number of exclusive expressed genes	81–189	287–1,887

### Expression of X-linked Genes in Male Reproductive Organs

**Fig. 4.**
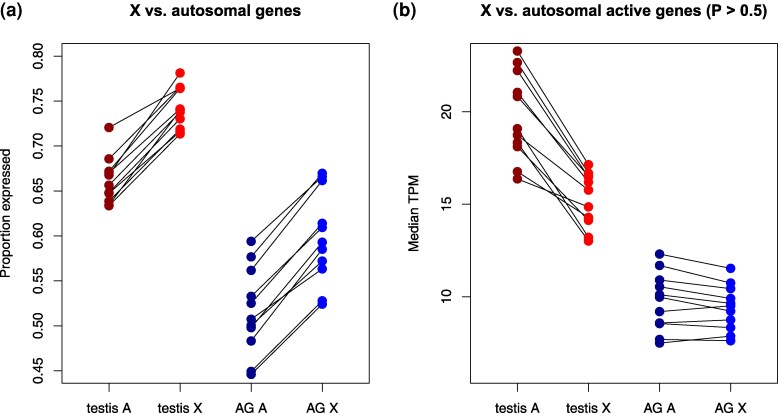
Comparison of gene expression between the X chromosome and autosomes. a) Each colored dot shows the expected proportion of actively expressed genes (averaged over the posterior probability of expression) in a given species and organ (testis shown in red; AG shown in blue.). Lines connect the autosomal (“A”) and X-linked categories within each species. b) Lines connect the median expression levels of actively expressed genes for autosomal and X-linked genes within each species. Genes are considered “active” if their probability of expression is >0.5.

Previous studies have found that the X chromosome is depleted for genes with male-biased expression (Parisi et al. 2003; Ranz et al. 2003; Mueller et al. 2005; Sturgill et al. 2007; Mahadevaraju et al. 2021). In the genus *Drosophila*, genes with testis-biased expression show a tendency to move from the X to the autosomes (Vibranovski et al. 2009), while accessory gland proteins (Acps) are significantly under-represented on the X chromosome in *D. melanogaster* (Ravi Ram and Wolfner 2007). In these studies, tissue-specific (or tissue-enriched) genes were identified on the basis of expression bias, i.e. quantitative difference in transcript abundance between different organs or between males and females. These studies reveal how organs and sexes are represented on the X chromosome, but not necessarily how the X is represented in the transcriptome of each organ. We found that a higher proportion of X-linked genes are expressed in both testes and accessory glands (AGs) compared to autosomal genes (Fig. 4a). However, X-linked genes expressed in the testes have about 30% lower median expression levels than their autosomal counterparts (Fig. 4b). These results are also consistent with the findings of Mahadevaraju et al. (2021; Fig. 1j). In contrast, we observed little difference in AG expression. These findings suggest that while male-specialized genes are under-represented on the X, a greater fraction of X-linked genes are active in male sex organs, albeit with lower expression levels in the testes (Mahadevaraju et al. 2021; Witt et al. 2021; Wei et al. 2024).

### Phylogenetic Model of Transcriptome Turnover

To investigate the evolutionary processes that contributed to the patterns we observe in our data, we fit a model of transcriptome evolution in the 2 organs to a time-calibrated phylogenetic tree and the expression states for single-copy genes in both organs (see Methods). In brief, we assumed that each gene’s expression state evolved as a two-state (active/inactive) continuous-time Markov chain (CTMC) with turnover rate (rate of activation/deactivation of expression) varying across branches according to an uncorrelated relaxed clock model where each branch of the tree was assumed to have a unique mean turnover rate that correlated between the 2 organs. We also included among-gene rate variation in our model to account for differences in turnover rate among genes.

Ideally, we would average over the uncertainty of the expression states of all genes while fitting evolutionary models to the *zigzag* posterior probabilities. Unfortunately, such phylogenetic analysis methods are not currently available for transcriptome-scale comparative data, which involve thousands of characters. We therefore set 2 probability thresholds derived from a single value we call *α* to classify genes as active (high probability of active expression; >1−α), inactive (low probability of active expression; <α), or unknown/missing data (intermediate probability of active expression; between *α* and 1−α). Because, to our knowledge, this type of study has not been performed before, it was important to investigate the consistency, adequacy, and robustness of not just the *zigzag* predictions of expression state, but also of the evolutionary model fit to those inferred expression states. The probability cutoffs for active and inactive genes in particular can potentially have a large impact on inferences. If these cutoffs are too conservative, too much data will be thrown out and the prior will have a strong impact on our inferences. As the cutoffs approach 0.5, more of the data is used for inference but the number of misclassifications increases, which could overpower the true signal in the data.

To assess the overall adequacy of our evolutionary model and to select appropriate probability cutoffs for gene expression states, we conducted a series of cross-validation experiments and sensitivity tests (see Methods and supplemental text S1, Supplementary Material online). The results of this cross-validation experiment revealed that the probability thresholds of α=0.05,0.1, and 0.25 work well for our data such that phylogenetic inferences of the expression states of the hold-out data agreed with the *zigzag* estimates with probability near 0.88 on average (see supplemental text S1, figs. S5–7, Supplementary Material online). Accuracy was similar for each species (supplementary fig. S6, Supplementary Material online). We performed phylogenetic analyses under all cutoffs, and mostly report the estimates based on an α=0.1 as our most confident estimates since that level had the highest average accuracy.

### History and Dynamics of Transcriptome Turnover

Inferences under our model suggest distinct modes of evolution for the 2 organ’s transcriptomes. Our model allowed us to directly infer the correlation of branch-specific rates of transcriptome turnover in the 2 organs. Despite there being a great deal of uncertainty about the strength of this correlation (95% highest posterior density interval (HPD) for the correlation parameter is [−0.05, 0.76]), the correlation is likely no >0.76.

**Fig. 5.**
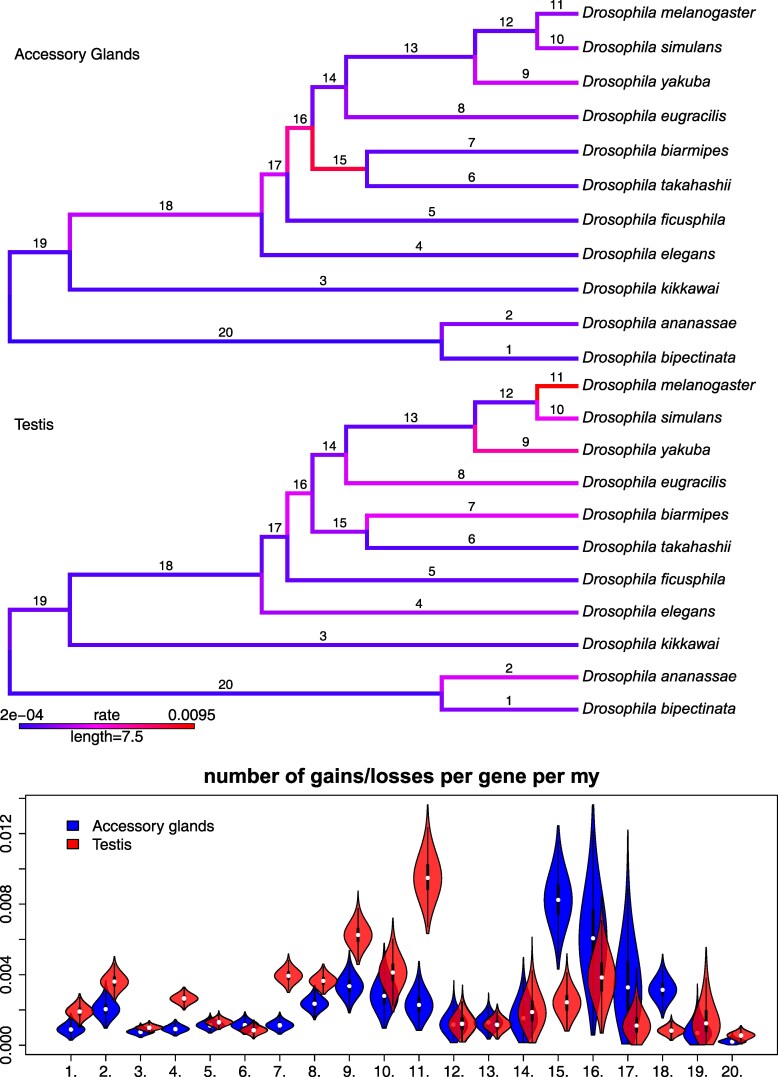
Posterior mean estimates of branch-specific relative turnover rates for single-copy genes in the AG (top phylogeny) and testis (bottom phylogeny). Branch color reflects the mean posterior estimates of the transcriptome turnover rate (changes per single-copy gene per MY), from low (blue) to high (red). The bottom violin plot shows the full posterior samples of the relative turnover rate for AG (blue) and testis (red) for each branch numbered on the phylogenetic trees. Note the accelerated turnover on branch 11 in the testis and branches 15–18 in the AG. The Oriental lineage is descended from branch 18.

We estimated the rates at which genes were turned on and off in the AG and testis and explored how those rates varied among genes and organs as well as among the different branches of the species phylogeny. Assuming that the root age of the species tree is approximately 25 million years (Obbard et al. 2012), our posterior estimates of branch rates suggest that the accessory glands experienced a pulse of rapid transcriptome turnover around 10 million years ago, near the base of the Oriental lineage of the *melanogaster* species group (Fig. 5). In contrast, we infer much more subdued rates of turnover in the testis on those branches. Also, in contrast to the AG, we infer accelerated transcriptome turnover in the testis on the *D. melanogaster* tip branch, compared to the rest of the tree (Fig. 5, branch 18). For probability cutoffs (*α*) of 0.05, 0.1, and 0.25, the relative rates of turnover were fairly robust to the choice of probability cutoff, with branches 15 and 16 near the base of the Oriental lineage consistently showing the highest rates of turnover for accessory glands, while some terminal branches consistently show elevated turnover in testes, in particular in *D. melanogaster* (supplemental text S1 figs. S1–S4, Supplementary Material online).

**Fig. 6.**
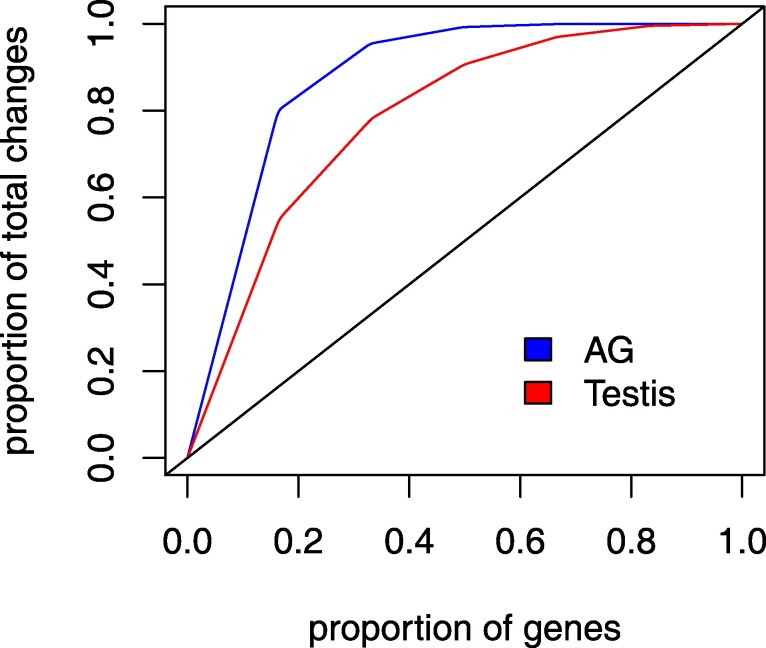
Transcriptome turnover is concentrated among a minority of genes. Proportion of single-copy genes (n=8,660) are ranked on the X axis by their rate of change, from fastest to slowest evolving. The red (testis) and blue (AG) curves show the cumulative proportion of transcriptome turnover events explained by each subset of genes. For example, 20% of genes account for nearly 80% of all turnover in the AG. The black line shows the expected curve if all genes turned over at the same rate.

At the cutoff of α=0.1, we estimate that the mean gene expression turnover rate is $1.6\times 10^{-3}$ per million years in the AG and $2.0\times 10^{-3}$ per million years in the testis (Table 2; supplementary Data file S2, Supplementary Material online). Overall, the per-gene turnover rate appears to be on the order of $10^{-3}$ per MY (Table 2). For reference, this is on the same order of magnitude as the per nucleotide rate of substitutions in *Drosophila*, where estimates are around $8\times 10^{-3}$ per MY (Obbard et al. 2012). Thus, for a gene of size 1 kb, we expect it to change expression state on the order of once every 1,000 base changes. For a genome of around 15,000 genes, these turnover rates imply that roughly 40 expression activation/deactivation events happen in each organ per million years. However, this does not imply a massive rewiring of the transcriptome. Most expression turnover is likely driven by frequent changes in the expression state of a relatively small subset of genes (Fig. 6).

**Table 2.** Mean turnover rates per gene for AG and testis at different probability cutoffs (*α*)

Probability cutoff (*α*/1−*α*)	AG mean turnover rate per gene (my^−1^)	Testis mean turnover rate per gene (my^−1^)
0.5	$5.8\times 10^{-3}$	$3.8\times 10^{-3}$
0.25/0.75	$3.1\times 10^{-3}$	$2.9\times 10^{-3}$
0.1/0.9	$1.6\times 10^{-3}$	$2.0\times 10^{-3}$
0.05/0.95	$1.0\times 10^{-3}$	$1.5\times 10^{-3}$
0.01/0.99	$0.4\times 10^{-3}$	$0.7\times 10^{-3}$

To estimate the variation among genes in contributing to transcriptome turnover, we analyzed the posterior distribution of the parameters of the among-site rate variation model. This model draws gene evolution rates from a discretized gamma distribution (see Methods). In the accessory glands, we estimate that half of all transcriptome turnover of single-copy genes is being driven by about 10% of the genes, while about 15% of genes are driving half of all changes in the testis. Because these estimates come from single-copy genes, which appear to be more likely to be expressed in the AG and testis compared to the rest of the genome (Fig. 2), these rates of turnover probably differ from the genome average and, we suspect, underestimate the average rate for all genes. On the other hand, transcriptome turnover may be slower in other tissues compared to the rapidly evolving male reproductive system. In summary, our analyses imply that the rate of transcriptome turnover changes over time independently in the 2 organs and is highly variable among genes.

**Fig. 7.**
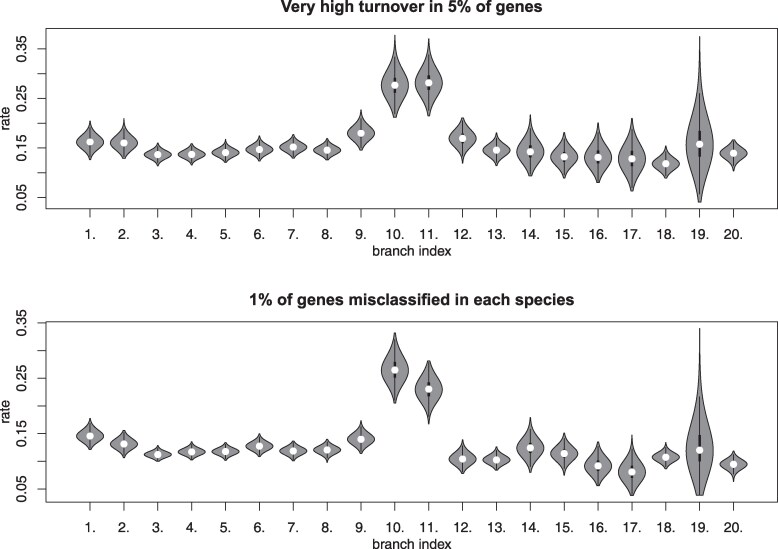
Posterior distributions of branch rates for data simulated under 2 scenarios where the true branch rates are constant. See Fig. 5 for branch numbers. Top panel shows inferred branch rates when 5% of genes change expression states 100 times more rapidly than other genes. Bottom panel shows the inferred branch rates when 1% of genes have their expression states misclassified. Note that branches 10 and 11 are the shortest terminal branches in the tree.

We investigated 2 plausible artifacts that could cause the inferred patterns of branch rate variation in these 2 organs. One potential artifact could be caused by a subset of genes exhibiting a very high rate of turnover, such that phylogenetic information is quickly erased to different degrees in different parts of the tree, e.g. longer branches vs. shorter branches near the tips. A second artifact could be caused by our method of classifying genes as ON or OFF. Our inferences could be highly sensitive to misclassification, which could lead to higher estimates of turnover rates near the tips of the tree. To explore these potential sources of error, we simulated 2 datasets under these scenarios using *sim.char* in the R package *geiger* v 2.0.11 (Pennell et al. 2014), with the same number of genes and species and the same tree as the *Drosophila* dataset. In both simulations, activation rate equaled deactivation rate and branch rates were held constant in order to test whether these potential problems could lead our method to infer significant branch rate variation where none exists. In the first simulation, all genes evolved at the same rate, except 5% of genes evolved 100-fold faster. In the second simulation, there was no variation in rates among genes, but 1% of the genes in each species and organ had their expression state flipped to simulate misclassification. Figure 7 shows that the branch rates at shorter terminal branches in our model are sensitive to both scenarios. Thus, the elevated rates observed on branches 1, 2, 9, 10, and 11 may be caused in part by state misclassification, a very high turnover rate in a subset of genes, or both.

### Turnover Rate and Biological Function

**Fig. 8.**
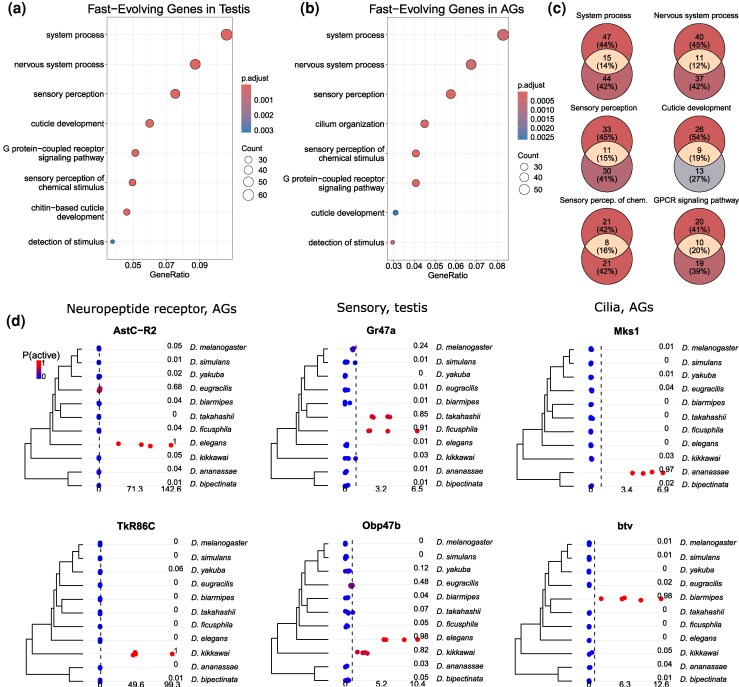
Genes with elevated turnover rates are enriched for similar functional categories in testes and AGs. a) A dot plot from a gene ontology (GO) analysis showing all significantly enriched (*q*-value <0.05) biological process terms among the genes showing rapid turnover in the testis. Genes are designated as showing rapid turnover if they have rates at least two-fold higher than the mean. The list of all singleton genes expressed in the testis is used as the background. b) The top 8 significantly enriched terms in the AG; analysis performed the same way as in (a). The full list of significantly enriched AG terms can be found in supplementary fig. S8 of supplemental text S1, Supplementary Material online. c) Venn diagrams illustrating the overlap between the genes showing rapid turnover in the testis and AG, in each of the shared enriched GO terms. Note that most individual genes are exclusive to one organ. d) Dot plots showing the TPM values for selected genes showing high turnover rates, mapped onto the species phylogeny. Two genes per functional class (neuropeptide receptors, sensory perception, and cilium organization) are shown. For each pair, both genes are shown in the same organ. Dots are colored in accordance with the posterior probability of active expression inferred from the zigzag model. Separate dots within a species represent different biological replicates.

We tested whether the genes that show elevated turnover rates in male reproductive organs are enriched for particular biological functions. To do this, we performed gene ontology (GO) analysis on the sets of genes that showed turnover rates at least two-fold higher than the mean. This analysis was done separately for AGs and testes, using all singleton genes expressed in the corresponding tissue as the background. Eight GO terms in the testis, and 20 in the AG, showed significant enrichment (Fig. 8a, b; and supplementary fig. S7 in supplemental text S1, Supplementary Material online). There was strong overlap in the significant terms between the 2 tissues, such that all gene categories that were enriched in the testis were also enriched in the AG. However, there was only a limited overlap between the individual genes driving the enrichment (Fig. 8c). The enriched GO terms were related to sensory perception (including olfactory and gustatory receptor genes and odorant-binding proteins), GPCR signaling (especially neuropeptide signaling), and cuticle development. The AG additionally showed enrichment for terms related to cilium organization, membrane ion transport and membrane projection.

The pattern of unusually fast turnover could potentially be explained by contamination of some samples with RNA from non-target tissues. For example, the presence of genes related to cilium organization in AG samples could reflect contamination from sperm, transcripts related to cuticle development could be coming from epithelial tissues, and genes related to GPCR signaling and sensory perception may reflect contamination from neurons that innervate the reproductive organs. However, several lines of evidence argue against contamination being a major factor. First, other genes that are highly expressed in the potential sources of contamination (such as ion channels and sperm-specific genes) do not show up in the enrichment analysis. Second, the inferred gains of expression are generally consistent across replicates of the same species (Fig. 8d). Third, these gains are often associated with high TPM counts (Fig. 8d). Fourth and most important, different genes in the same GO category show expression gains in different species (Fig. 8d), whereas contamination would produce a highly correlated gain. For these reasons, contamination is unlikely to be a major contributor to the rapid transcriptome turnover we observe.

We do not know what roles, if any, the genes undergoing rapid turnover play in male reproductive organs, but some enriched terms are consistent with their physiology. Neuropeptides are known to regulate copulation, for example, by coupling mating duration to the transfer of sperm and seminal fluid (Taylor et al. 2012). Similarly, the ability of males to upregulate sperm production in response to the presence of females depends on the ability of somatic cyst stem cells of the testis to respond to neuronally secreted octopamine (Martin-Diaz and Herrera 2024). It is conceivable, therefore, that co-option of genes involved in neuropeptide signaling could lead to lineage-specific changes in inter-organ communication or the regulation of secretory cell activity—changes that may, for example, have implications for the males’ ability to dynamically respond to the sociosexual environment (e.g. Hopkins et al. 2019).

### Turnover of Transcription Factors

Differences in the rate of expression evolution among classes of genes can suggest possible mechanisms for phenotypic evolution. In particular, gain or loss of a transcription factor expression can have large downstream consequences on the transcriptome and thus on the function of an organ. If a transcription factor is lost or gained by a transcriptome it may bring with it downstream targets, resulting in a pulse of transcriptome turnover in a lineage on the phylogeny. We examined the mean difference in the turnover rate between transcription factor genes and the rest of the single-copy gene families in our phylogenetic study. To accomplish this we analyzed the posterior distribution of gene-specific rates from the among-gene rate variation model (see Methods). Our data and models imply that transcription factors change expression states at different rates in the accessory glands and testes, where they evolve at a relatively slow rate in the former and a relatively fast rate in the latter. We report the posterior credible intervals of the percent difference of the mean rate (PDMR) between each group of genes (i.e. TF and non-TF). PDMR is calculated with the following formula:


(1) $$\text{PDMR}=\left(\frac{\text{mean TF rate}-\text{mean non-TF rate}}{\text{mean non-TF rate}}\right)\times 100$$

**Fig. 9.**
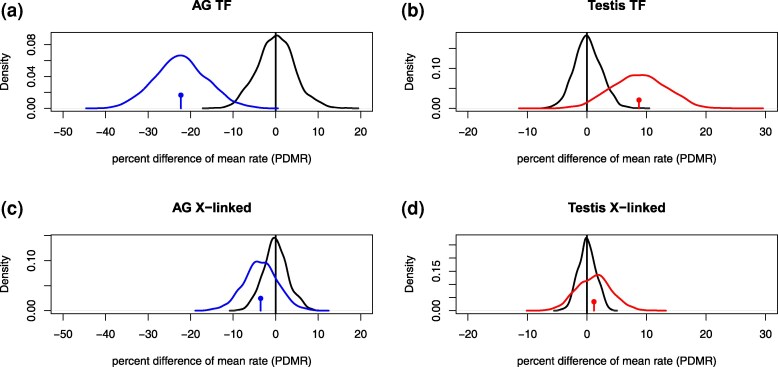
The posterior distributions of the percent difference of mean rate (PDMR; equation 1) for transcription factors and non-transcription factors (top row; A and B) and between X-linked and autosomal genes (bottom row; C and D) in AG (blue) and testis (red). a) The blue distribution gives the percent difference of the mean rate of TF genes and non-TF genes computed from the posterior sample from the phylogenetic MCMC. As a null reference, the black distribution of mean PDMR (M-PDMR) of 1,000 random partitions of all genes into 2 groups equivalent in size to the TF/non-TF partition. The blue point with a line indicates the mean of the distribution. b) shows the same analysis in the testis. c) shows the same analysis but for X-linked/autosomal PDMR. d) is like C except in the testis.

Our models and the RNA-seq data imply that expression states of single-copy transcription factors in AGs turn over on average 10–34% slower than those of non-TF single-copy genes. Conversely, TF expression states in testes turn over 0–17% faster compared to non-TF genes (colored lines and circles in each density in Fig. 9;a–b, blue and red curves). To check how frequently random subsets of genes deviate from the genome average at least this strongly, we measured the mean PDMR (M-PDMR) for each comparison and created an empirical null distribution from random partitions of the data where we summarized each random partition with the same statistic, the M-PDMR (Fig. 9;a–b, black curves). Results show that for TF genes, this statistic is extreme relative to the reference set in both AGs and testis, with < 0.1% of genes in the reference set having more extreme statistics for both organs. Thus, at the level of discrete ON/OFF transitions, our analysis implies that TF genes evolve slightly faster than non-TF genes in the testis, but significantly slower than non-TF genes in the AG.

### Turnover of X-linked Genes

X-linked genes may evolve differently from autosomal genes due to the differences in selective pressures and effective population size that the X chromosome experiences in males vs females (Parisi et al. 2003; Ravi Ram and Wolfner 2007; Singh and Petrov 2007; Sturgill et al. 2007; Vibranovski et al. 2009; Khodursky et al. 2020). We tested whether transcriptome turnover rate differs between X-linked and autosomal genes using the same approach as described above for transcription factors. We found that both classes of genes turn over at similar rates. The posterior distribution of PDMR overlaps zero and places little probability on differences greater than about 10% in either organ (Fig. 9; c–d, blue and red curves). For X-linked genes, the reference distribution of mean PDMR suggests little surprise at seeing this pattern (Fig. 9; c–d, black curves) when comparing gene partitions that are random with respect to X-linkage. In summary, the 2 male reproductive organs show little evidence of either slower or faster evolutionary turnover in the expression of X-chromosomal genes compared to other single-copy gene families.

### Ancestral Transcriptomes of Testes and Accessory Glands

We used our phylogenetic model to estimate the posterior probability that each single-copy gene was expressed in the testes and AGs of the most recent common ancestor of the 11 species in our analysis (i.e. the most recent common ancestor of the *melanogaster* species group). Our model and data imply, at a probability cutoff of 0.95, that 61% of the single-copy genes were expressed in the ancestral accessory gland, and 74% were expressed in the ancestral testis. We also estimated the posterior probability that each gene is actively expressed in *D. melanogaster* but was not expressed in the most recent common ancestor (i.e. that it gained expression in the testis or AG in the *D. melanogaster* lineage over the last ∼25 MY) by analyzing the joint posterior distribution of the expression state at the root node of the tree and at the *D. melanogaster* tip (supplementary Data file S3, Supplementary Material online). This analysis implies that at a probability >0.95, *D. melanogaster* expresses 19 single-copy genes in the AG that were not expressed in that organ in the most recent common ancestor of the *melanogaster* species group, while 95 such genes are expressed in the *D. melanogaster* testis.

**Fig. 10.**
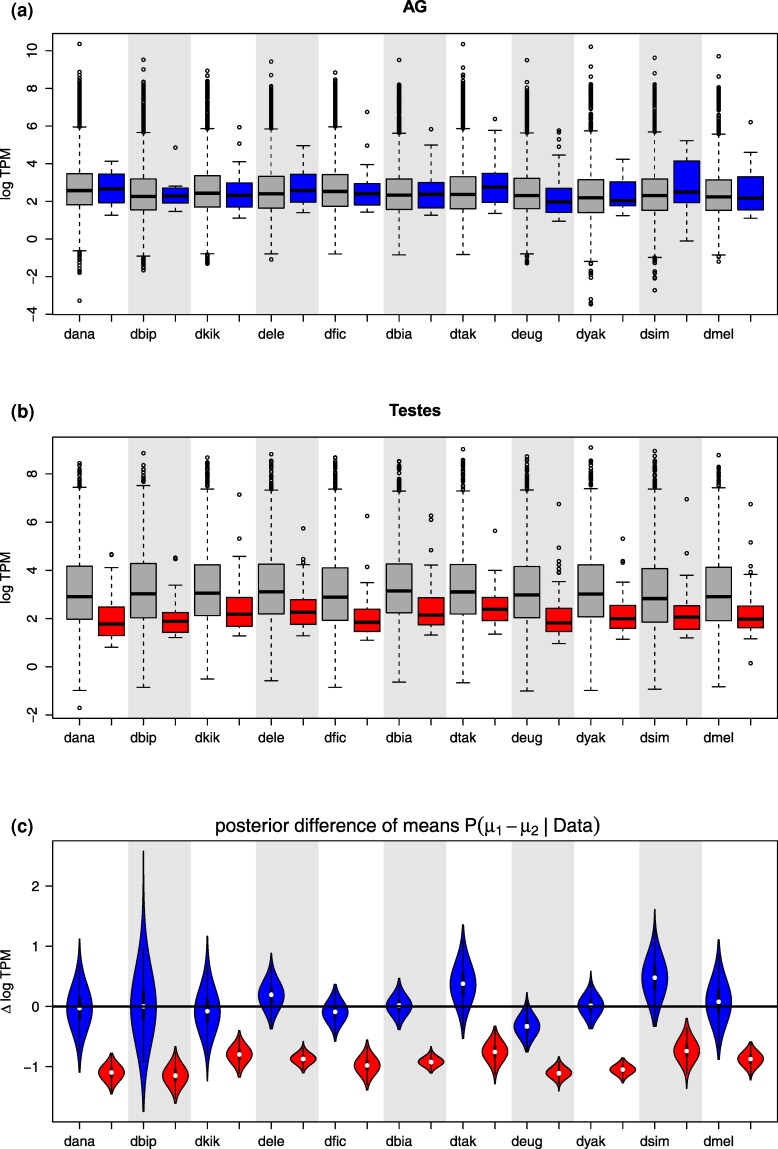
Expression of recently activated genes compared to genes with conserved expression. a,b) “Recently activated” is defined as having >0.95 probability of being OFF in the common ancestor and ON in species X (A; blue boxes for AG, B; red for testes). “conserved expression” is defined as having >0.95 probability of being ON in the common ancestor and ON in species X (gray boxes). c) The posterior distribution (proportional to violin width) of the difference in log TPM between the 2 groups ($\mu_{1}$ is the mean of the recently activated group and $\mu_{2}$ is the mean of the conserved expression group).

These results raise several questions, in particular—how big are the changes in transcript abundance associated with the gain of active expression? Do recently activated genes remain expressed at levels barely above our probability cut-offs, or does their expression approach the levels typical of other genes expressed in that tissue? To address this question, we compared the expression levels of newly active and ancestrally active genes in each species in both organs (Fig. 10a,b). Newly active genes were defined as those that have >0.95 probability of being ON in the focal species and >0.95 probability of being OFF in the most recent common ancestor of the 11 species in our study. Conserved, ancestrally active genes are defined as those with >0.95 probability of being ON both in the focal species and in the common ancestor. We estimated the posterior distribution of the difference in mean log TPM between these 2 groups using the R package BEST v 0.5.4 (Kruschke 2013) setting default vague priors (Fig. 10c). Resulting inferences imply that the 2 organs differ in the expression levels of newly active genes. In testes, such genes have lower expression compared to ancestrally active genes, while in the AG both classes of genes are expressed at roughly similar levels (Fig. 10). Thus, in at least some tissues newly activated genes approach typical expression levels. In fact, some recently activated genes are expressed at high TPM (Fig. 3,Fig. 8d).

## Discussion

In this report, we explored the feasibility of using discretized RNA-seq data to study the evolutionary turnover of transcriptomes—that is, the gain and loss of tissue-specific expression by conserved genes. We created an analysis pipeline that fits discrete trait evolution models to comparative RNA-seq datasets. This pipeline translates continuous RNA-seq data to discrete trait data using a Bayesian method for expression state inference (*zigzag*; Thompson et al. 2020). To investigate the co-evolution of transcriptomes across multiple organs, we defined a phylogenetic model of correlated gene expression evolution in 2 or more organs. We demonstrated the power of this research pipeline by examining 2 rapidly evolving organs in the *Drosophila* male reproductive system, namely the accessory glands and the testes.

We first characterized attributes of the 2 organ transcriptomes in each species individually. We estimated that testes express a greater proportion of the genome compared to accessory glands, with a proportion of actively expressed genes falling in the 63–74% range in the testis and 44–59% in the AG across the 11 species. The level of background expression noise (i.e. the fraction of the transcriptome that does not correspond to actively expressed genes) is similar between the 2 organs and is below 1% in all species. In other words, >99% of protein-coding transcripts in cells appear to be from actively expressed genes.

With our model of correlated transcriptome evolution, we inferred turnover rates and historical expression states of 8,660 single-copy genes. This inference suggests that the transcriptomes of the two male reproductive organs evolve at similar overall rates but follow distinct evolutionary paths. The accessory glands and testes show bursts of rapid transcriptome turnover at different times and in different parts of the tree. Among genes, there is greater variation in turnover rates in the AG than in the testis, indicating a lower baseline rate of turnover in the AG with a subset of genes showing higher rates of evolution. We inferred a relatively low correlation in branch rate between the organs, though the inferences were highly uncertain. Another type of correlation we did not explore but is worth mentioning is the gene-level correlation between the organs. In our model we sought to capture a dynamic where an acceleration in evolution of one organ may correlate with the rate of evolution of another organ regardless of which genes are experiencing accelerated evolution. We hope in the future this model is expanded to include dynamics where genes can change state in multiple organs simultaneously.

We also demonstrated that we can estimate ancestral transcriptomes with this phylogenetic model. Ancestral state reconstruction methods can be used to estimate tip states for missing data as well. We performed a leave-k-out experiment to obtain estimates of the state of genes at the tips which were “held out” by setting them as missing data. We used these estimates to check the consistency between our evolutionary inferences and the state predictions from *zigzag*. This experiment indicates a solid degree of consistency with an average accuracy of 88%. In like fashion, genes that are truly missing from a dataset either because of missing measurements or because *zigzag* couldn’t identify their states with sufficient certainty can leverage the information in other species through phylogenetic relationships to gain more certainty.

We explored the evolutionary patterns of 2 important classes of genes, transcription factors and X-linked genes, to test whether they follow distinct evolutionary dynamics compared to the rest of the genome. Transcription factor expression appears to evolve faster than the non-TF single-copy genes in the testis, but much slower in the AG. It is interesting to note that our estimates of the overall transcriptome turnover rates are similar for the 2 organs. Given that changes in TF expression are expected to have a large impact on the transcriptome, this is a curious result. One possibility is that the gain and loss of TF expression may modulate the expression levels of genes that are already expressed in the organ, rather than changing the expression states of genes from off to on or vice versa. Alternatively, downstream expression changes that result from TF turnover could be more pronounced in non-single-copy gene families that undergo duplication and deletion more frequently. Finally, we note that the posterior distributions of the mean AG and testis turnover rate are both fairly wide, and thus moderate to large differences in rates are plausible. Analysis among more species could help provide more precise estimates.

Our estimates of evolutionary rates indicate that the “fast-X effect” on expression levels (Meisel et al. 2012b) doesn’t necessarily imply a faster rate of transitioning between ON and OFF among conserved single-copy genes. We find that X-linked genes turn over at rates not significantly different from autosomal genes. However, we emphasize that our dataset consists of conserved single-copy gene families among eleven *Drosophila* species. The fast-X effect may be in part driven by gene families that are more evolutionarily labile. Gene duplication and deletion in some gene families is known to play an important role in expression evolution (Lynch and Conery 2000; Lynch and Force 2000; Thompson et al. 2016, 2018; Ebadi et al. 2023).

*zigzag* allowed us to gain some insight into the representation of the X and autosomal chromosomes in male organs. We found that a higher percentage of X-linked genes is expressed in both organs compared to autosomal genes. How does this result fit with the large body of research on the reverse relationship, that is, how male organ genes are distributed among the chromosomes? Several studies report that genes with testis-biased and AG-biased expression tend to be under-represented on the X chromosome in *D. melanogaster* and other *Drosophila* species, suggesting that X-linked genes with male-specific functions may be disfavored by selection (Parisi et al. 2004; Vibranovski et al. 2009; Mikhaylova and Nurminsky 2011; Assis et al. 2012; Meisel et al. 2012a; Mahadevaraju et al. 2021). This paucity of male-biased genes on the X chromosome does not necessarily imply that the X chromosome is under-represented in any given tissue relative to autosomal genes. These studies also focus on quantitative expression levels rather than expression states and thus cannot estimate the proportion of classes of genes (e.g. X-linked genes) that comprise transcriptomes.

Additionally, we estimate that actively expressed X-linked genes are expressed at around 30% lower levels than autosomal genes in the testis while the 2 types have similar expression levels in the AG. This whole-organ pattern is expected if dosage compensation is decreased or the X is silenced in the male germline (Vibranovski et al. 2009; Meiklejohn et al. 2011; Mahadevaraju et al. 2021; Witt et al. 2021; Wei et al. 2024).

### Challenges and Pitfalls

Different dimensions of the dataset are informative about different model parameters. An obvious question is how this type of analysis is influenced by the number of species, genes and biological replicates per species. The first step in our analysis pipeline estimates the expression state of genes from replicated continuous (TPM) data. Four samples per species appear to be sufficient for *zigzag*, as we find that increasing the number of samples further provides only marginally more precise estimates (Thompson et al. 2020). As for the number of taxa, we found that with just 11 species, two organs, and 8,660 genes, we were able to detect differences in the mode and tempo of transcriptome turnover both between tree branches and between organs. However, many model parameters of interest, such as the correlation of turnover rates between the 2 organs, have posterior distributions that are quite wide. This is also true for the turnover rates of individual genes parameterized in the among-site rate variation model. Datasets containing more species should better resolve these parameters and shed light on the answers to many other important questions relating to transcriptome evolution while allowing for richer and biologically realistic models of regulatory evolution. In summary, gene-level parameter estimates should benefit primarily from more species, while transcriptome-level parameters should benefit from more species as well as from including more genes in the analysis.

The first point of failure in any comparative transcriptome study is dissection of biological samples. Because “comparative” assumes homology, it is crucial that RNA samples are isolated from truly homologous tissues. This is not a trivial problem if the organ system under study changes structure frequently and/or is not defined in a consistent way among species. It is easy to imagine scenarios where it is difficult to distinguish the evolutionary co-option of new genes in a transcriptome from differential contamination by other tissues and cells in a subset of species. We took many precautions with the accessory glands and testis at several steps in our analysis pipeline to detect and mitigate potential artifacts emanating from dissection error. Our analysis of individual gene expression patterns suggests that cross-tissue contamination is not a major source of artifacts in this study. Nevertheless, it is important to consider this potential source of erroneous inference in all comparative transcriptome analyses, whether qualitative or quantitative.

The next set of challenges emerge from how expression is defined and quantified. Similar to comparative analyses that treat gene expression as a continuous character, evolutionary inference of discrete expression states is fraught with potential artifacts emanating from the data processing pipeline (Khaitovich et al. 2005; Harrison et al. 2012; Romero et al. 2012; Rohlfs et al. 2014; Dimayacyac et al. 2023; Pal et al. 2023). The most obvious factors to influence results would be genome assembly and annotation quality. If some species have few reads mapping to an ortholog because the gene was not correctly assembled or given an incorrect annotation, that could obviously influence the inferred expression state and comparative patterns for that gene.

Tip state uncertainty poses another set of challenges that require validation and innovation. The posterior probability of active expression is continuous, which requires discretization for trait models that do not account for tip state uncertainty. This necessitated using a probability threshold, *α*. We conducted a leave-k-out validation experiment to evaluate sensitivity to probability thresholds and selected a conservative threshold for downstream analysis. A more robust method would average over tip state uncertainty during inference (Liebeskind et al. 2019; Beaulieu and O’Meara 2024). Although most genes in our dataset had highly certain states, this may not hold true for many datasets.

An essential assumption for conducting a macroevolutionary analysis is that expression states of genes do not vary among populations in each species, which may not be the case for some genes (Cridland et al. 2020). Our approach to this problem was to sample 2 distinct populations in each species and then run a leave-one-library-out *zigzag* analysis to see which genes are sensitive to the exclusion of individual libraries (see Methods).

Our use of a new method for classifying gene expression states, *zigzag*, requires careful model checking, sensitivity tests, and validation; indeed, posterior predictive checks and cross-validations are essential for any study seeking to combine numerous complex models in an inference pipeline. Our sensitivity and posterior predictive checks and cross-validation experiments suggest some robustness. However, it can be difficult to know how robust the inferences are because when it comes to expression states of genes, ground truths are themselves inferences under models. The fact that we observe an 88% probability of agreement between evolutionary predictions of the expression states of hold-out genes and the *zigzag* predictions of their expression states (supplemental text S1, Supplementary Material online) appears to be a good indication that our analysis is providing a fair approximation of the expression states and evolutionary processes behind the comparative expression patterns in our data. However, interpreting this result requires care, and we encourage others to critically explore potential weaknesses that may exist in this approach.

As in any comparative study, our results depend on the accuracy with which species relationships are inferred. We treat the species tree and root age as known, when in fact these are estimates and thus have some unmodeled level of uncertainty around them. We have high confidence in the tree topology, since it is similar to the phylogeny based on hundreds of loci distributed across the entire genome (Suvorov et al. 2021). On the other hand, the estimates of node ages in *Drosophila* are notoriously uncertain due to the dearth of fossil calibration points (Suvorov et al. 2021). Including this uncertainty may decrease the precision of posterior estimates of branch rates.

Another critical assumption in our methods (as in other analyses of gene expression evolution) is that each gene is evolving independently. This is a convenient simplifying assumption of the phylogenetic inference model. However, it is unknown how much bias emanates from this simplification for transcriptome evolution, where this is obviously not true given the regulatory interactions among genes. If the expression state of the average gene is governed by a complex interaction of numerous genes, then this assumption of independence may be a reasonable approximation. However, if many genes are highly sensitive to the state of a small number of genes, this assumption may result in misleading inferences.

### Future Directions

The first step toward identifying causal mechanisms is to find patterns of correlation. While quantitative changes in gene expression are both highly prevalent and affect adaptive phenotypes, qualitative gain or loss of gene expression can, like gene deletion and duplication, have especially profound consequences for organ structure and function (Gompel et al. 2005; Rebeiz et al. 2009; Chan et al. 2010; Fraser et al. 2010; Arnoult et al. 2013; Loehlin et al. 2019; Kowalczyk et al. 2022; Marand et al. 2023). Potential cases of association between genetic and phenotypic change can be identified by phylogenetic analysis: if a gene was activated or inactivated on the branch where a new phenotype evolved, that gene may be part of a tissue-specific regulatory pathway mediating the evolutionary change. In this study, we took a step toward a systematic comparative analysis of gene expression states by developing a method for quantifying the rate of transcriptome turnover on phylogenies. In the future, we can build upon this foundation to map the transitions between active and inactive gene expression states to specific branches of species trees. This approach is likely to provide new insights into the regulatory mechanisms driving phenotypic evolution.

This study focuses only on single-copy gene families, which comprise slightly over half of the genome and may not be representative of the transcriptome as a whole. Gene families with unstable sizes (those undergoing frequent gene duplications and losses) may also be subject to faster regulatory turnover (Lynch and Conery 2000; Lynch and Force 2000; Makino and McLysaght 2010; Thompson et al. 2016; Ebadi et al. 2023). This is perhaps one of the greatest weaknesses of evolutionary studies that require filtering the dataset to single-copy gene families. As more species are added, the dataset becomes more enriched for evolutionarily stable gene families. This means as the power of our inferences increase, the generality of those inferences likely decreases. To fully understand how organ function evolves, it is essential to characterize the role of structural changes to the genome in transcriptome turnover. This means we should develop models that integrate evolutionary processes that add and remove genes from the genome with regulatory processes that turn genes on and off. Though challenging, solving this problem will uncover complex relationships, such as those between gene duplication and expression changes or regulatory inactivation and gene loss. As genome annotations improve, expression estimation becomes more precise, and new evolutionary models are developed, we anticipate many novel and intriguing questions will arise from expression state evolution research.

## Methods

### RNA Sequencing and Expression Estimation

*D. melanogaster* testis expression estimates were obtained from Thompson et al. (2020). All other RNA-seq libraries from testes and accessory glands were created and analyzed using the same methods as in Thompson et al. (2020). In brief, mRNA from each organ was extracted from approximately 25 mixed-stage adult male flies. At least 2 biological replicates each from the genome reference strain (used for genome assembly and annotation) and from a second, non-reference strain were obtained. Sequencing was performed on either a HiSeq2500, 3,000, or 4,000 (see the supplemental methods S2.3.2, Supplementary Material online of Thompson et al. 2020). We mapped paired-end reads to published genome annotations (see Table 3) using STAR v. 2.5.3 (Dobin et al. 2013). We assembled reference-only transcripts using Stringtie v. 2.1.4 (Pertea et al. 2015) and estimated transcript abundance in transcripts per million (TPM). Suppl. table S1, Supplementary Material online provides more details about samples.

**Table 3.** Genome and annotation information

Species	Genome release
*D. melanogaster*	r6.12
*D. simulans*	r2.02
*D. yakuba*	r1.05
*D. eugracilis*	Deug_2.0
*D. biarmipes*	Dbia_2.0
*D. takahashii*	Dtak_2.0
*D. ficusphila*	Dfic_2.0
*D. kikkawai*	Dkik_2.0
*D. elegans*	Dele_2.0
*D. ananassae*	r1.05
*D. bipectinata*	Dbip_2.0

### Expression State Inference

We used the R package *zigzag* (Thompson et al. 2020) v.1.0.0 to infer gene expression states from TPM values from multiple biological replicates (libraries) of each tissue and species. *zigzag* assumes that inactive and active genes are drawn from distinct distributions in a mixture model, which are approximately normal and overlap to varying degrees depending on the tissue and species. The model also assumes that individual libraries (biological replicates) are noisy samples from the combined inactive and active distributions of the mixture model. Importantly, *zigzag* assumes that the latent true expression state of each gene is shared among all biological replicates. Increasing numbers of libraries therefore increases the certainty about the expression states of genes. Table 4 shows the prior settings assumed for the accessory glands and the testis.

**Table 4.** Summary of zigzag prior distributions

Parameter	Distribution Type	Distribution Parameters	Description
$s_{0}$	Normal	μ=−1,σ=2	Baseline variance in expression
$-s_{1}$	Exponential	λ=2	Influence of expression level on variance
*τ*	Exponential	λ=1	Variation in gene-specific variance
$\alpha_{r}$	Exponential	λ=1/10	Proportional to library dropout rate
$\omega_{a}$	Beta	α=2,β=2	Proportion active
$\omega_{a}^{k}$	Beta	α=1,β=1	Proportional within active subcomponent *k*
*ρ*	Beta	α=1,β=1	Spike probabililty (true TPM = 0)
$\mu_{a1}-1$	Exponential	λ=1/3	Mean of active component a1
$\mu_{a2}-4$	Exponential	λ=1/3	Mean of active component a2
$\mu_{a3}-6$	Exponential	λ=1/3	Mean of active component a3 (testis only)
$-\mu_{i}$	Exponential	λ=1/3	Mean of inactive component
$\sigma^{2}$	logUniform	log(min)=log(0.01) ,	Variance parameter shared by
		log(max)=log(5)	all mixture components

We updated *zigzag* (v1.0.0) so that all mixture components could share a single variance parameter. This significantly improves MCMC convergence and efficiency with apparently little impact on inference for our samples. Additionally, the previous default model (v0.1.0) allowing the variances to vary between inactive and active components puts positive prior probability on unrealistic models where, for example, if the variance of the inactive component is much higher than the active component(s), genes with very high expression could be assigned to the inactive component. Two independent MCMC chains were run for 25,000 cycles, sampled every 5 cycles, and compared for convergence by measuring PSRF (Brooks and Gelman 1998). A PSRF close to 1 and < 1.2 is considered a good convergence. All parameters for both organs had PSRF <1.2.

*Proportion of transcripts from Inactive Genes.* The proportion of protein-coding gene transcripts emanating from inactive genes is computed from the posterior distribution of the latent mixture model implemented in *zigzag*. The model comprises two primary distributions: an inactive distribution characterized by lower mean expression levels and an active distribution with higher mean expression levels.

The inactive distributions is modeled as a spike-and-slab distribution, consisting of a point mass at TPM =0 with proportion of inactive genes in the spike denoted by *ρ* and a log-normal distribution with a log-mean of $\mu_{i}$ for inactive genes with leaky expression (TPM>0). In contrast, the active component is a mixture of one or more log-normal distributions, each with log-mean(s) $\mu_{a_{k}}>\mu_{i}$. All log-normal distributions share a common log-standard deviation parameter, *σ*. For detailed information about the model, refer to the supplementary text, Supplementary Material online of Thompson et al. (2020).

The proportion of protein-coding transcripts originating from inactive genes is computed from the latent mixture model parameters, θ, and is denoted by δ(θ). This proportion is given by the weighted mean of the log-normal component of the inactive distribution divided by the weighted average of the expectation of all components that produce transcripts (TPM>0):


(2) $$\delta (\theta )=\frac{(1-\rho )(1-\omega_{a})e^{\mu_{i}+\frac{1}{2}\sigma^{2}}}{(1-\rho )(1-\omega_{a})e^{\mu_{i}+\frac{1}{2}\sigma^{2}}+\omega_{a}\sum_{k}\omega_{k}e^{\mu_{a_{k}}+\frac{1}{2}\sigma^{2}}}$$

where $e^{\mu +\frac{1}{2}\sigma^{2}}$ is the mean of a log-normal distribution. Table 4 provides descriptions of the parameter symbols used.

The proportion averaged over the posterior uncertainty of the latent mixture model parameters, θ, is obtained by computing the mean of *δ* from *N* draws of θ from the MCMC:


(3) $$E_{\theta |X}[\delta (\theta )]\approx \frac{1}{N}\sum_{i=1}^{N}\delta (\theta_{i}).$$

*Model Adequacy and Validation.* We used posterior predictive simulation to select a set of hyperparameters and number of mixture components that do not inappropriately influence the posterior. With *zigzag’s* posterior predictive simulation setting we simulated library expression distributions and compared them to the data distributions through plots to select appropriate mixture distributions and prior thresholds (supplementary Data File S1, Supplementary Material online, upper-level plots). We found that 2 active mixture components for the accessory glands and 3 for the testis worked well. We visually analyzed posterior predictive plots output by *zigzag* and found that the simulated library distributions from testes matched very closely with the data, while the accessory glands showed a much weaker match in many of the species (supplementary Data File S1, Supplementary Material online lower-level (library-specific) plots).

We checked the sensitivity of all genes to this mismatch to see which genes may violate the assumption of the same expression state in all replicate samples by performing leave-one-library-out analysis. Each library was removed from the set and expression state probabilities were reestimated. Thus, for a set of 4 replicate libraries, we obtained 4 expression state probabilities for each gene. Any gene where the difference between the maximum and minimum probability of active expression was >0.25 was treated as unknown for downstream analyses. The accessory glands contained many more such genes (58–1049) than the testis (39–116). This could be due to the greater challenge of dissecting the AG cleanly and the presence of contaminants from neighboring tissues, which we expect to be more variable among replicates.

### Orthology Table and Species Tree Inference

An orthology table was estimated using OrthoFinder v. 2.4.0 with default settings (Emms and Kelly 2019). This produced 15,322 gene families including 8,660 conserved single-copy families (i.e. one gene per family in each of the 11 species), constituting 56% of all genes. A comprehensive list of *D. melanogaster* transcription factors was downloaded from FlyMine.org. Of the 757 TFs, 75% were in single-copy gene families.

The phylogenetic tree of the 11 *Drosophila* species was inferred using RevBayes (Höhna et al. 2016). The goal was to create a species tree with accurate relative node ages (branch lengths in time units rather than expected number of nucleotide substitutions) that could then be treated as fixed in the phylogenetic analysis of gene expression states. To construct this tree, we used 12 conserved single-copy genes from the dataset that was used to infer a *Drosophila* chronogram by Turelli et al. (2018). In each species, we used the isoform with the longest protein sequence of the following genes: *Ald1*, *bcd*, *Eno*, *esc*, *Glyp*, *Glys*, *ninaE*, *Pepck1*, *Pgi*, *pic*, *Tpi*, and *Taldo*. Mafft v.7.471 on auto settings was used to align each set of 11 homologous proteins, followed by pal2nal v.14 (Suyama et al. 2006) to convert protein alignments into codon alignments. All codon alignments were then partitioned by codon position for phylogenetic analysis, yielding a dataset with 36 partitions.

All phylogenetic analyses were conducted in RevBayes v. 1.1.0. Posterior samples of relative chronograms were simulated under three clock models: a global clock (one rate for the whole tree), uncorrelated relaxed clock (UCLN) with one rate for each branch which is uncorrelated with neighboring branches, and an autocorrelated relaxed clock model (ACLN) where neighboring branch rates were correlated (Drummond et al. 2006). All partitions were assumed to evolve at the same clock rate on each branch, with root age set to 1. All 3 models assumed the same substitution model for each individual partition (3 partitions per gene for 12 genes): GTR + G + I, with G having 4 rates that are distributed according to a discretized Gamma distribution with mean set to 1 and I being the proportion of invariant sites.

For the tree prior in all analyses, we assumed the sampled birth-death model of Yang and Rannala (1997). For the strict and uncorrelated analyse0s, the number of species in the clade that spans the sampled species was assumed to be around 220–240, which is slightly larger than the number of described species in the *melanogaster* species group (spanning the clade from *D. ananassae* to *D. melanogaster*). We averaged over this uncertainty by specifying an informative prior of Gamma (529, 2.3). MCMCs were run for 75,000 generations with 10% burnin. ESS was checked so that all parameters had ESS>150. Multiple independent MCMCs converged on identical posterior distributions for parameters and trees in each model.

We inferred the topology of the tree under an uncorrelated relaxed clock model which supported the phylogeny used with at least 0.92 posterior probability at each branch except for the placement of *D. kikkawai*, which was equally likely placed with the (*D. anannassae* + *D. bipectinata*) clade as with the other clade at the first post-root split. To estimate branch lengths under a more realistic autocorrelated relaxed clock model, we selected the topology that placed *D. kikkawai* basal to the other 9 species (Suvorov et al. 2021) and treated that as fixed. The tree topology matched almost exactly the topology that was recovered in a larger analysis that included >100 species and several hundred conserved dipteran BUSCO genes (Suvorov et al. 2021). The one difference was the locations of *D. elegans* and *D. ficusphila*, which are reversed in our UCLN analysis with posterior probability = 0.92 on the branch separating the two. The maximum a posteriori (MAP) tree with UCLN MAP estimate of topology and ACLN MAP estimate of branch lengths was used for subsequent analyses of transcriptome evolution.

### Transcriptome Evolution Inference

We modeled the evolution of gene expression states as a continuous-time Markov chain (CTMC) with 2 states, {OFF, ON}. The transition rates between these states vary across genes and branches of the phylogenetic tree. Additionally, the branch-specific rates are correlated between the 2 organs.

#### Relative rates model

We assume that the process is at stationarity over the time scale of the whole tree, with genes at the root being active (ON) in organ *i* with frequency $\pi_{i}$, where *i* indexes the organs {AG, Testis}. The relative transition rate matrix, $Q_{i}$, is parameterized by $\pi_{i}$ such that the mean transition rate across all genes is one (Yang 1994). Specifically,


$$\pi_{i}Q_{i_{21}}+(1-\pi_{i})Q_{i_{12}}=1,$$

where $Q_{i_{12}}$ is proportional to the rate at which genes are activated (turned ON), and $Q_{i_{21}}$ is proportional to the rate at which genes are deactivated (turned OFF). The relative rate matrix is therefore:


$$Q_{i}=\left[\begin{array}{cc} - & \frac{1}{2(1-\pi_{i})}\\ \frac{1}{2\pi_{i}} & - \end{array}\right],$$

where dashes along the diagonal are set so that rows sum to zero.

#### Among-gene rate variation model

To model rate variability among genes, we specified a scale factor, $a_{i,g}$, to scale the relative rate matrix, $Q_{i}$ for each gene, *g*. This variable is drawn from a discretized gamma distribution with mean fixed to 1 (Yang 1994):


$$a_{i,g}∼\text{DiscretizedGamma}(\alpha =\xi_{i},\beta =\xi_{i},k=6).$$

Here, the distribution is a mixture model in which a gamma distribution, with mean α/β=1 and variance $\alpha /\beta^{2}=1/\xi_{i}$, and is divided into k=6 intervals of equal probability mass. The mixture weights are thus 1/6. The rate category at each interval is the mean rate for that interval. $\xi_{i}$ is the precision of the distribution.

#### Among-branch rate heterogeneity model

In our model of turnover rate heterogeneity across branches of the tree, the evolutionary rates for both organs on each branch follow a bivariate log-normal distribution. The distribution of the bivariate log-transformed rates, $\log\;r_{b}$, for the 2 organs on branch *b* are given by:


$$\log\;r_{b}∼\text{MVN}(\mu ,\Sigma ),$$

The transition rate matrix for gene, *g*, in organ, *i*, on branch, *b*, is thus equal to


$$r_{i,b}a_{i,g}Q_{i}.$$

We summarize the prior distributions for the paramaters of the above model in Table 5:

**Table 5.** Summary of the probabilistic model parameters and their distributions or definitions

Parameter	Distribution Type	Distribution Parameters	Description
$\pi_{i}$	Beta	α=β=1	Frequency genes at the root are active
$\xi_{i}$	LogNormal	logmean = ln(5),	Precision of the discretized gamma
		logsd = 0.587	distribution of $a_{i,g}$
*ϕ*	Uniform	min=−10,	Log mean branch scale parameter
		max=1	
$\alpha_{i}$	Beta	α=2,β=2	Proportional mean rates of 2 organs
$\mu_{i}$	Defined	$\phi +\ln\;(2\alpha_{i})$	Log mean branch rate of organ *i*
*θ*	LogUniform	min=0.0001,	Branch rate variance scale parameter
		max=5	
$\beta_{i}$	Beta	α=10,β=10	Proportional branch rate variance
$\sigma_{i}^{2}$	Defined	$\theta +2\beta_{i}$	Variance of branch rates of organ *i*
*C*	LKJ	η=1	Correlation matrix
*S*	Defined	$\left(\begin{array}{cc} \sigma_{1} & 0\\ 0 & \sigma_{2} \end{array}\right)$	Organ branch rate standard deviation
*Σ*	Defined	*SCS*	Covariance matrix

The LKJ distribution is the Lewandowski-Kurowicka-Joe distribution (Lewandowski et al. 2009).

#### Markov Chain Monte Carlo (MCMC)

We sampled from the model posterior using RevBayes (Hohna et al. 2016). After an initial burnin period, we ran the MCMC for 10,000 generations sampling every 5 generations. We confirmed that two independent runs converged by measuring the PSRF for all parameters and checking that it was < 1.2. We also checked that ESS was at least 200.

### GO Analysis

GO analyses were performed in R v4.1.1 using clusterProfiler (v4.0.5; Yu et al. 2012). Outputs were plotted using clusterProfiler, ggplot2 (v3.4.4), and ggVennDiagram (v1.5.2; Gao et al. 2021 ). Ontogeny was limited to “Biological Process” and a Benjamini-Hochberg procedure was used to correct for the false discovery rate. A *q*-value cutoff of 0.05 was used. The org.Dm.eg.db (v3.13.0) package was used for gene annotation data.

### BEST Analysis

The prior distributions and data distribution assumed in *BEST* (Kruschke 2013) are listed in Table 6.

**Table 6.** Summary of the probabilistic model parameters and distributions with specified priors

Parameter	Distribution Type	Distribution Parameters	Description
$\mu_{1}$	Normal	$\mu =\text{mean}(\log\;y_{1}),$ $\sigma =\text{sd}(\log\;y_{1})\times 1,000$	Mean of group 1
$\mu_{2}$	Normal	$\mu =\text{mean}(\log\;y_{2}),$ $\sigma =\text{sd}(\log\;y_{2})\times 1,000$	Mean of group 2
$\sigma_{1}$	Uniform	$\min=\text{sd}(\log\;y_{1})/1,000,$ $\max=\text{sd}(\log\;y_{1})\times 1,000$	Standard deviation of group 1
$\sigma_{2}$	Uniform	$\min=\text{sd}(\log\;y_{2})/1,000,$ $\max=\text{sd}(\log\;y_{2})\times 1,000$	Standard deviation of group 2
*ν* - 1	Exponential	rate=1/29	Degrees of freedom for the
			t-distribution (shifted by 1)
$y_{1}$	t-distribution	$\mu =\mu_{1},\sigma =\sigma_{1},\nu =\nu$	Observed data for group 1
$y_{2}$	t-distribution	$\mu =\mu_{2},\sigma =\sigma_{2},\nu =\nu$	Observed data for group 2

## Supplementary Material

msaf106_Supplementary_Data

## Data Availability

The processed data, pipeline scripts and final analysis scripts underlying results for this article are available through our GitHub repository at https://github.com/ammonthompson/transcriptome_turnover. Raw reads data are deposited in Genbank Gene Expression Omnibus with accession GSE274048 (https://www.ncbi.nlm.nih.gov/geo/query/acc.cgi?acc=GSE274048).
