# Supplementary material for: Quantifying Transcriptome Turnover on Phylogenies by Modeling Gene Expression as a Binary Trait
Source: Mol Biol Evol. 2025 May 27;42(5):msaf106. doi: 10.1093/molbev/msaf106 (PMC12108096; doi:10.1093/molbev/msaf106)
Supplement: msaf106_Supplementary_Data [file msaf106_supplementary_data.zip › Data_file_S1_postpred_mixture_distro_all.pdf]

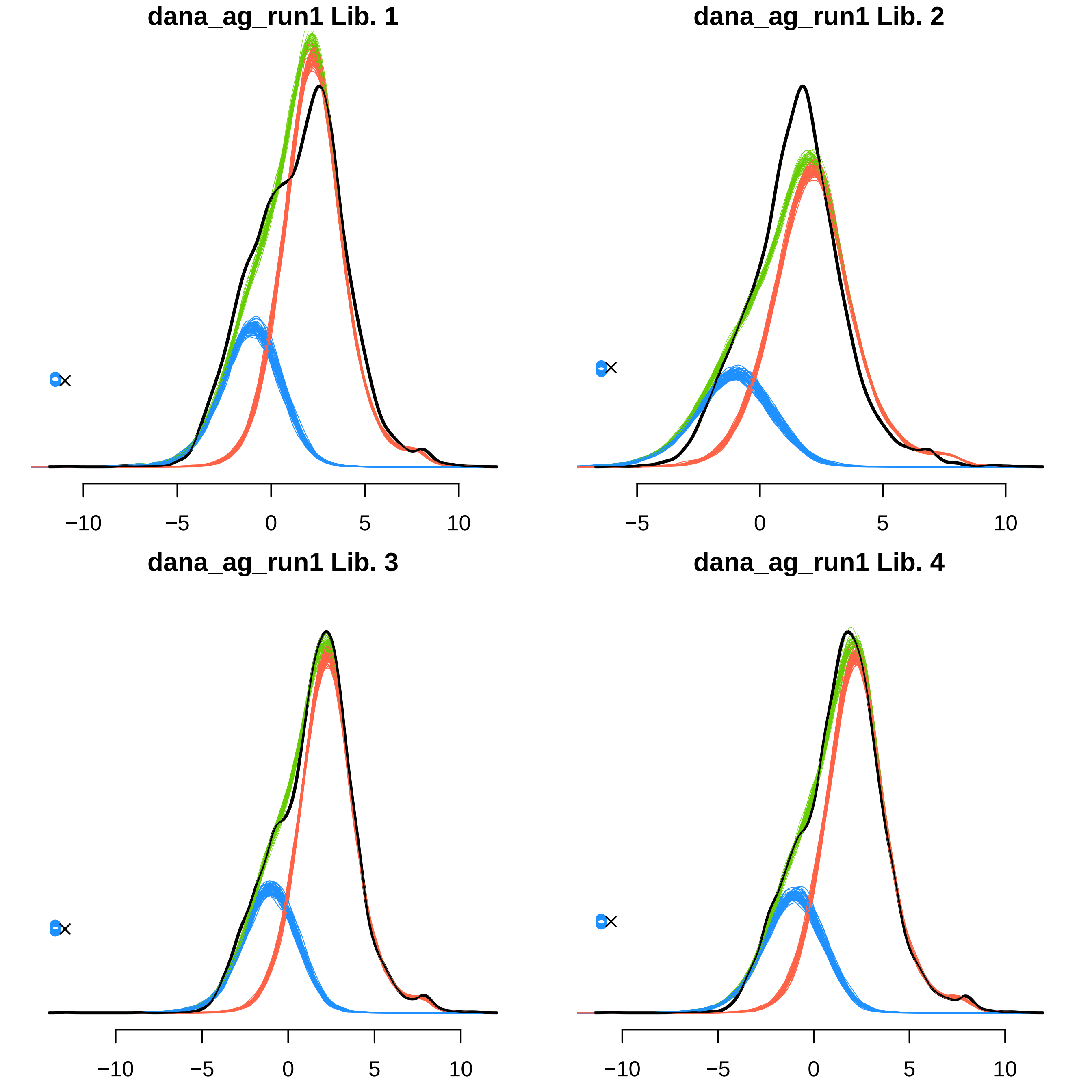
dana_ag_run1 Lib. 1
−10
−5
0
5
10
dana_ag_run1 Lib. 2
−5
0
5
10
dana_ag_run1 Lib. 3
−10
−5
0
5
10
dana_ag_run1 Lib. 4
−10
−5
0
5
10

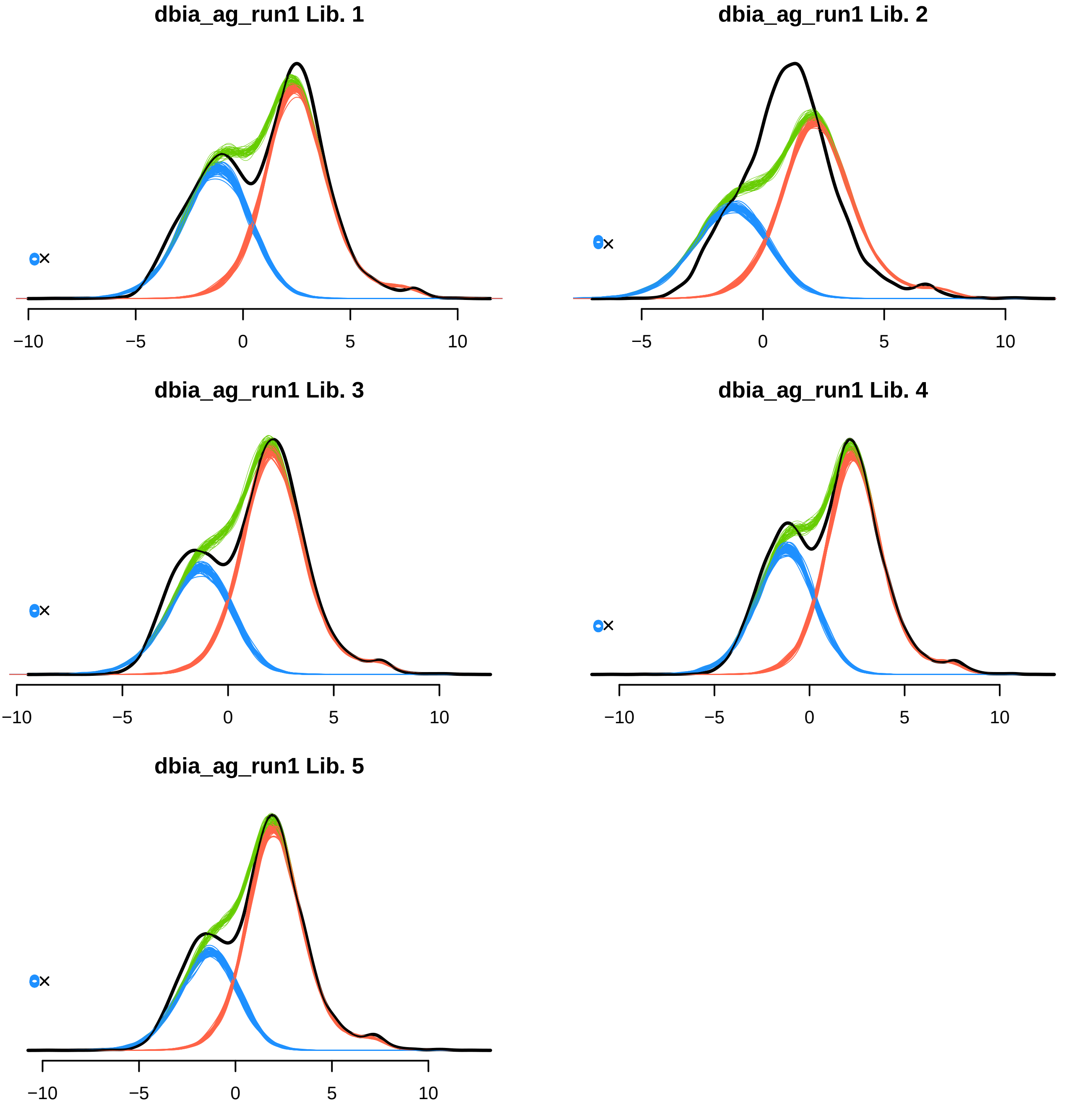
dbia_ag_run1 Lib. 1
−10
−5
0
5
10
dbia_ag_run1 Lib. 2
−5
0
5
10
dbia_ag_run1 Lib. 3
−10
−5
0
5
10
dbia_ag_run1 Lib. 4
−10
−5
0
5
10
dbia_ag_run1 Lib. 5
−10
−5
0
5
10

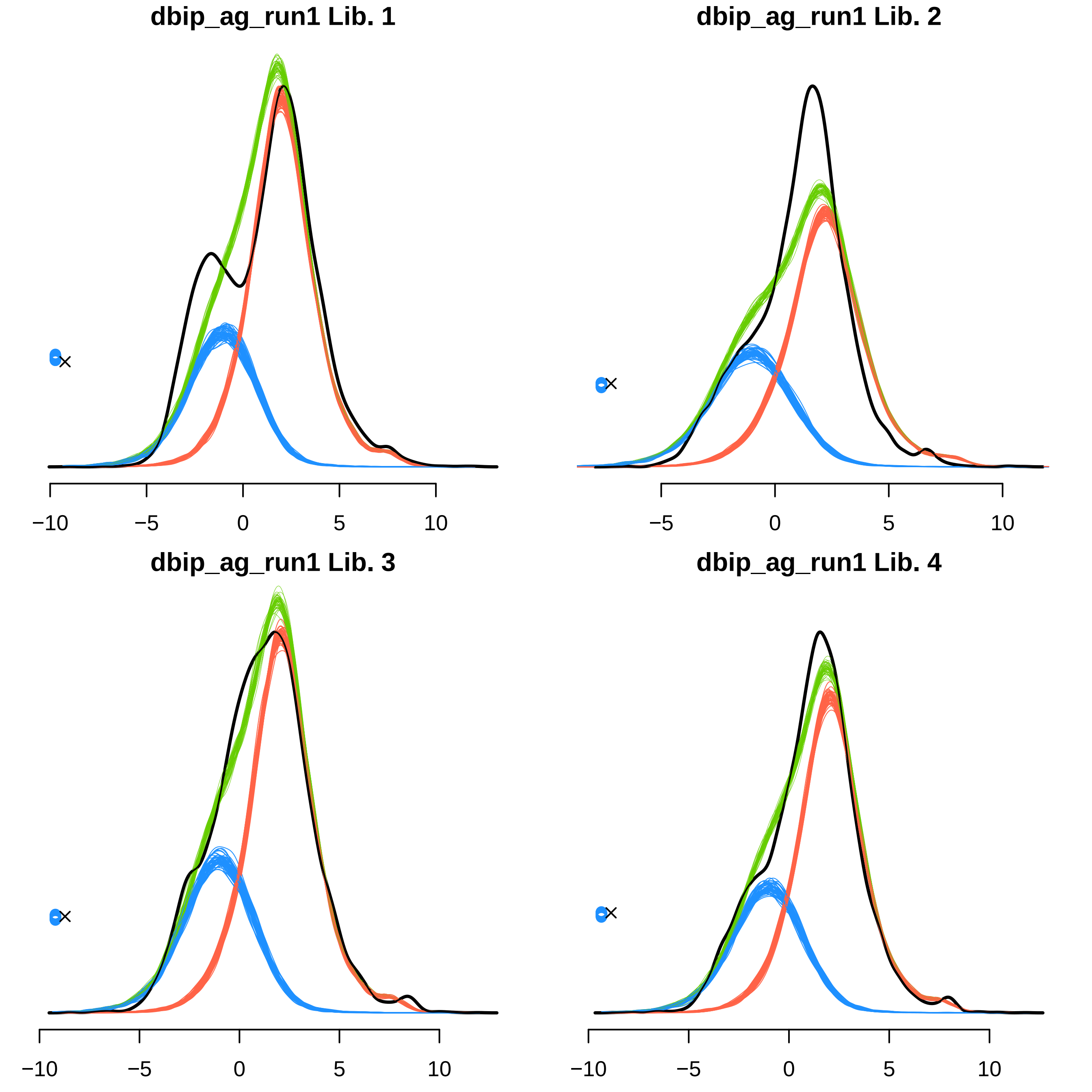

dbip_ag_run1 Lib. 1
−10
−5
0
5
10
dbip_ag_run1 Lib. 2
−5
0
5
10
dbip_ag_run1 Lib. 3
−10
−5
0
5
10
dbip_ag_run1 Lib. 4
−10
−5
0
5
10

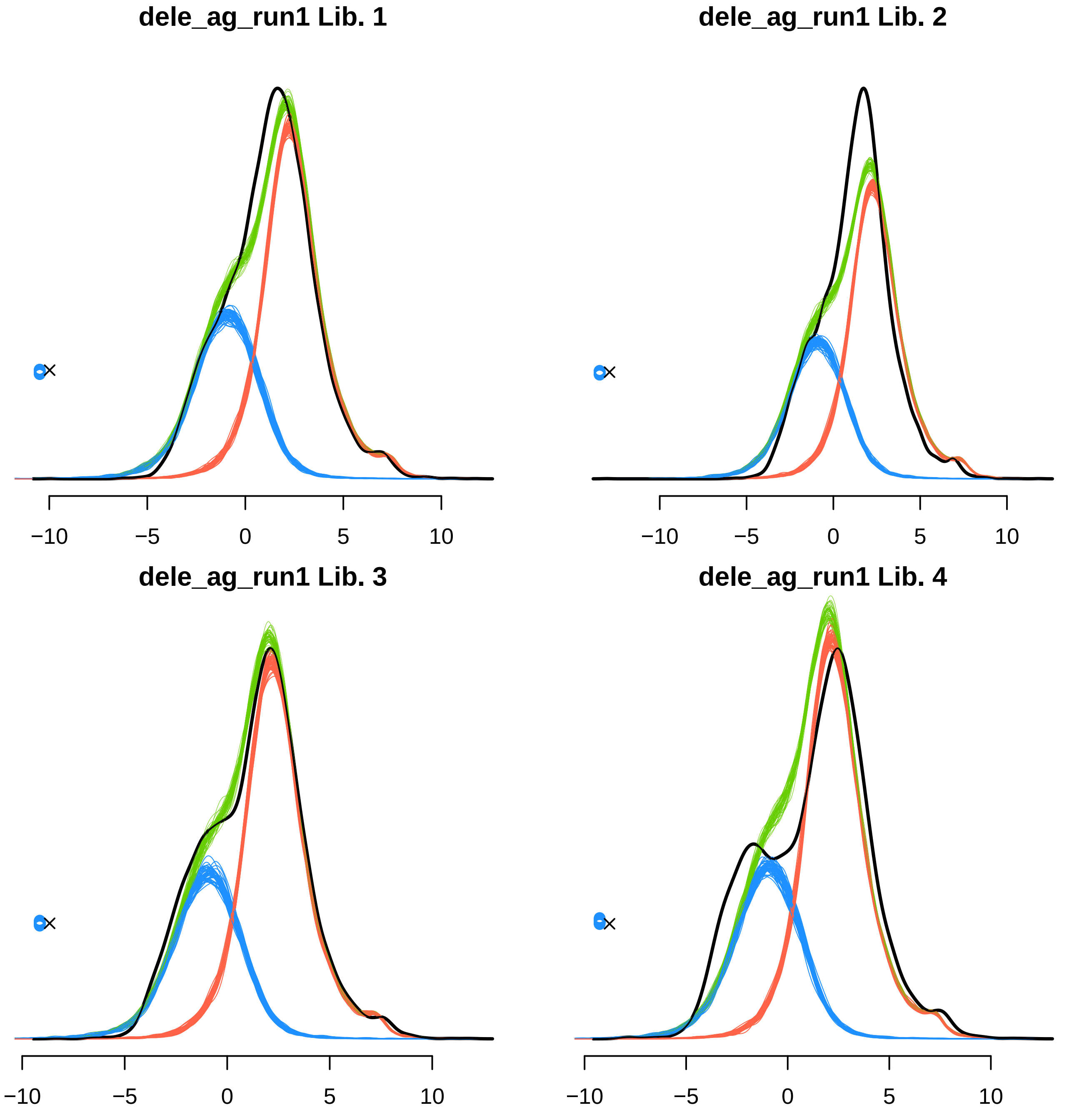

dele_ag_run1 Lib. 1
−10
−5
0
5
10
dele_ag_run1 Lib. 2
−10
−5
0
5
10
dele_ag_run1 Lib. 3
−10
−5
0
5
10
dele_ag_run1 Lib. 4
−10
−5
0
5
10

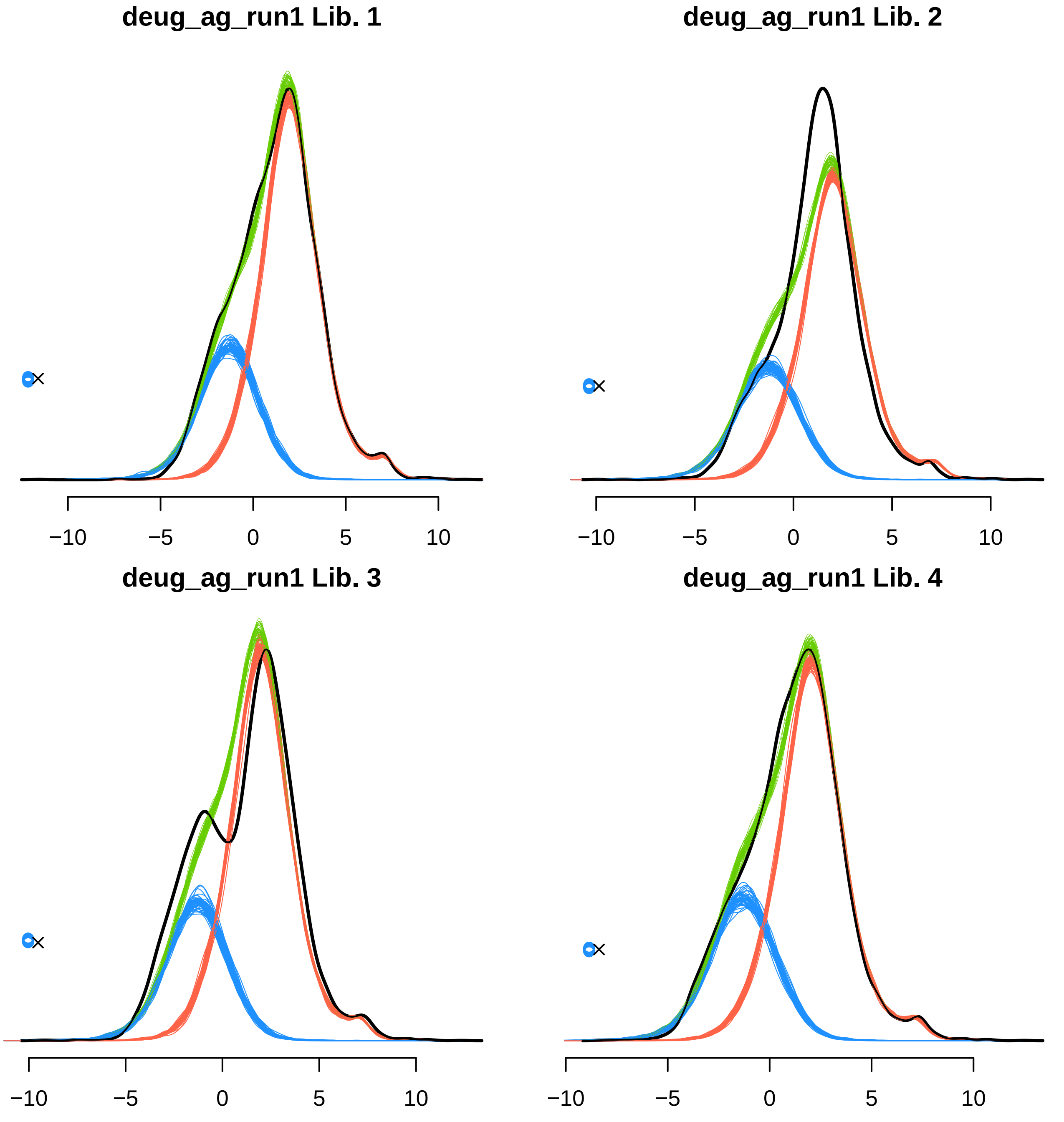

deug_ag_run1 Lib. 1
−10
−5
0
5
10
deug_ag_run1 Lib. 2
−10
−5
0
5
10
deug_ag_run1 Lib. 3
−10
−5
0
5
10
deug_ag_run1 Lib. 4
−10
−5
0
5
10

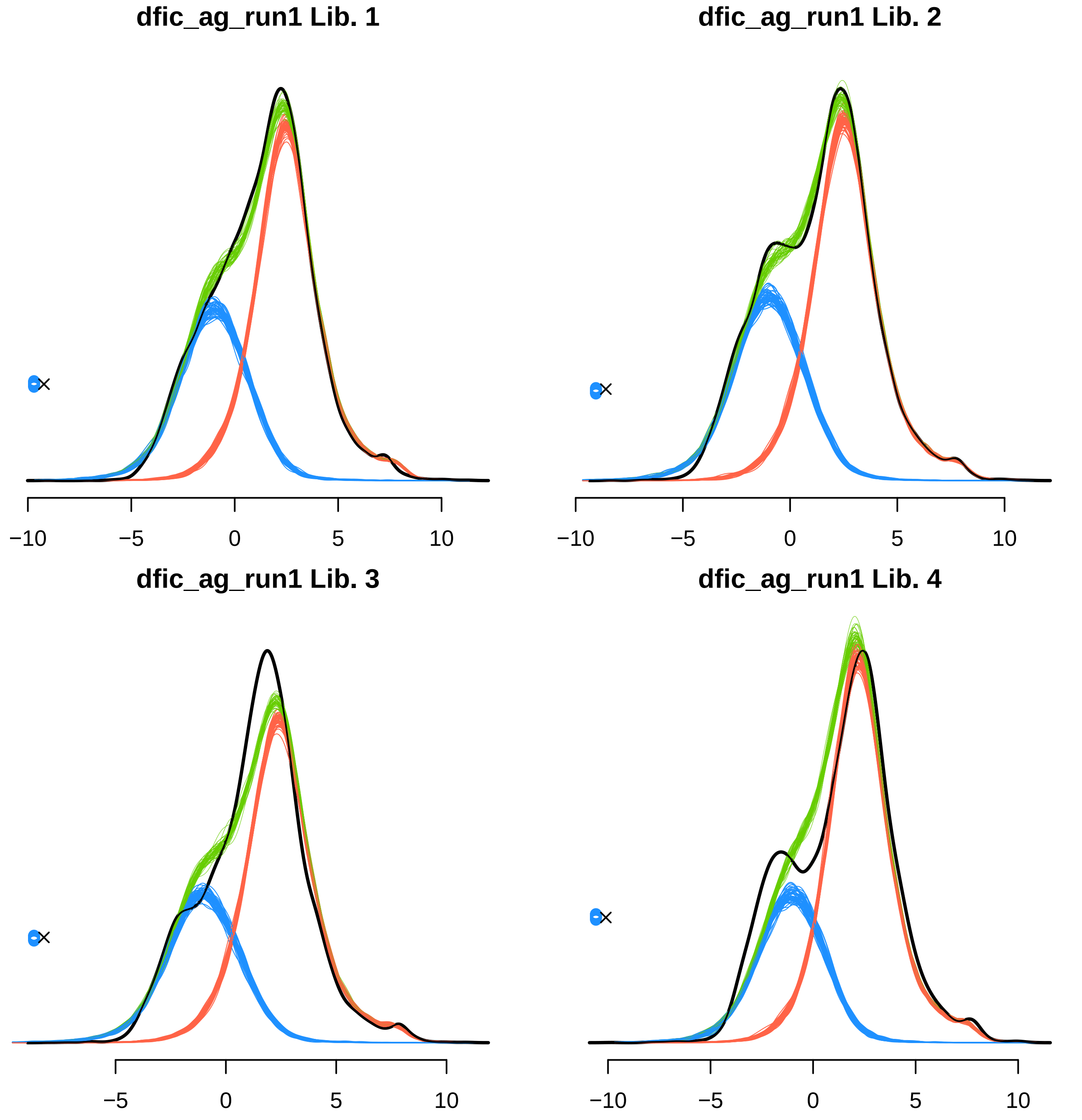
dfic_ag_run1 Lib. 1
−10
−5
0
5
10
dfic_ag_run1 Lib. 2
−10
−5
0
5
10
dfic_ag_run1 Lib. 3
−5
0
5
10
dfic_ag_run1 Lib. 4
−10
−5
0
5
10

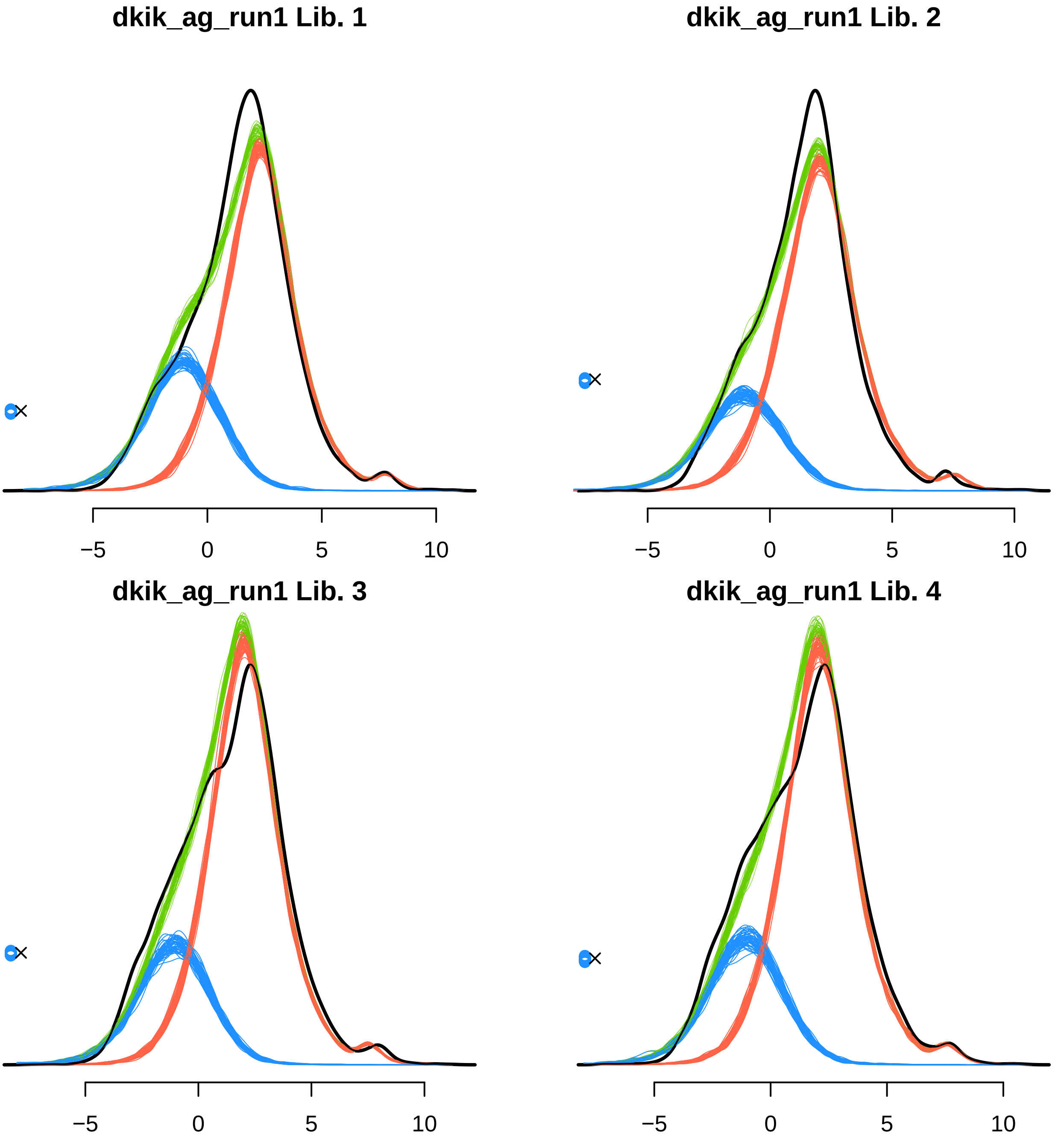
dkik_ag_run1 Lib. 1
−5
0
5
10
dkik_ag_run1 Lib. 2
−5
0
5
10
dkik_ag_run1 Lib. 3
−5
0
5
10
dkik_ag_run1 Lib. 4
−5
0
5
10

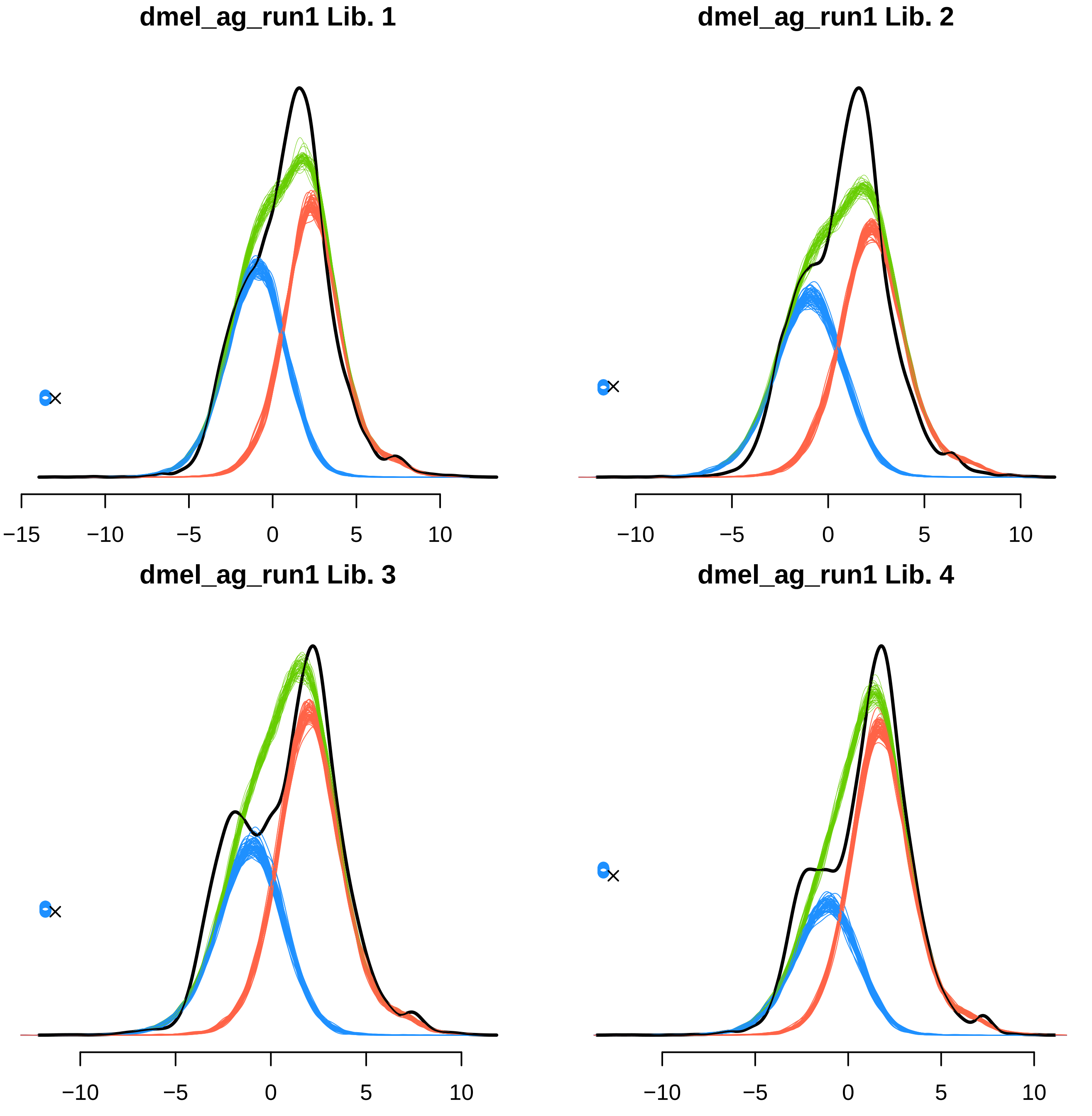
dmel_ag_run1 Lib. 1
−15
−10
−5
0
5
10
dmel_ag_run1 Lib. 2
−10
−5
0
5
10
dmel_ag_run1 Lib. 3
−10
−5
0
5
10
dmel_ag_run1 Lib. 4
−10
−5
0
5
10

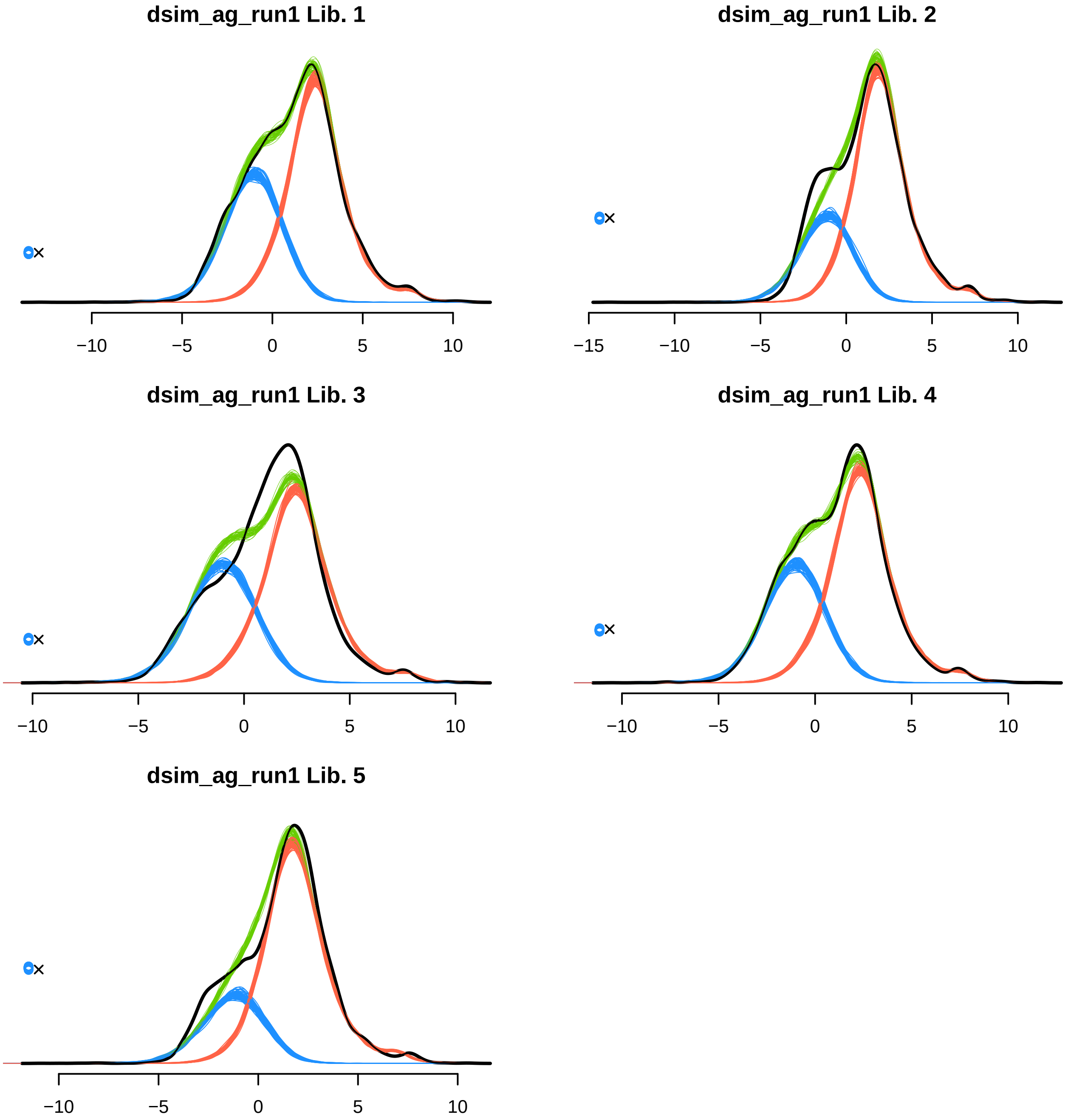
dsim_ag_run1 Lib. 1
−10 −5 0 5 10
dsim_ag_run1 Lib. 2
−15 −10 −5 0 5 10
dsim_ag_run1 Lib. 3
−10 −5 0 5 10
dsim_ag_run1 Lib. 4
−10 −5 0 5 10
dsim_ag_run1 Lib. 5
−10 −5 0 5 10

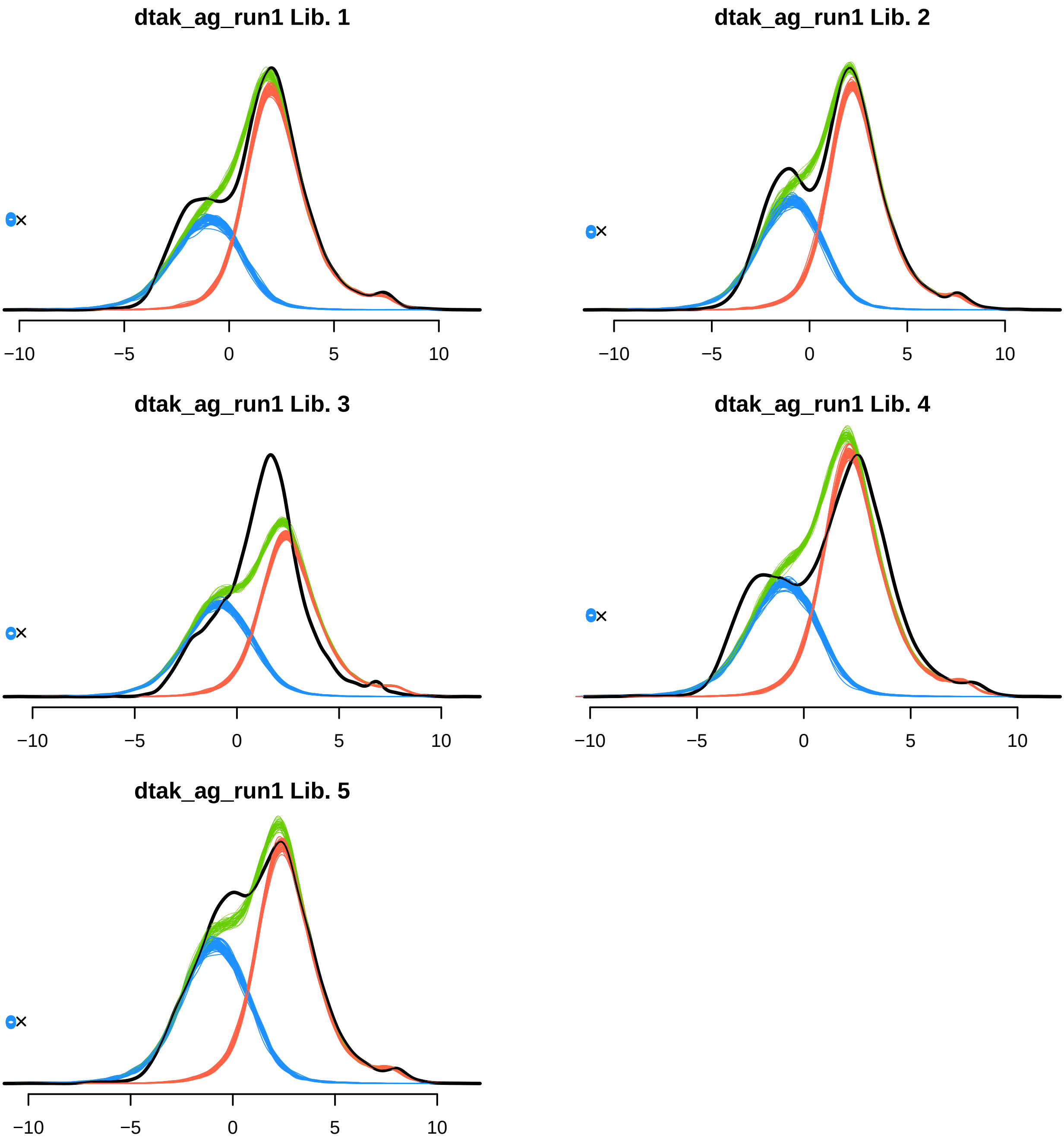
dtak_ag_run1 Lib. 1
dtak_ag_run1 Lib. 2
dtak_ag_run1 Lib. 3
dtak_ag_run1 Lib. 4
dtak_ag_run1 Lib. 5
−10
−5
0
5
10

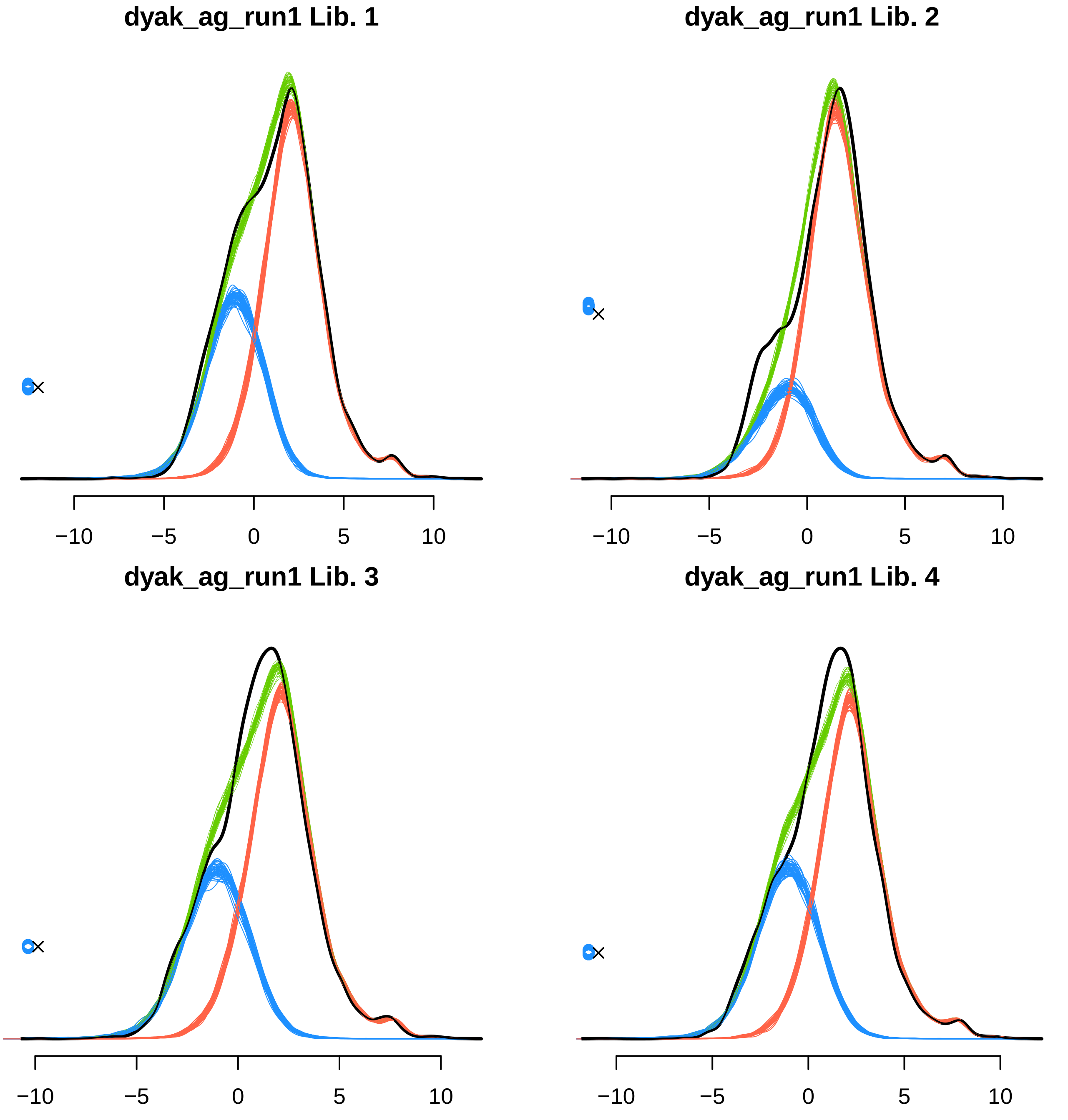

dyak_ag_run1 Lib. 1
dyak_ag_run1 Lib. 2
dyak_ag_run1 Lib. 3
dyak_ag_run1 Lib. 4
−10
−5
0
5
10

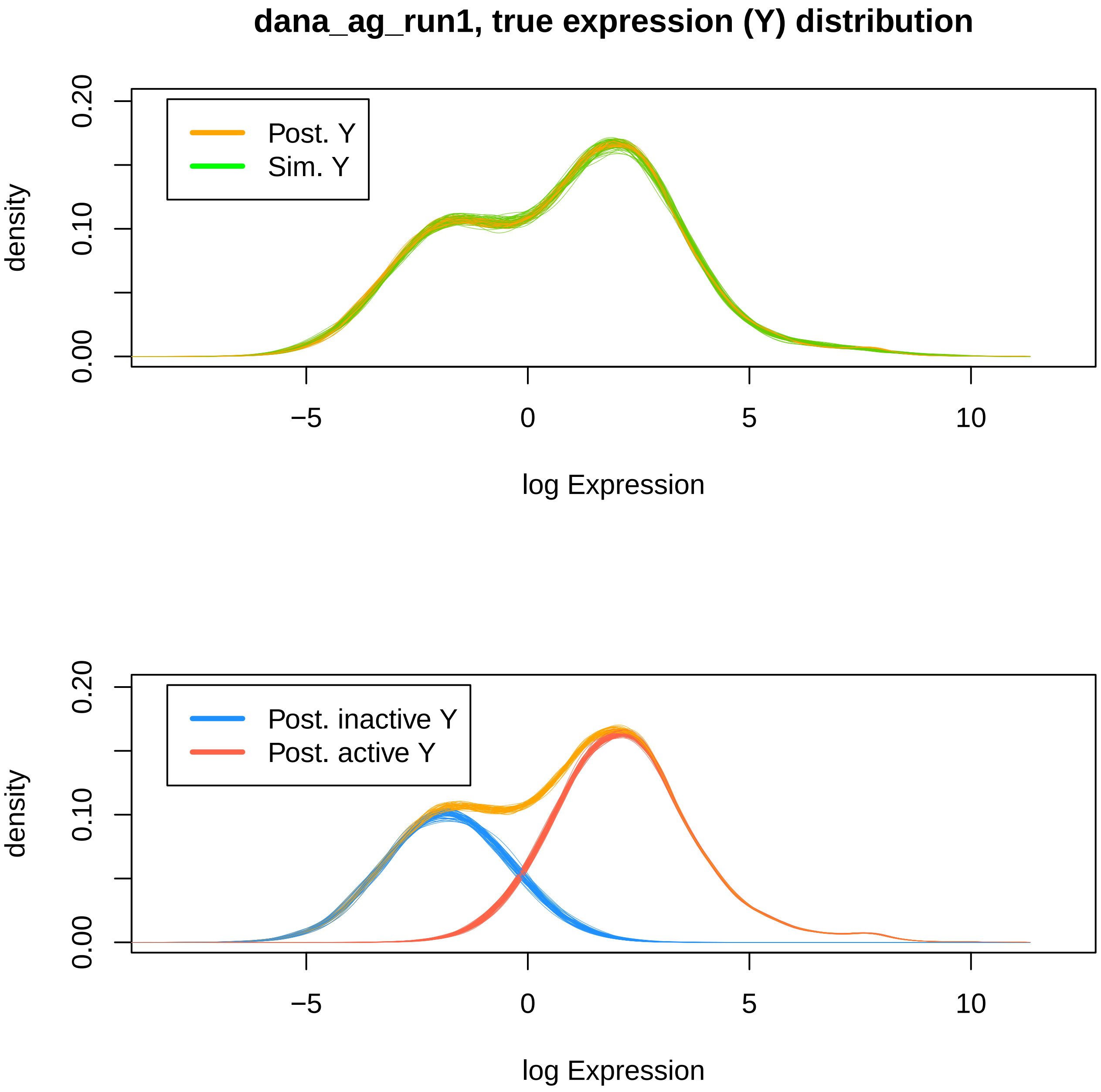
dana_ag_run1, true expression (Y) distribution
Post. Y
Sim. Y
density
0.00
0.10
0.20
−5
0
5
10
log Expression
Post. inactive Y
Post. active Y
density
0.00
0.10
0.20
−5
0
5
10
log Expression

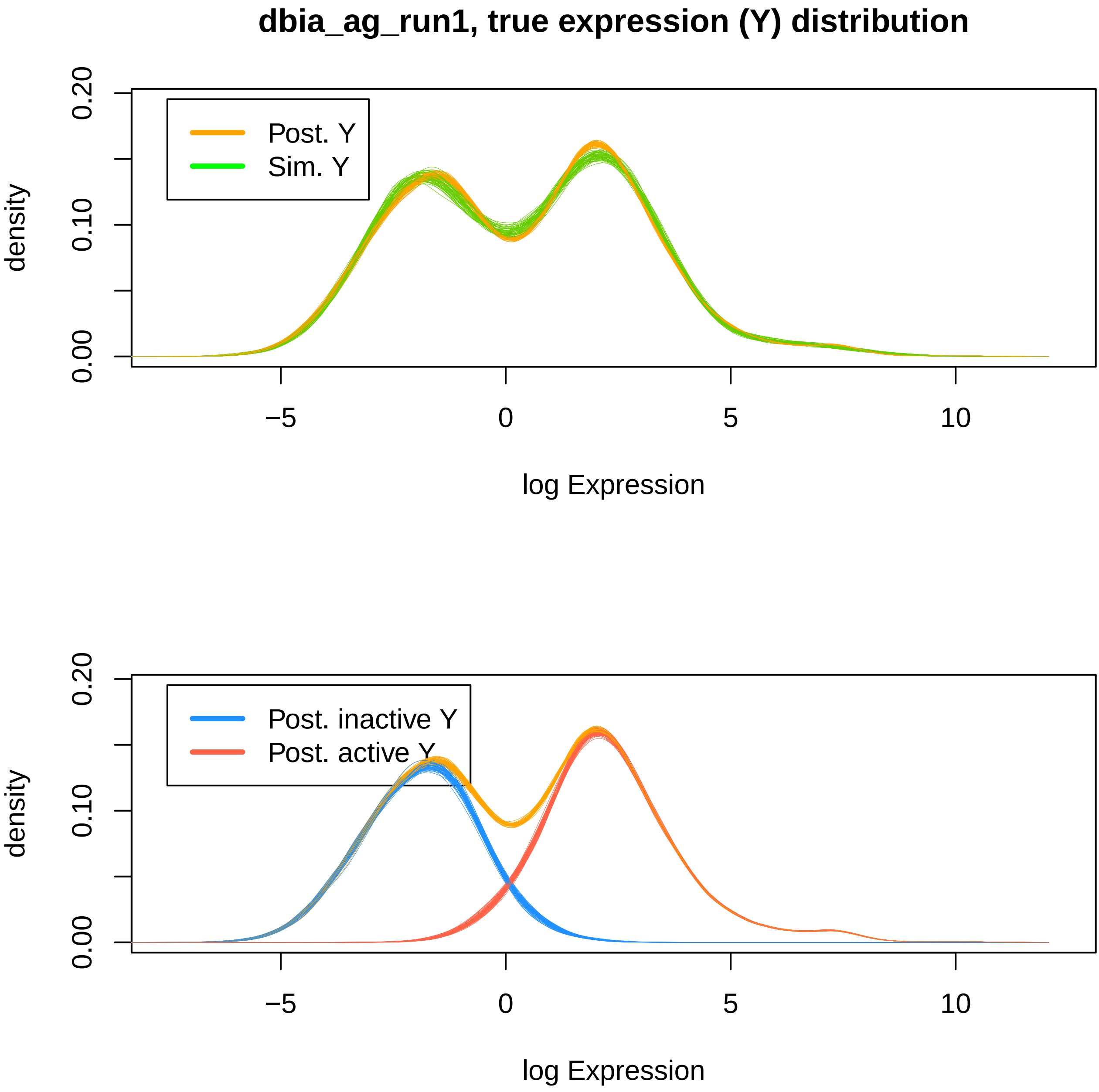
dbia_ag_run1, true expression (Y) distribution
Post. Y
Sim. Y
density
0.00
0.10
0.20
−5
0
5
10
log Expression
Post. inactive Y
Post. active Y
density
0.00
0.10
0.20
−5
0
5
10
log Expression

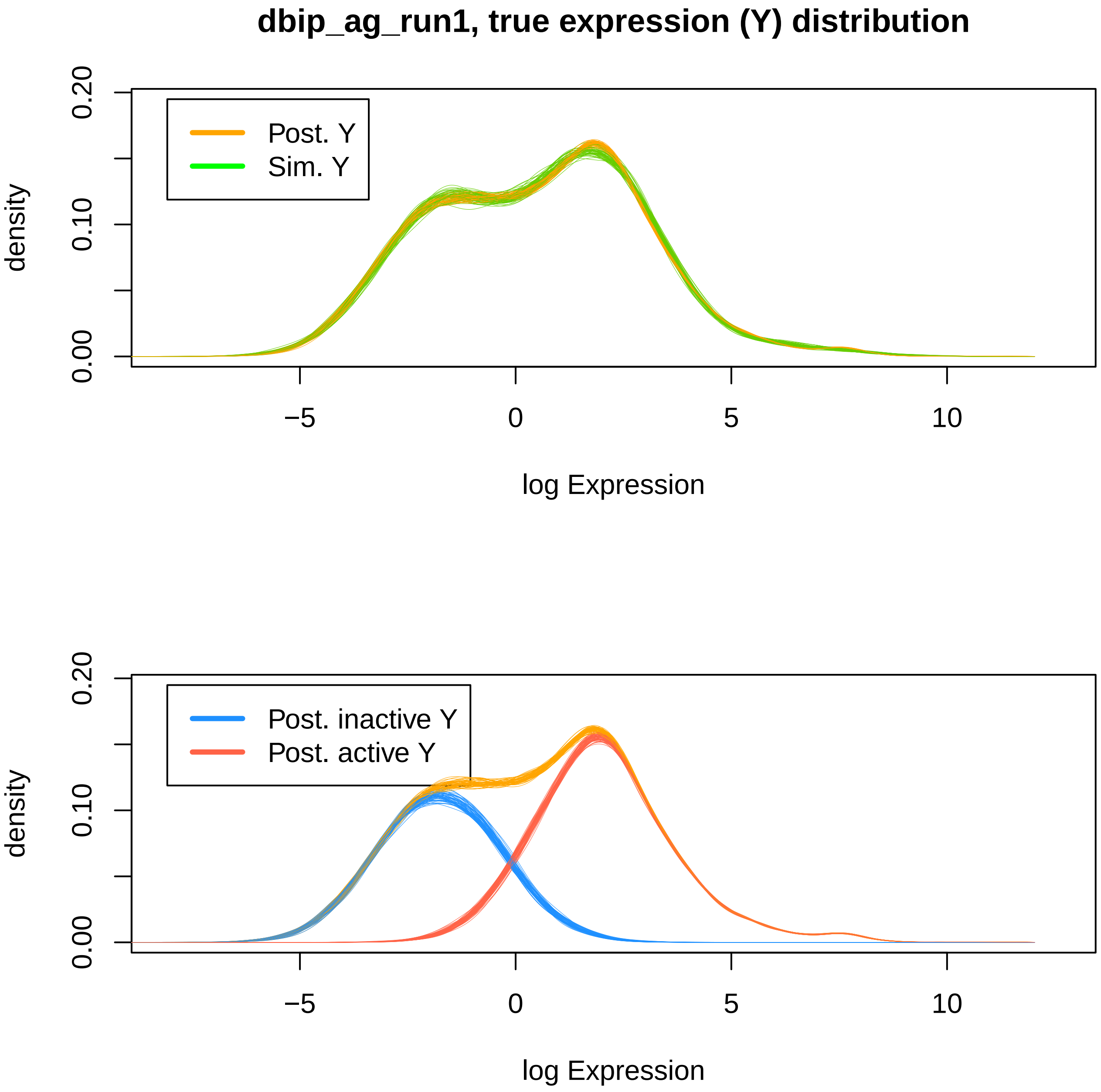

dbip_ag_run1, true expression (Y) distribution
Post. Y
Sim. Y
density
0.00
0.10
0.20
−5
0
5
10
log Expression
Post. inactive Y
Post. active Y
density
0.00
0.10
0.20
−5
0
5
10
log Expression

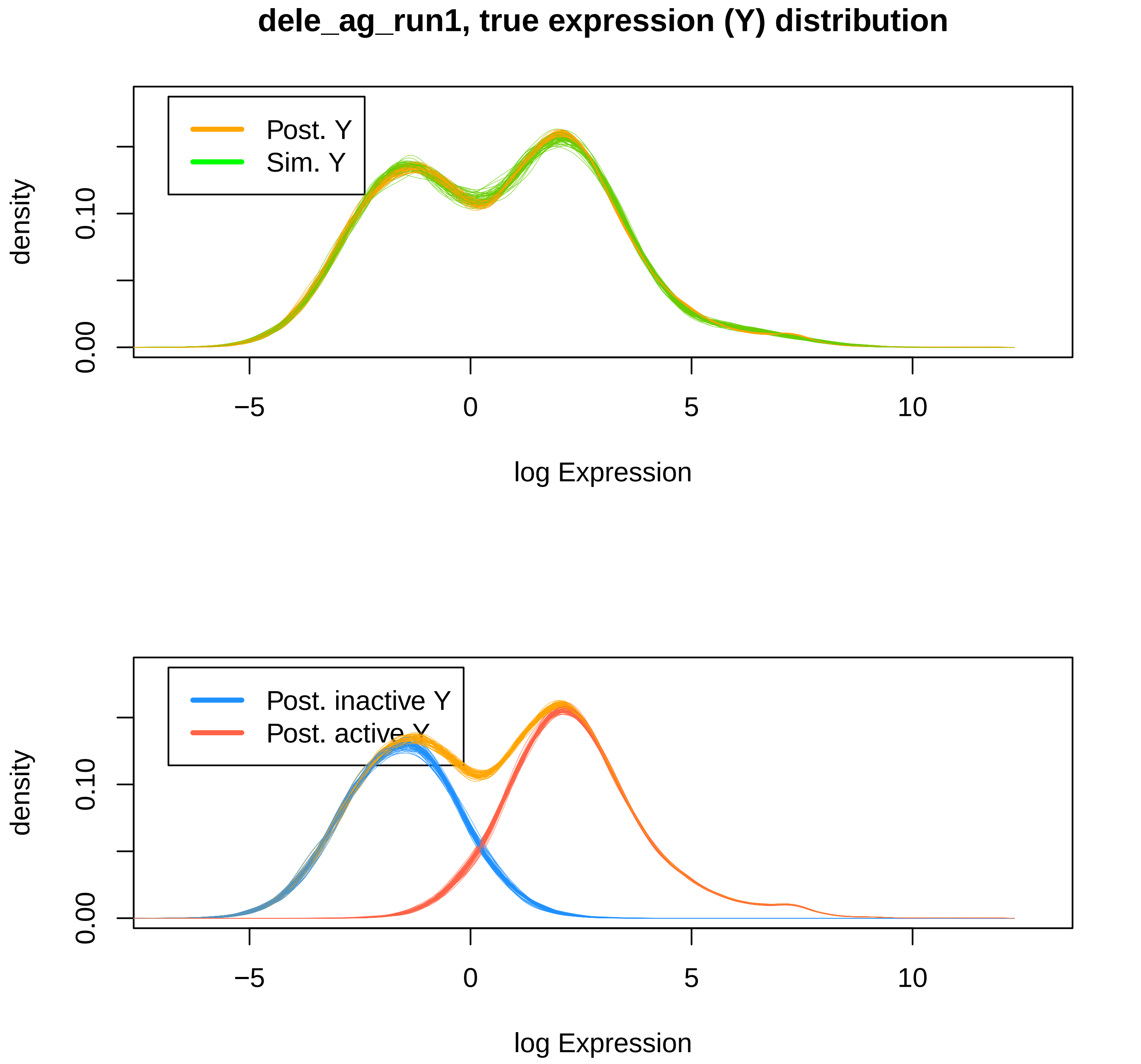

dele_ag_run1, true expression (Y) distribution
Post. Y
Sim. Y
density
0.00
0.10
−5
0
5
10
log Expression
Post. inactive Y
Post. active Y
density
0.00
0.10
−5
0
5
10
log Expression

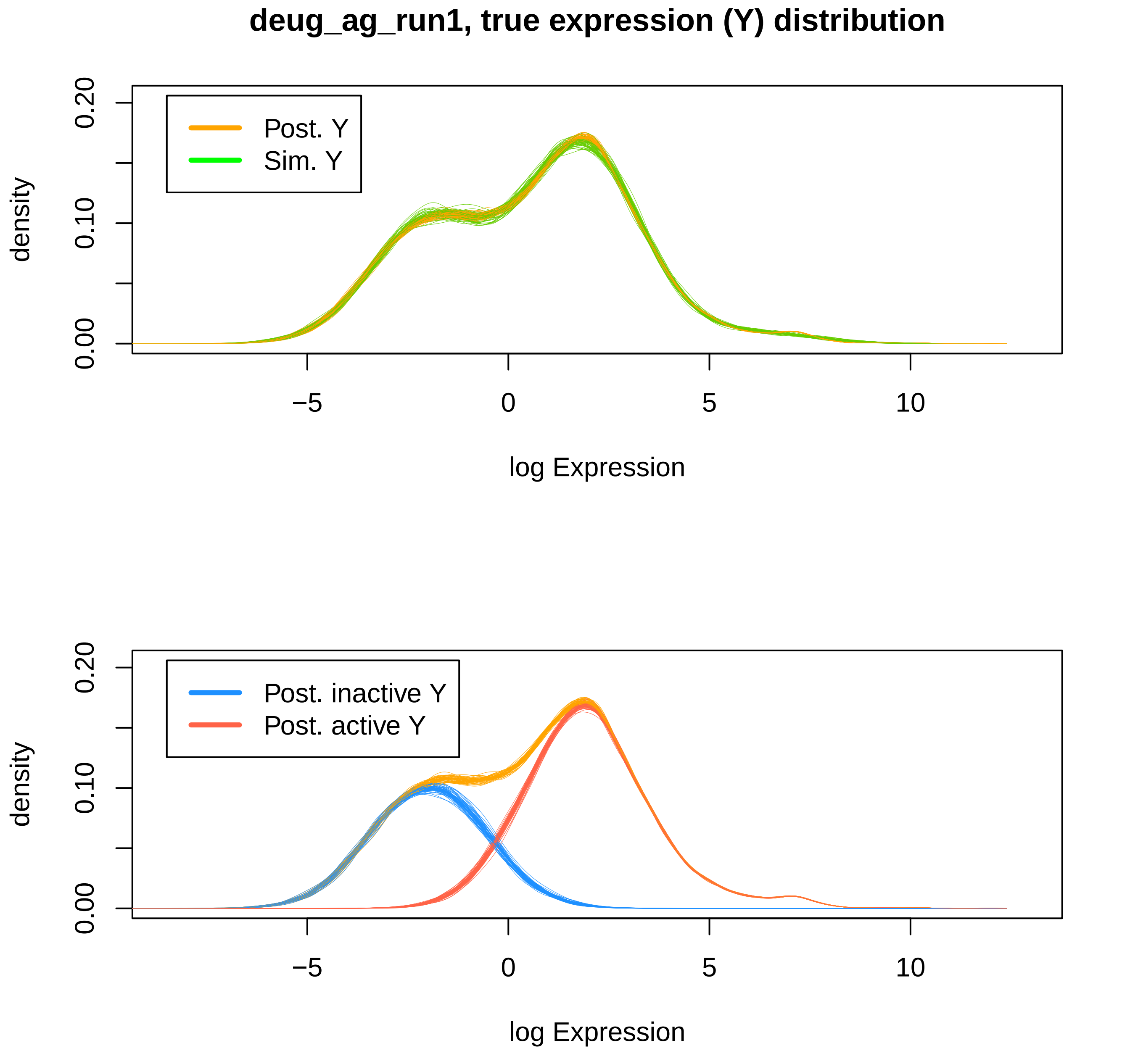

deug_ag_run1, true expression (Y) distribution
Post. Y
Sim. Y
density
0.00
0.10
0.20
−5
0
5
10
log Expression
Post. inactive Y
Post. active Y
density
0.00
0.10
0.20
−5
0
5
10
log Expression

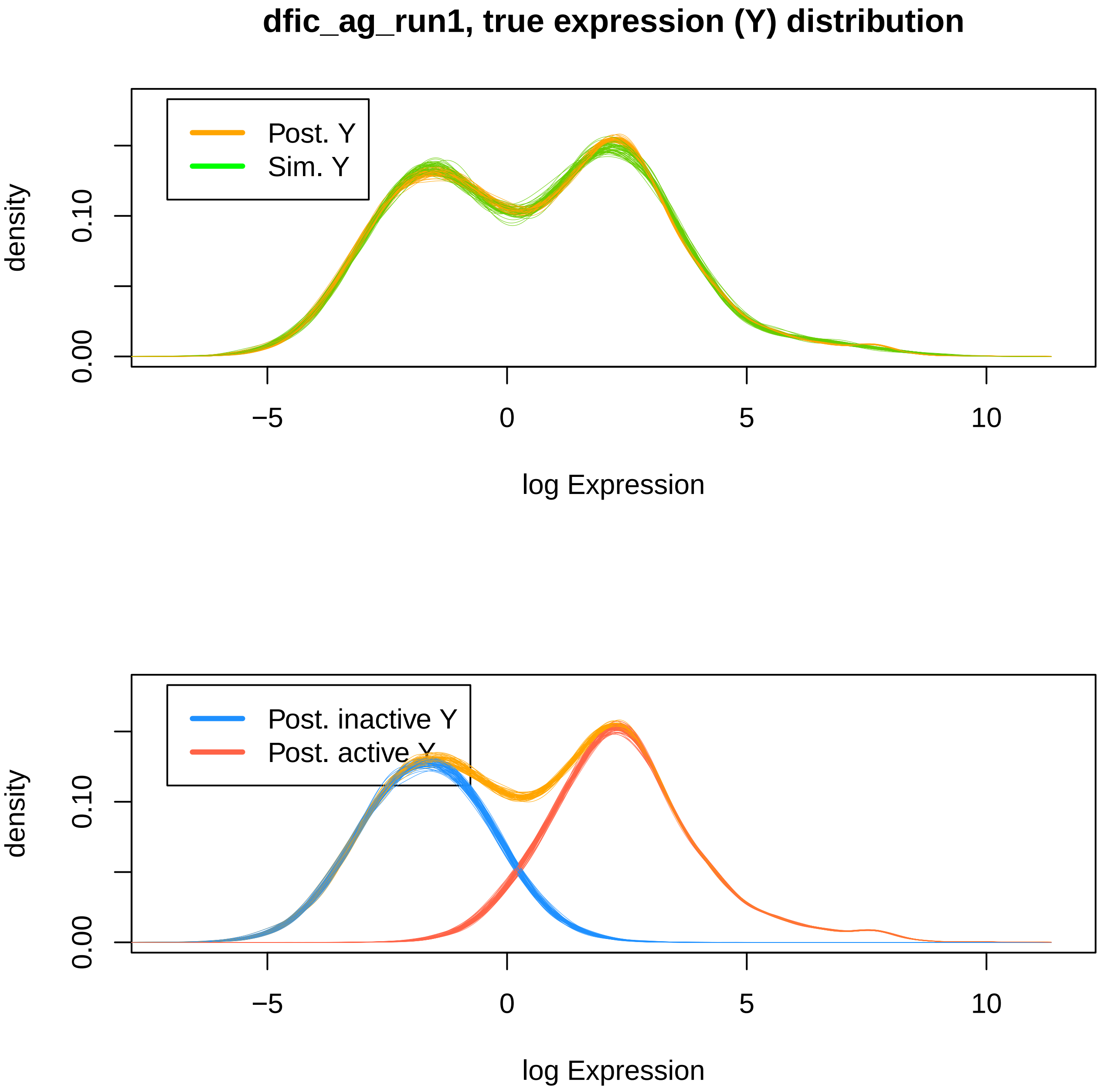

dfic_ag_run1, true expression (Y) distribution
Post. Y
Sim. Y
density
0.00
0.10
−5
0
5
10
log Expression
Post. inactive Y
Post. active Y
density
0.00
0.10
−5
0
5
10
log Expression

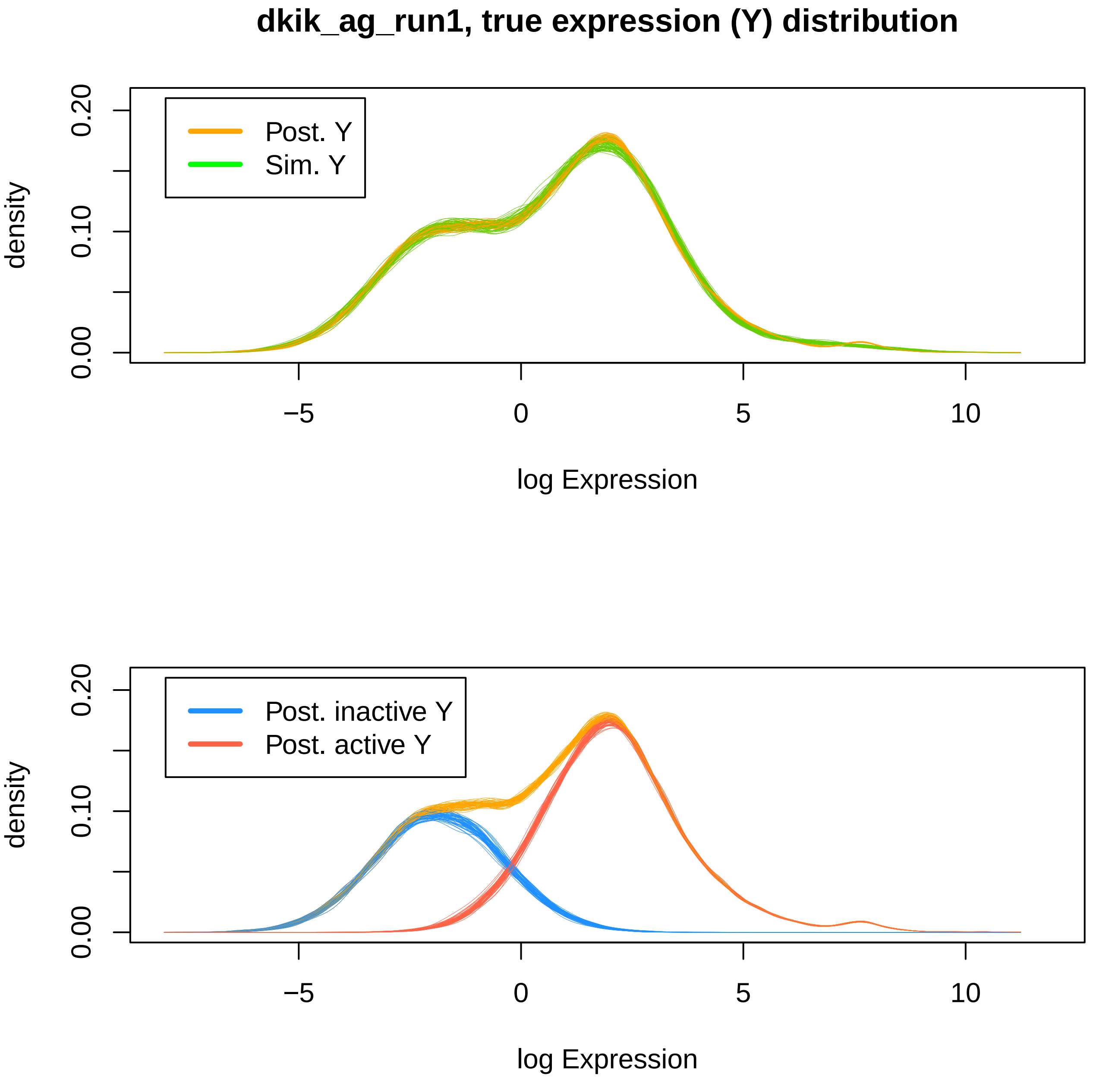

dkik_ag_run1, true expression (Y) distribution
Post. Y
Sim. Y
density
0.00
0.10
0.20
−5
0
5
10
log Expression
Post. inactive Y
Post. active Y
density
0.00
0.10
0.20
−5
0
5
10
log Expression

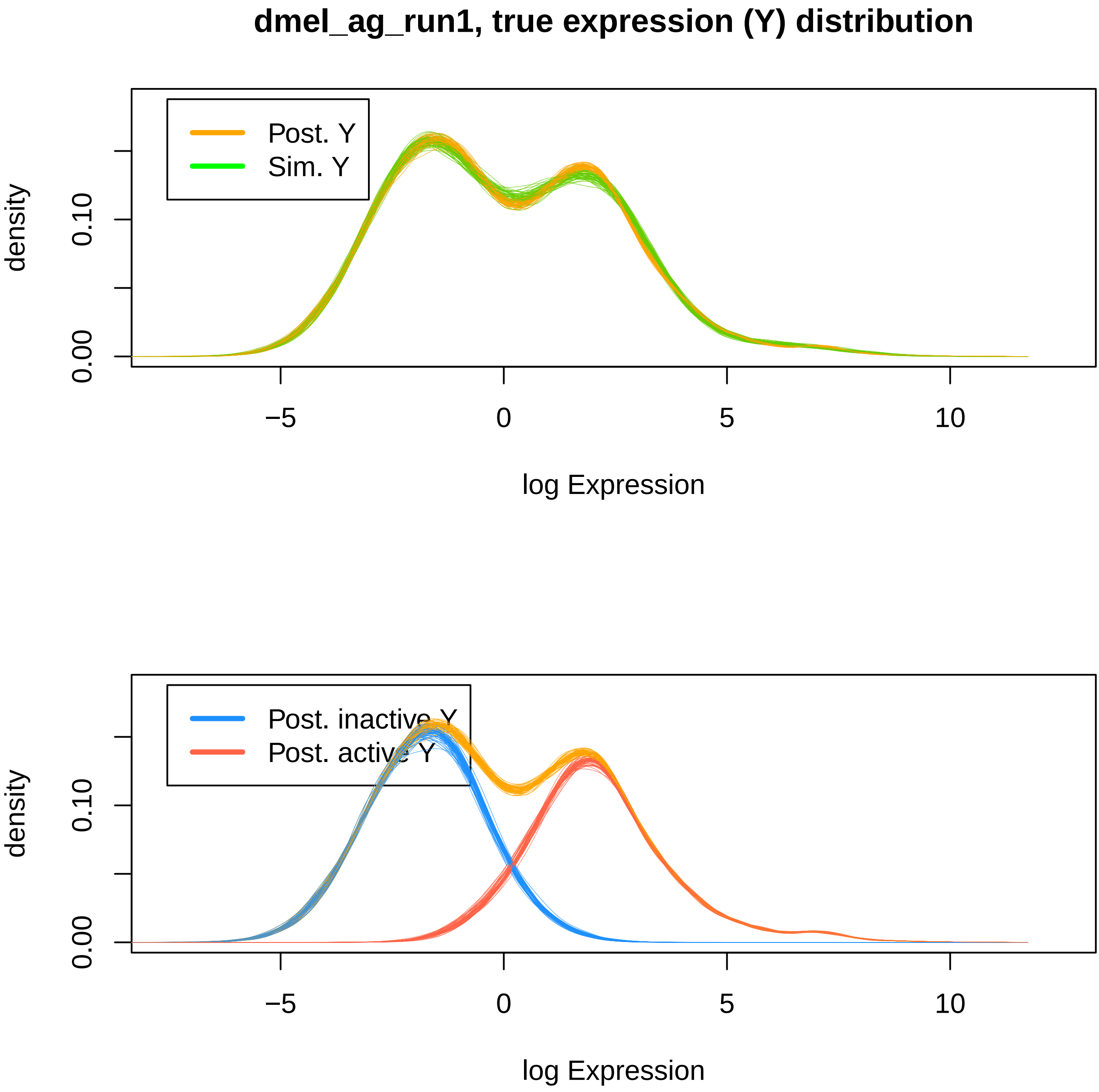

dmel_ag_run1, true expression (Y) distribution
Post. Y
Sim. Y
density
0.00
0.10
−5
0
5
10
log Expression
Post. inactive Y
Post. active Y
density
0.00
0.10
−5
0
5
10
log Expression

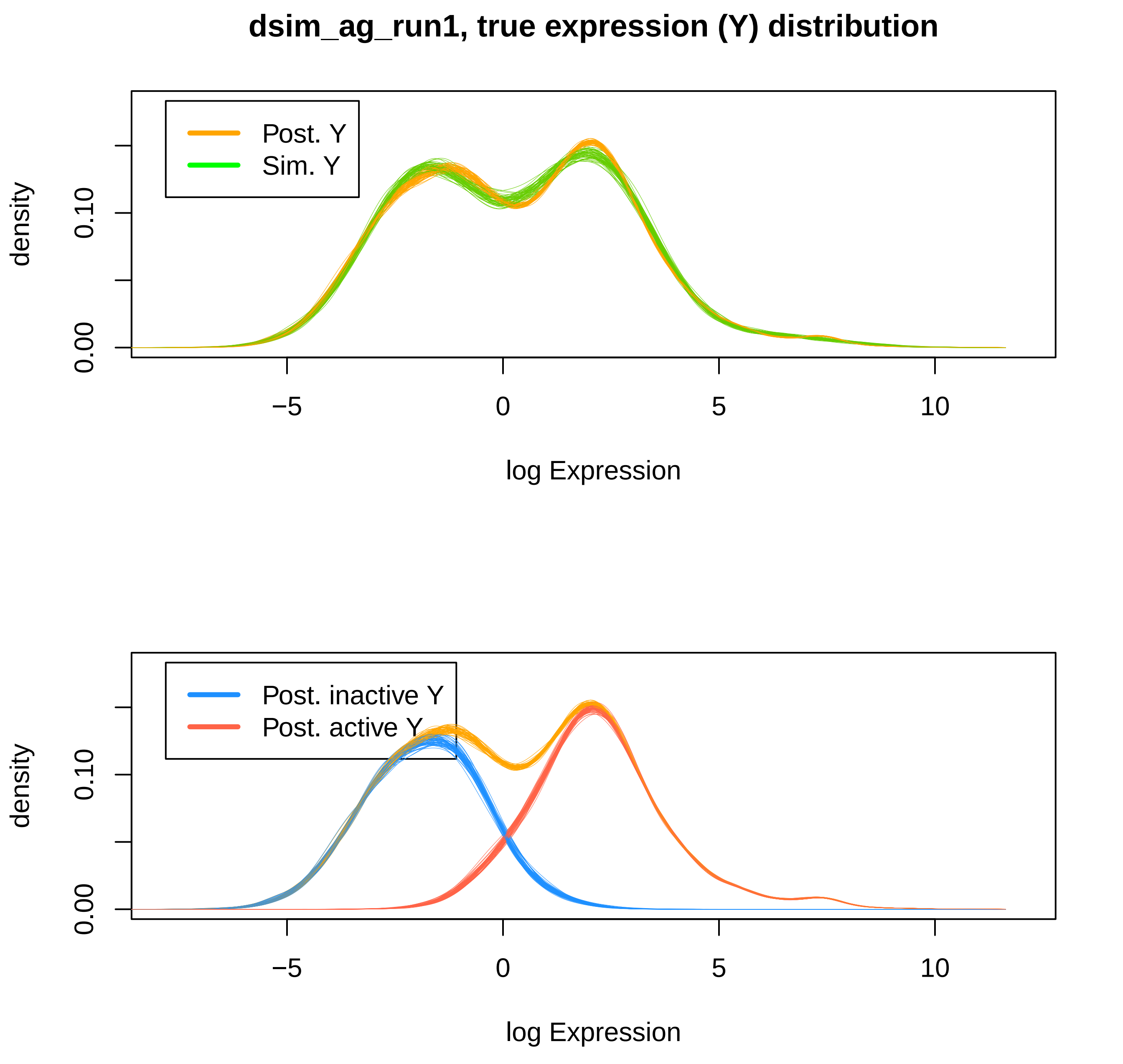

dsim_ag_run1, true expression (Y) distribution
Post. Y
Sim. Y
density
0.00
0.10
−5
0
5
10
log Expression
Post. inactive Y
Post. active Y
density
0.00
0.10
−5
0
5
10
log Expression

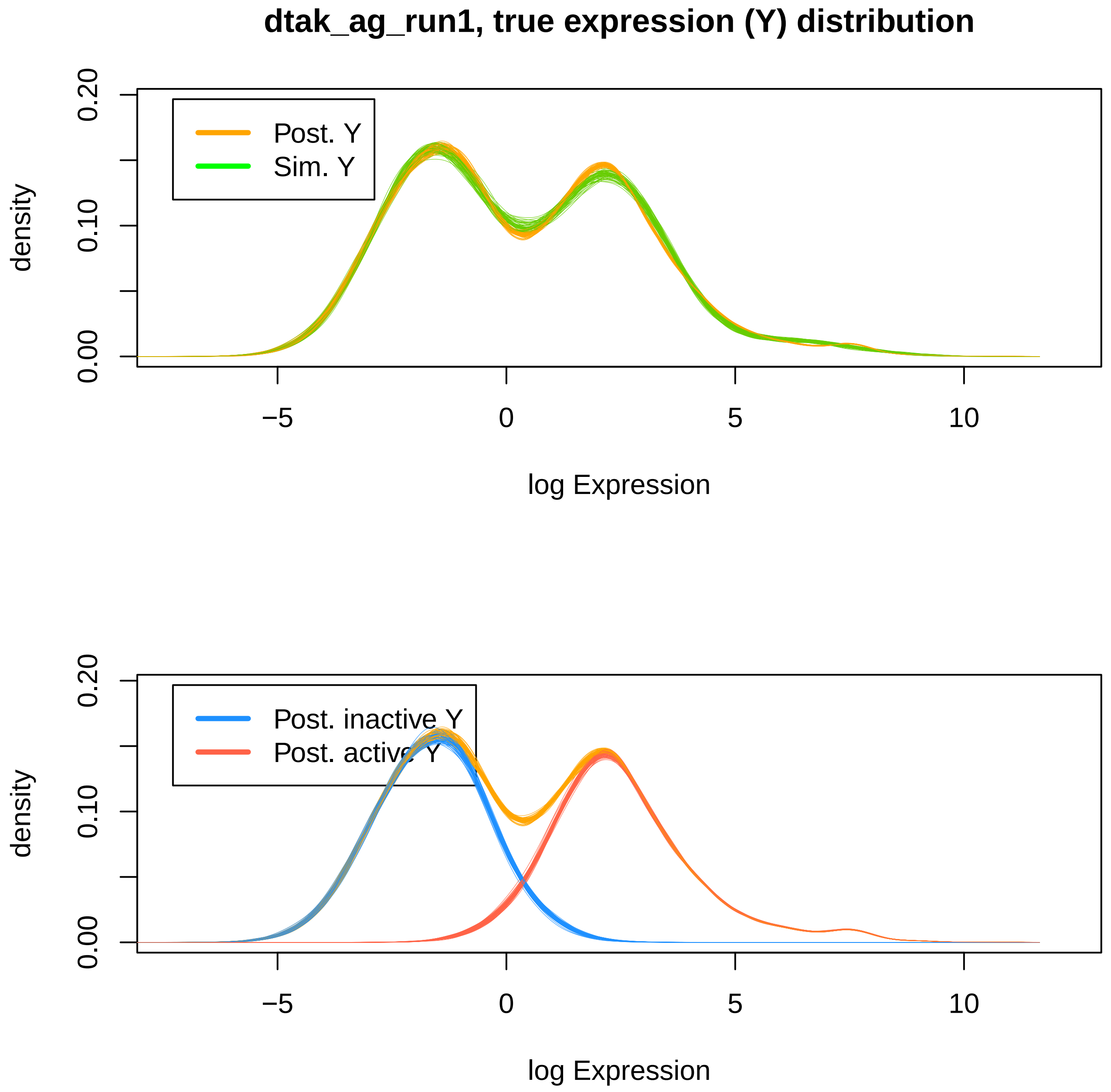

dtak_ag_run1, true expression (Y) distribution
Post. Y
Sim. Y
density
0.00
0.10
0.20
−5
0
5
10
log Expression
Post. inactive Y
Post. active Y
density
0.00
0.10
0.20
−5
0
5
10
log Expression

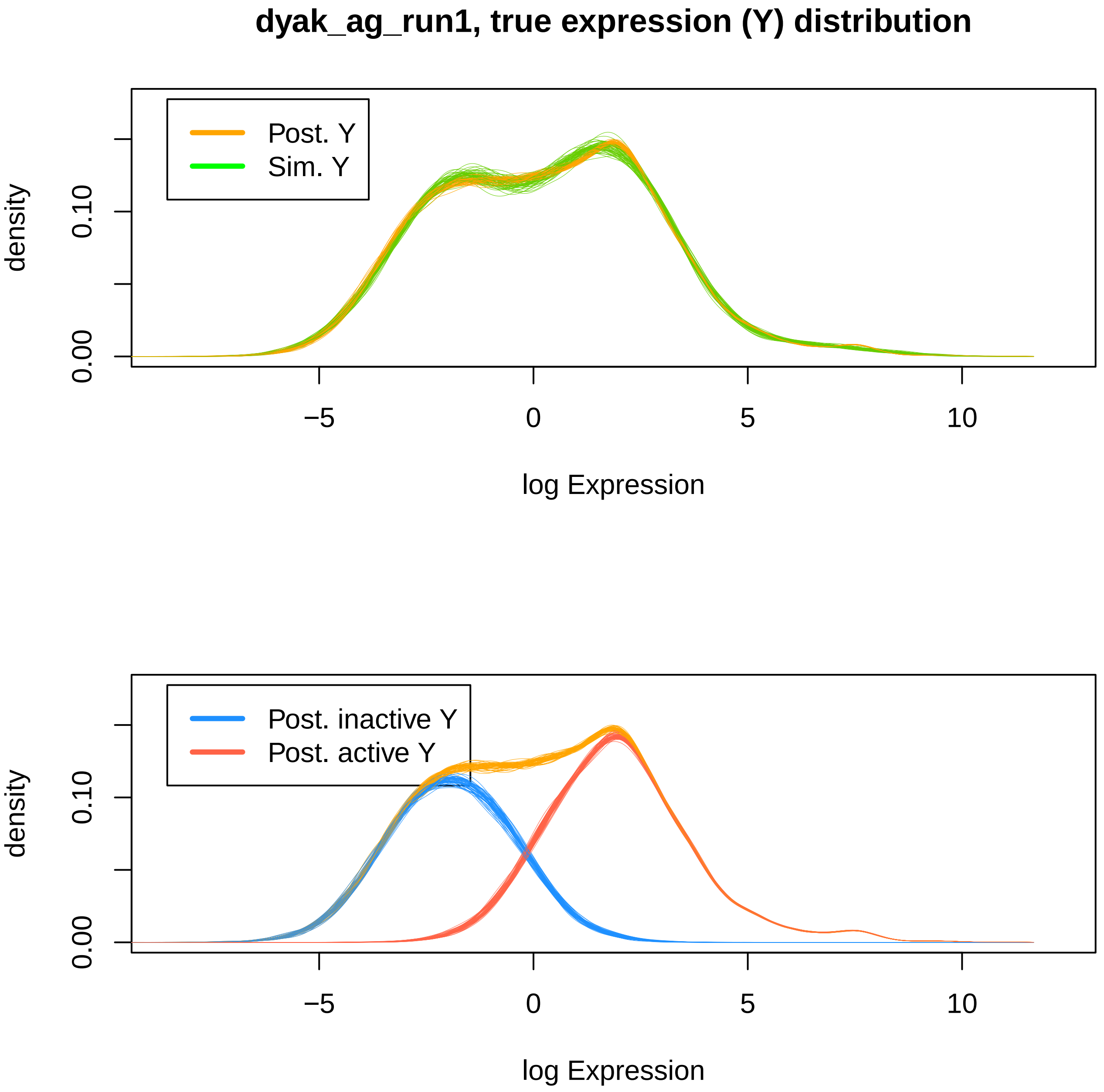
dyak_ag_run1, true expression (Y) distribution
Post. Y
Sim. Y
density
0.00
0.10
−5
0
5
10
log Expression
Post. inactive Y
Post. active Y
density
0.00
0.10
−5
0
5
10
log Expression

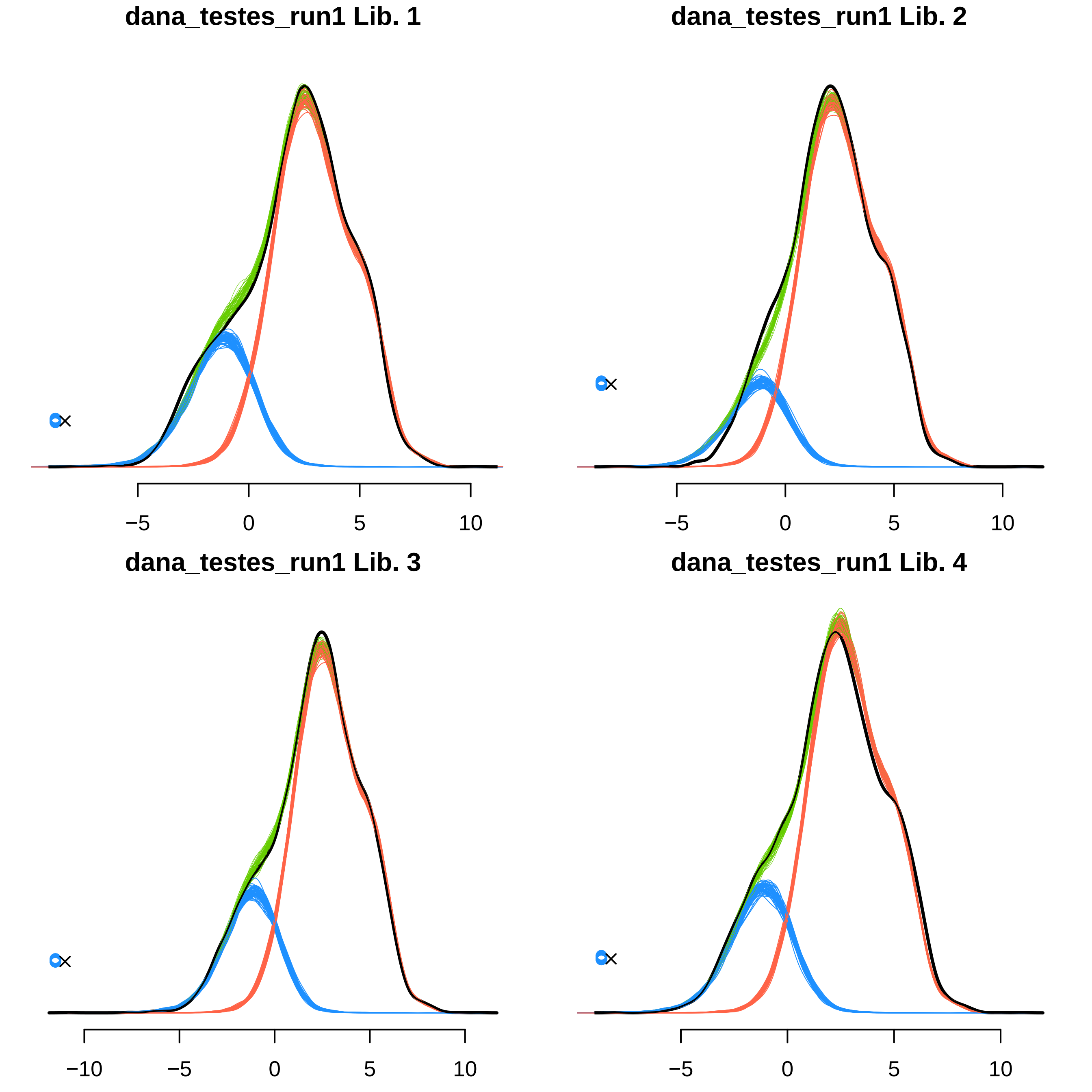
dana_testes_run1 Lib. 1
−5
0
5
10
dana_testes_run1 Lib. 2
−5
0
5
10
dana_testes_run1 Lib. 3
−10
−5
0
5
10
dana_testes_run1 Lib. 4
−5
0
5
10

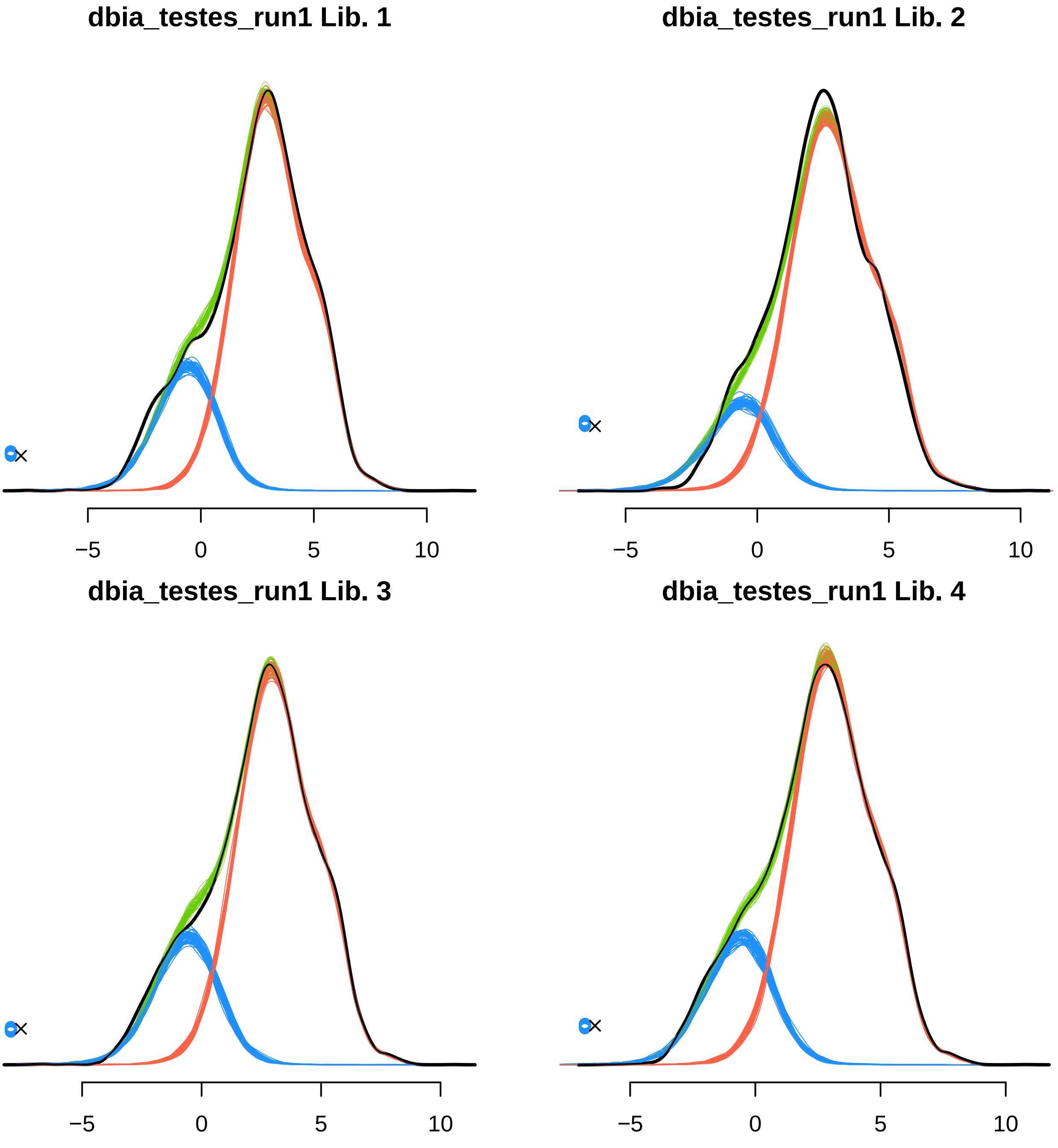

dbia_testes_run1 Lib. 1
−5
0
5
10
dbia_testes_run1 Lib. 2
−5
0
5
10
dbia_testes_run1 Lib. 3
−5
0
5
10
dbia_testes_run1 Lib. 4
−5
0
5
10

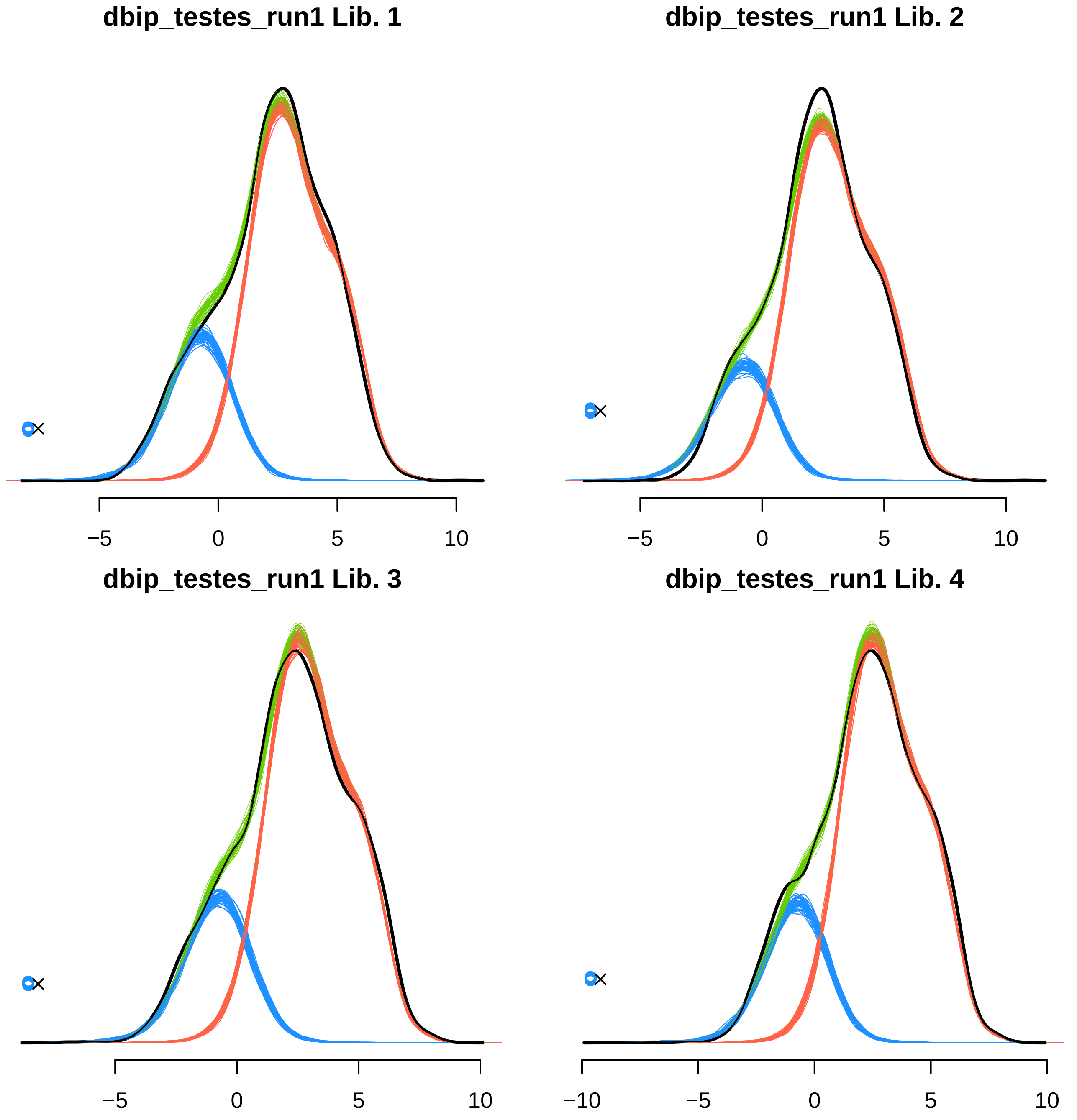

dbip_testes_run1 Lib. 1
−5
0
5
10
dbip_testes_run1 Lib. 2
−5
0
5
10
dbip_testes_run1 Lib. 3
−5
0
5
10
dbip_testes_run1 Lib. 4
−10
−5
0
5
10

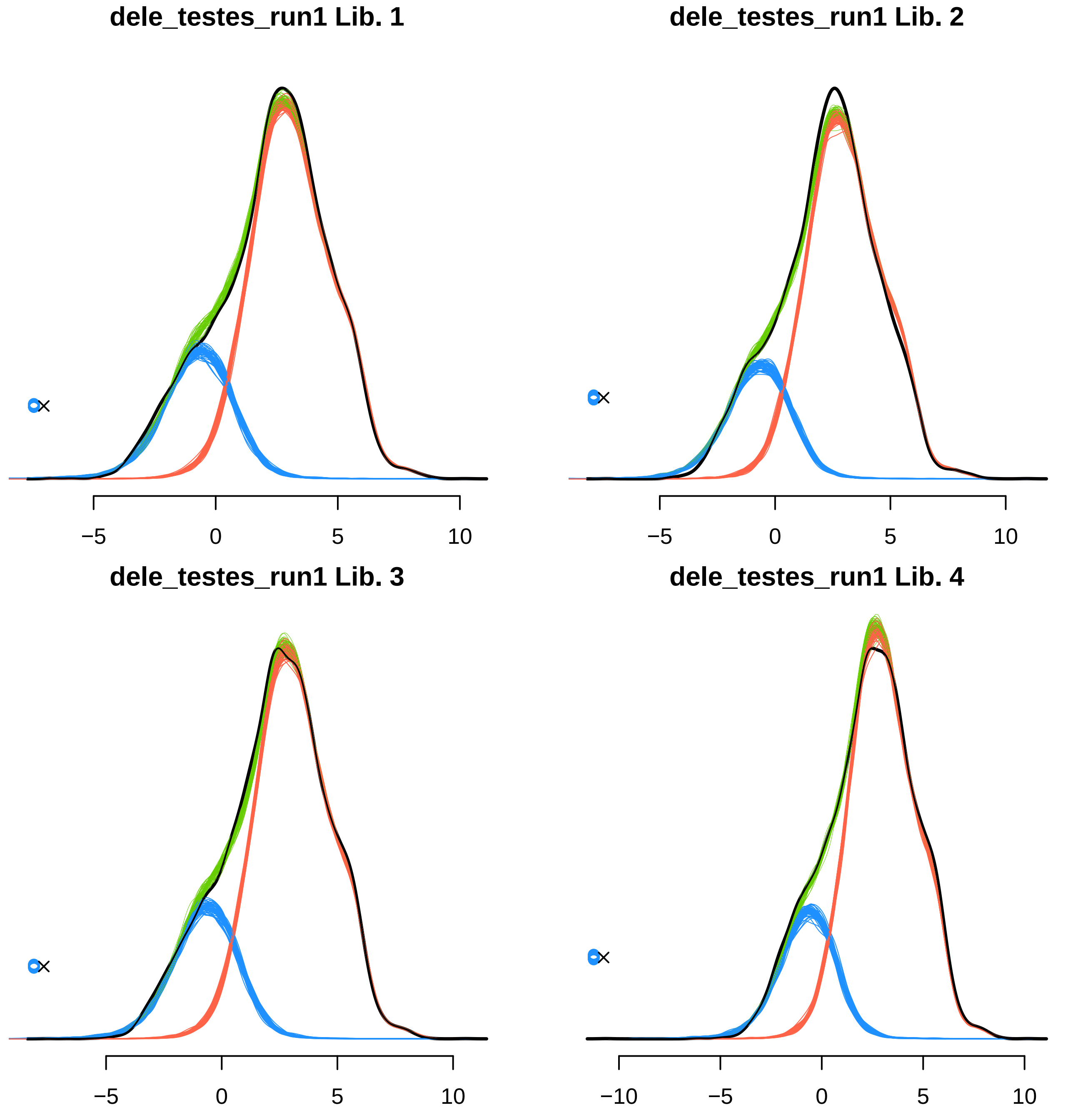
dele_testes_run1 Lib. 1
−5
0
5
10
dele_testes_run1 Lib. 2
−5
0
5
10
dele_testes_run1 Lib. 3
−5
0
5
10
dele_testes_run1 Lib. 4
−10
−5
0
5
10

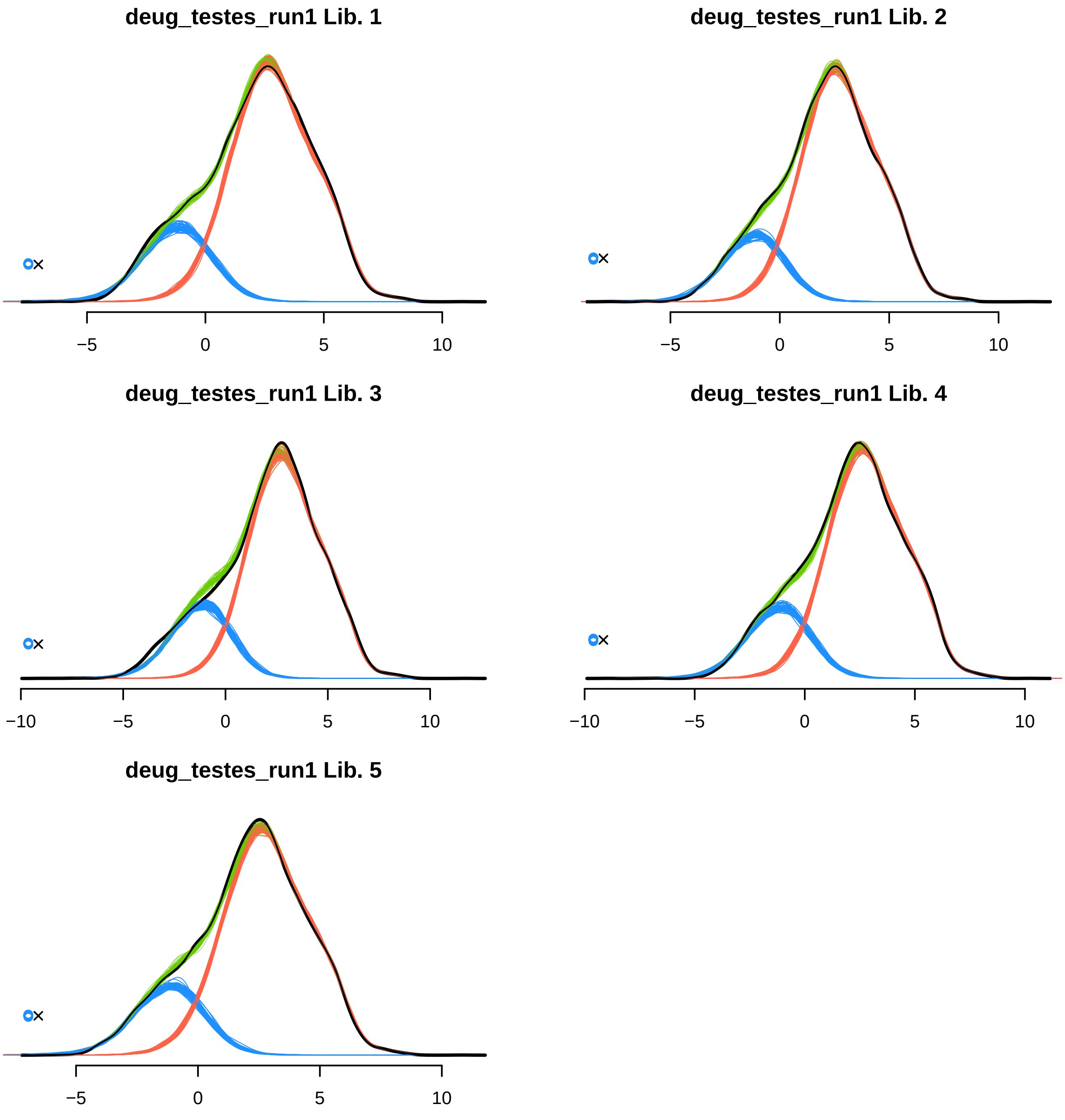
deug_testes_run1 Lib. 1
−5
0
5
10
deug_testes_run1 Lib. 2
−5
0
5
10
deug_testes_run1 Lib. 3
−10
−5
0
5
10
deug_testes_run1 Lib. 4
−10
−5
0
5
10
deug_testes_run1 Lib. 5
−5
0
5
10

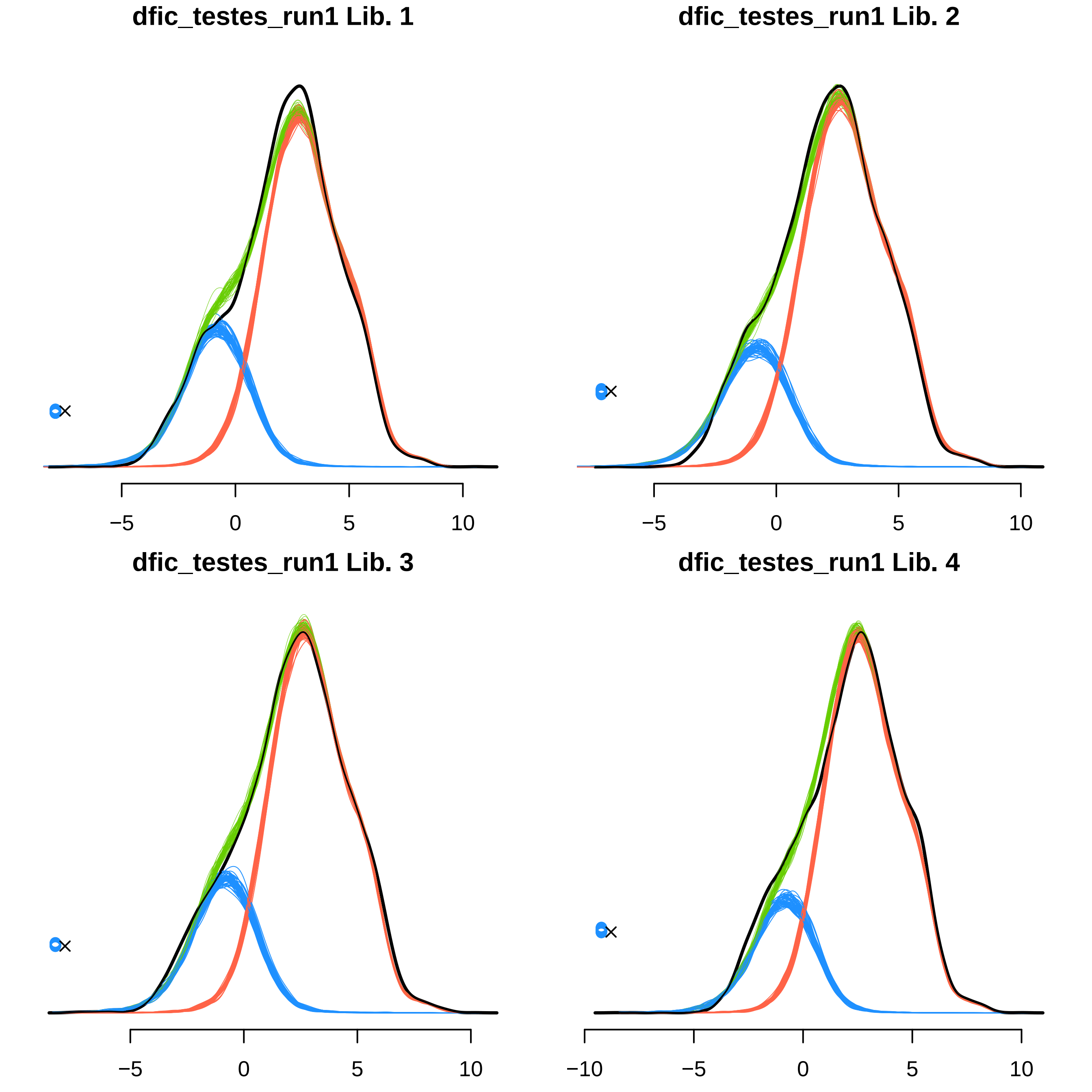
dfic_testes_run1 Lib. 1
−5
0
5
10
dfic_testes_run1 Lib. 2
−5
0
5
10
dfic_testes_run1 Lib. 3
−5
0
5
10
dfic_testes_run1 Lib. 4
−10
−5
0
5
10

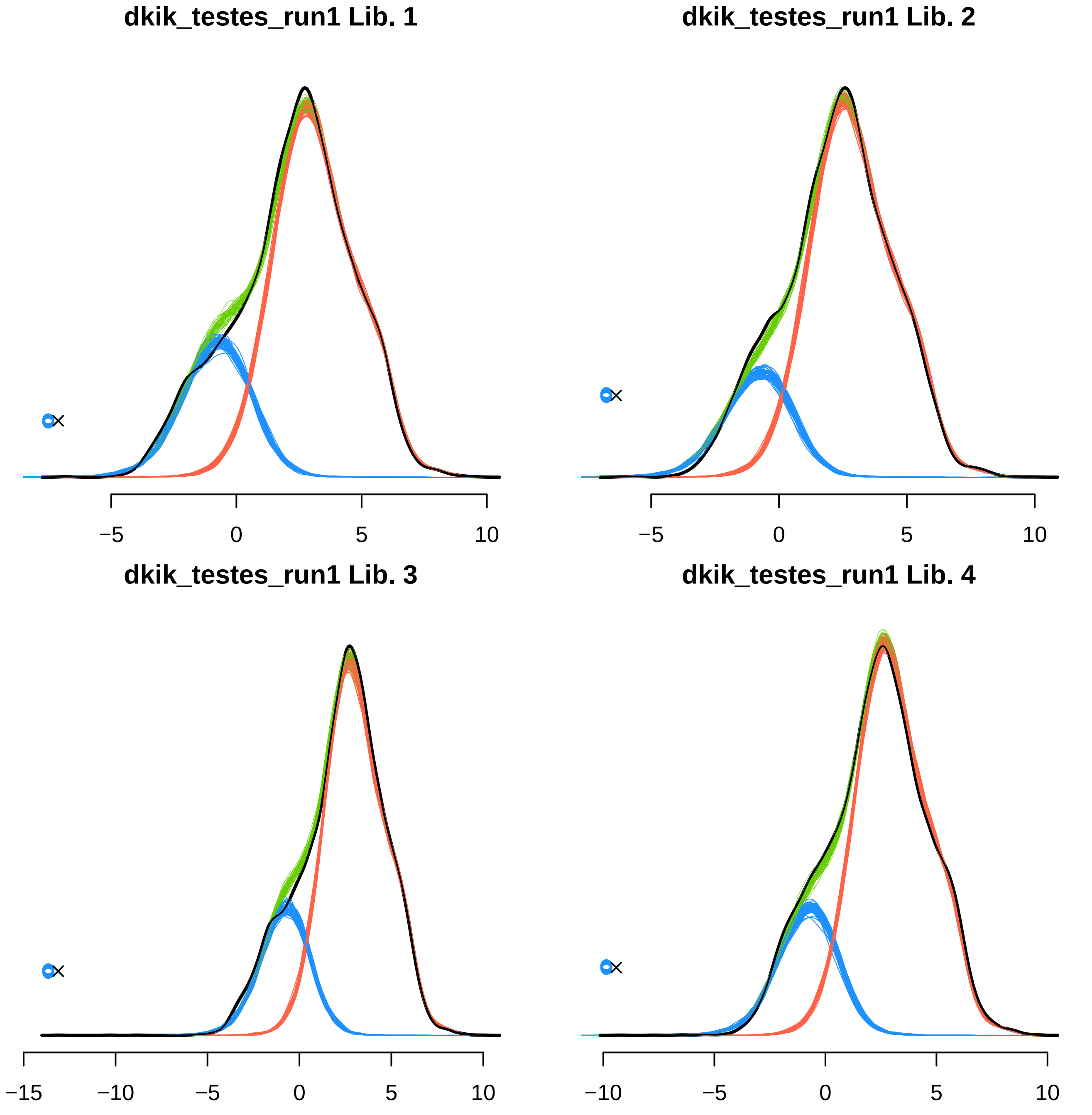
dkik_testes_run1 Lib. 1
−5
0
5
10
dkik_testes_run1 Lib. 2
−5
0
5
10
dkik_testes_run1 Lib. 3
−15
−10
−5
0
5
10
dkik_testes_run1 Lib. 4
−10
−5
0
5
10

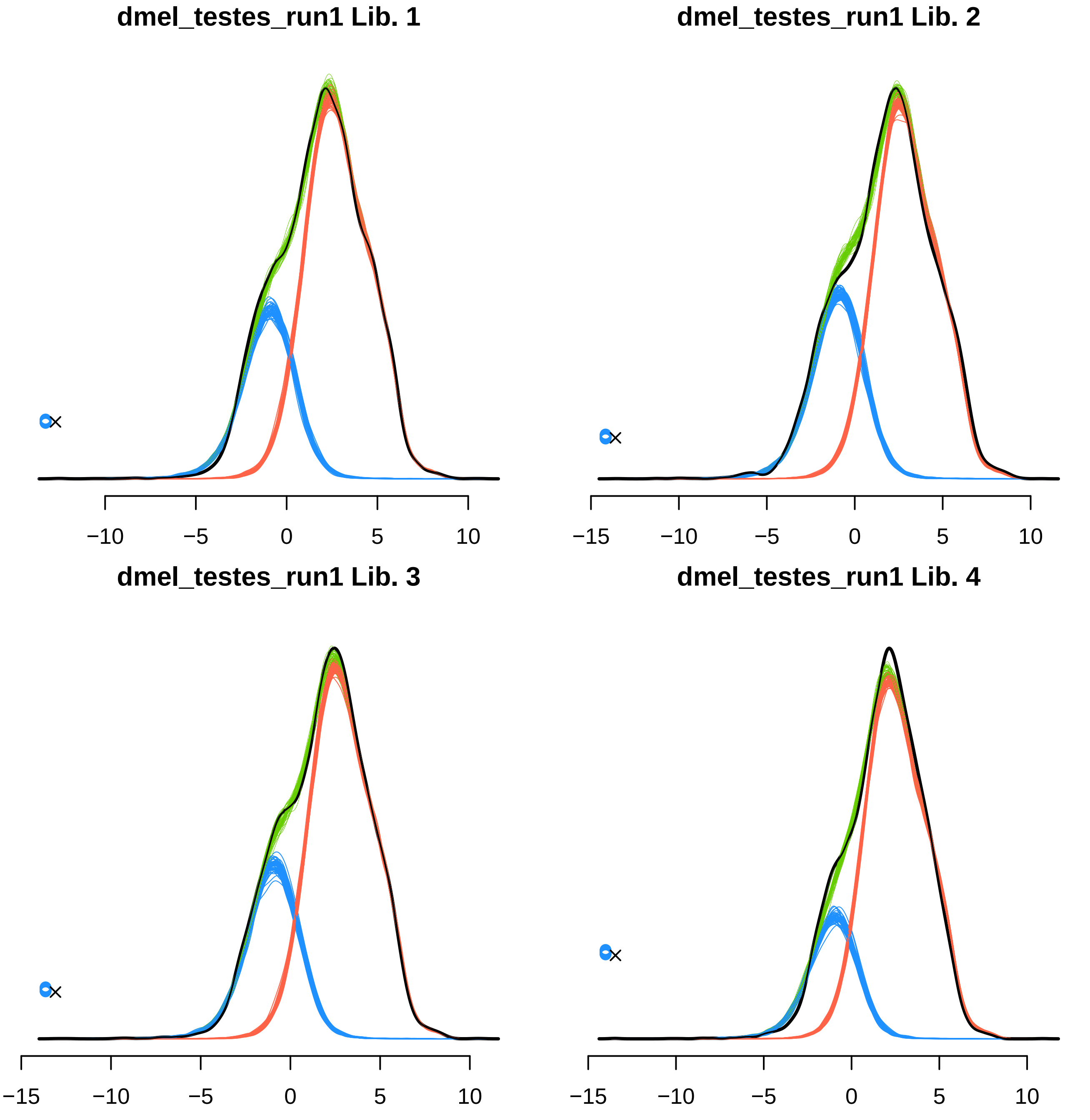

dmel_testes_run1 Lib. 1
−10
−5
0
5
10
dmel_testes_run1 Lib. 2
−15
−10
−5
0
5
10
dmel_testes_run1 Lib. 3
−15
−10
−5
0
5
10
dmel_testes_run1 Lib. 4
−15
−10
−5
0
5
10

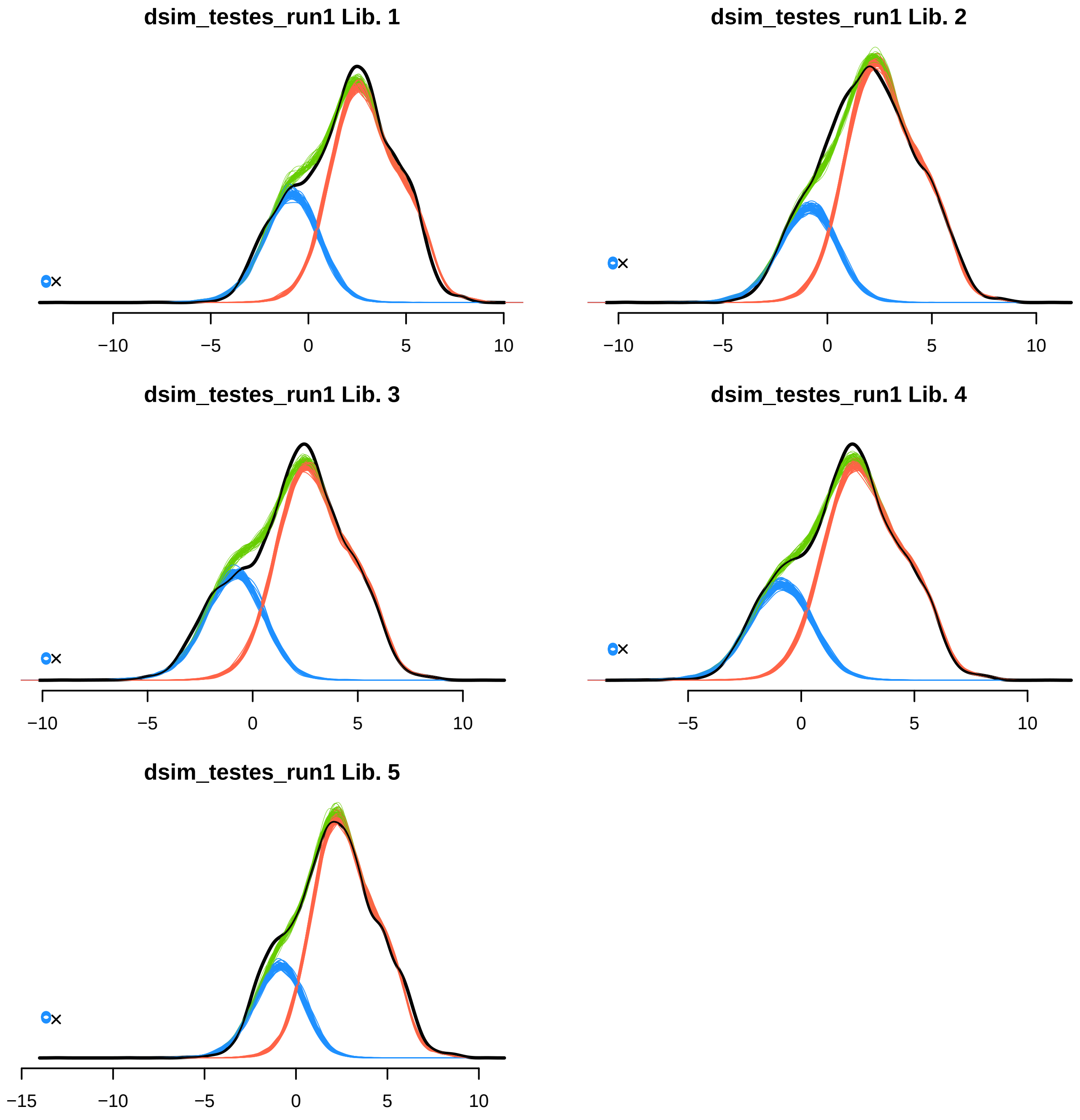
dsim_testes_run1 Lib. 1
−10
−5
0
5
10
dsim_testes_run1 Lib. 2
−10
−5
0
5
10
dsim_testes_run1 Lib. 3
−10
−5
0
5
10
dsim_testes_run1 Lib. 4
−5
0
5
10
dsim_testes_run1 Lib. 5
−15
−10
−5
0
5
10

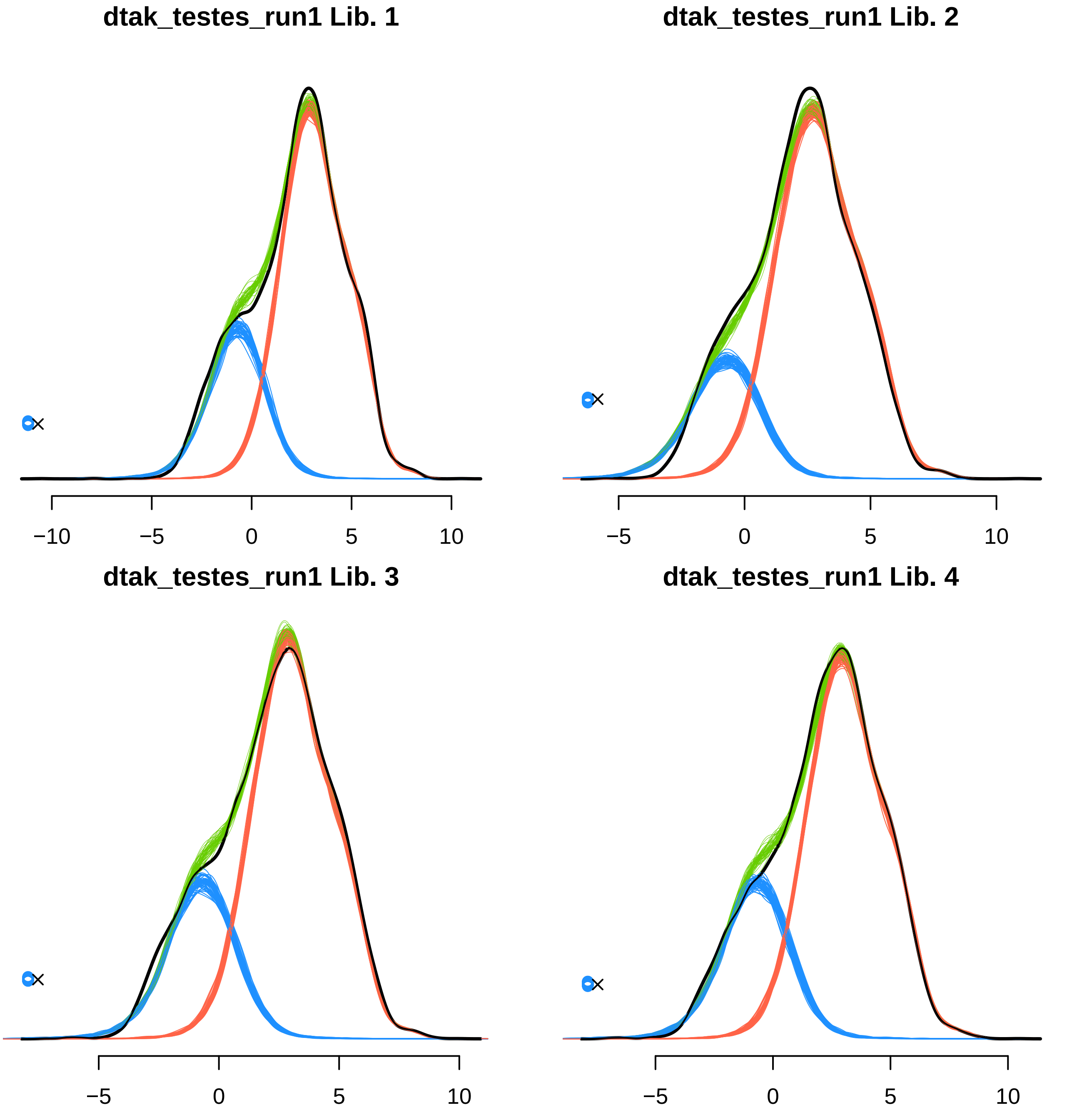
dtak_testes_run1 Lib. 1
−10
−5
0
5
10
dtak_testes_run1 Lib. 2
−5
0
5
10
dtak_testes_run1 Lib. 3
−5
0
5
10
dtak_testes_run1 Lib. 4
−5
0
5
10

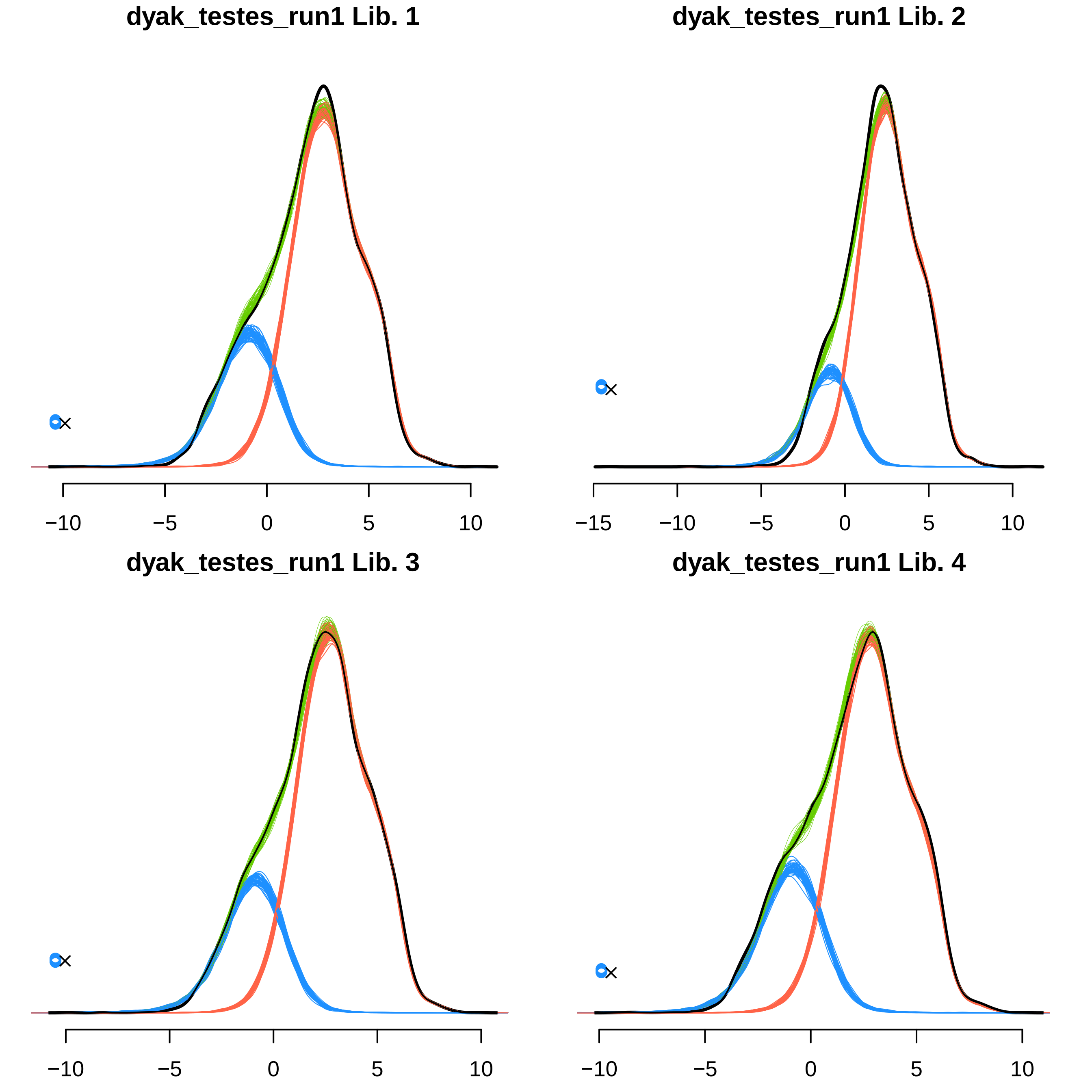
dyak_testes_run1 Lib. 1
−10
−5
0
5
10
dyak_testes_run1 Lib. 2
−15
−10
−5
0
5
10
dyak_testes_run1 Lib. 3
−10
−5
0
5
10
dyak_testes_run1 Lib. 4
−10
−5
0
5
10

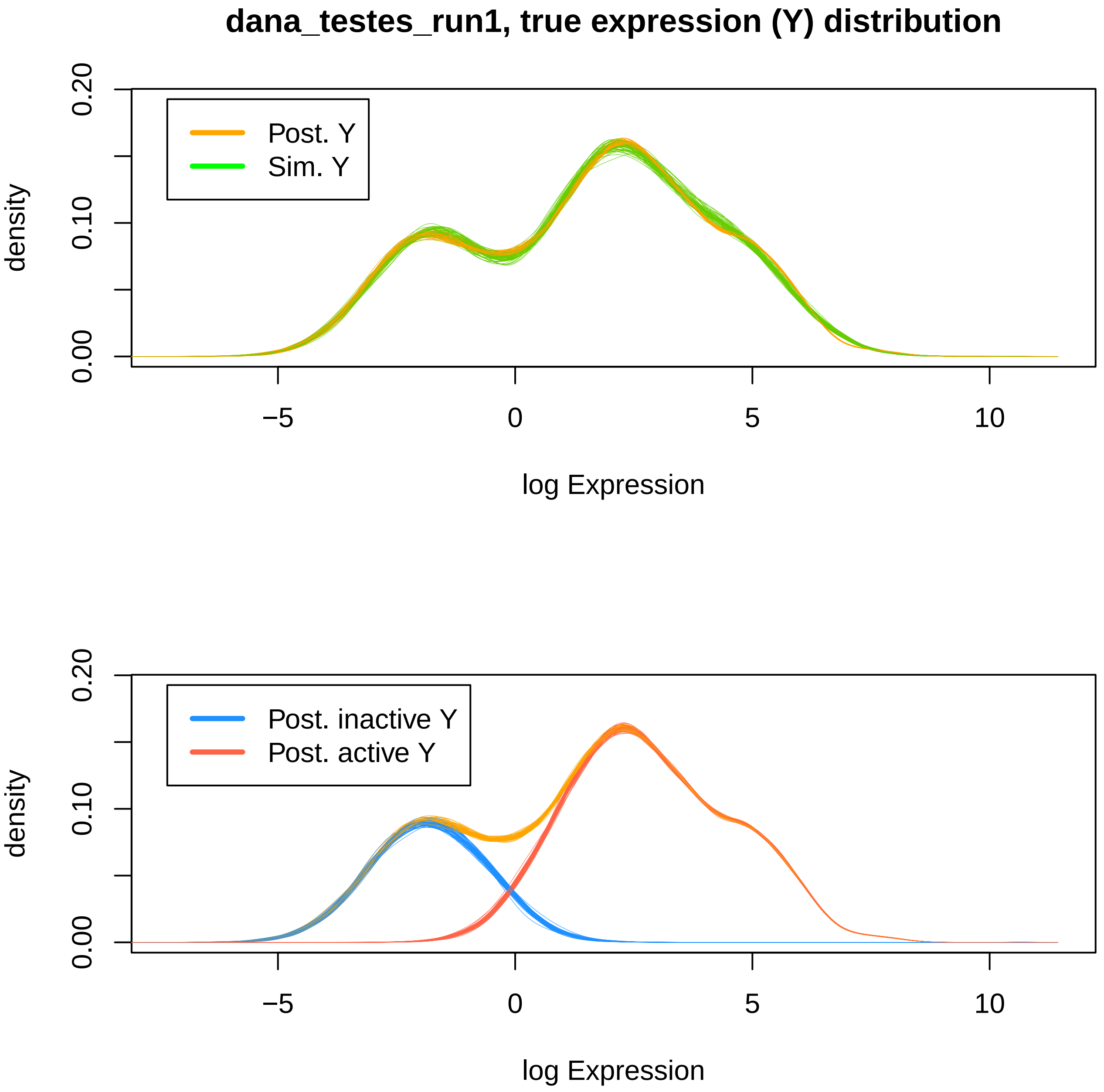
dana_testes_run1, true expression (Y) distribution
Post. Y
Sim. Y
density
0.00
0.10
0.20
−5
0
5
10
log Expression
Post. inactive Y
Post. active Y
density
0.00
0.10
0.20
−5
0
5
10
log Expression

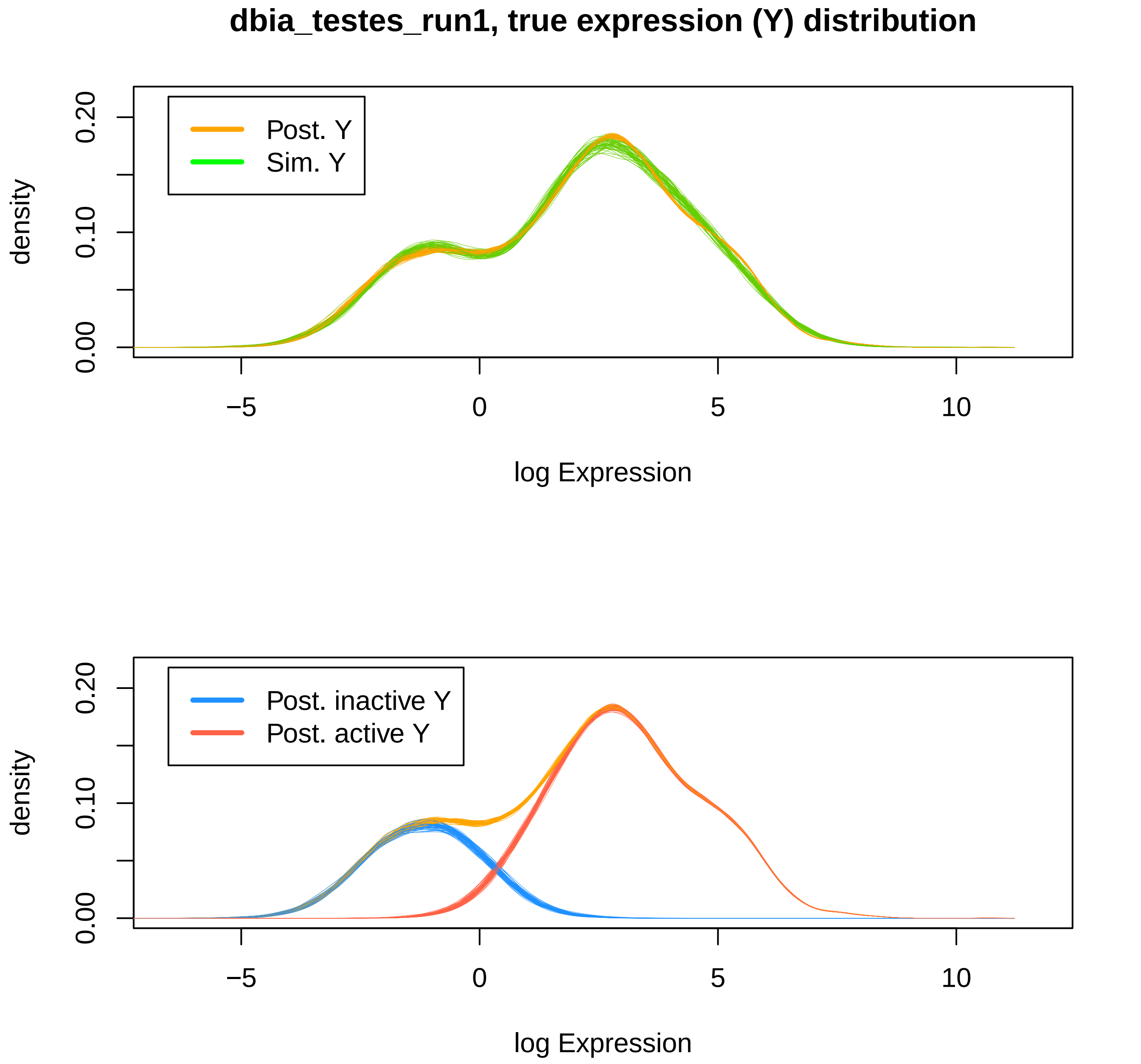

dbia_testes_run1, true expression (Y) distribution
Post. Y
Sim. Y
density
0.00
0.10
0.20
−5
0
5
10
log Expression
Post. inactive Y
Post. active Y
density
0.00
0.10
0.20
−5
0
5
10
log Expression

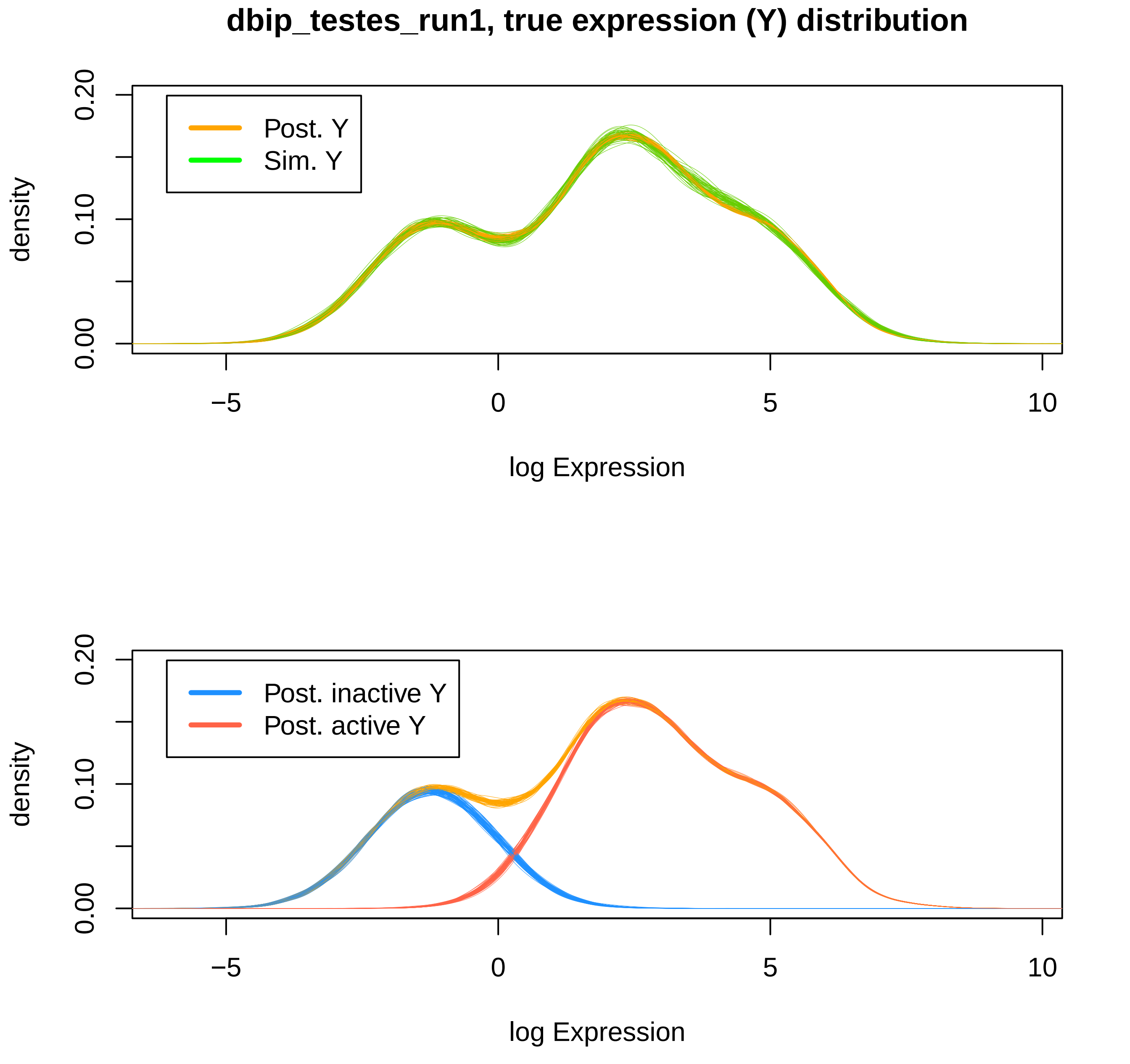

dbip_testes_run1, true expression (Y) distribution
Post. Y
Sim. Y
density
0.00
0.10
0.20
−5
0
5
10
log Expression
Post. inactive Y
Post. active Y
density
0.00
0.10
0.20
−5
0
5
10
log Expression

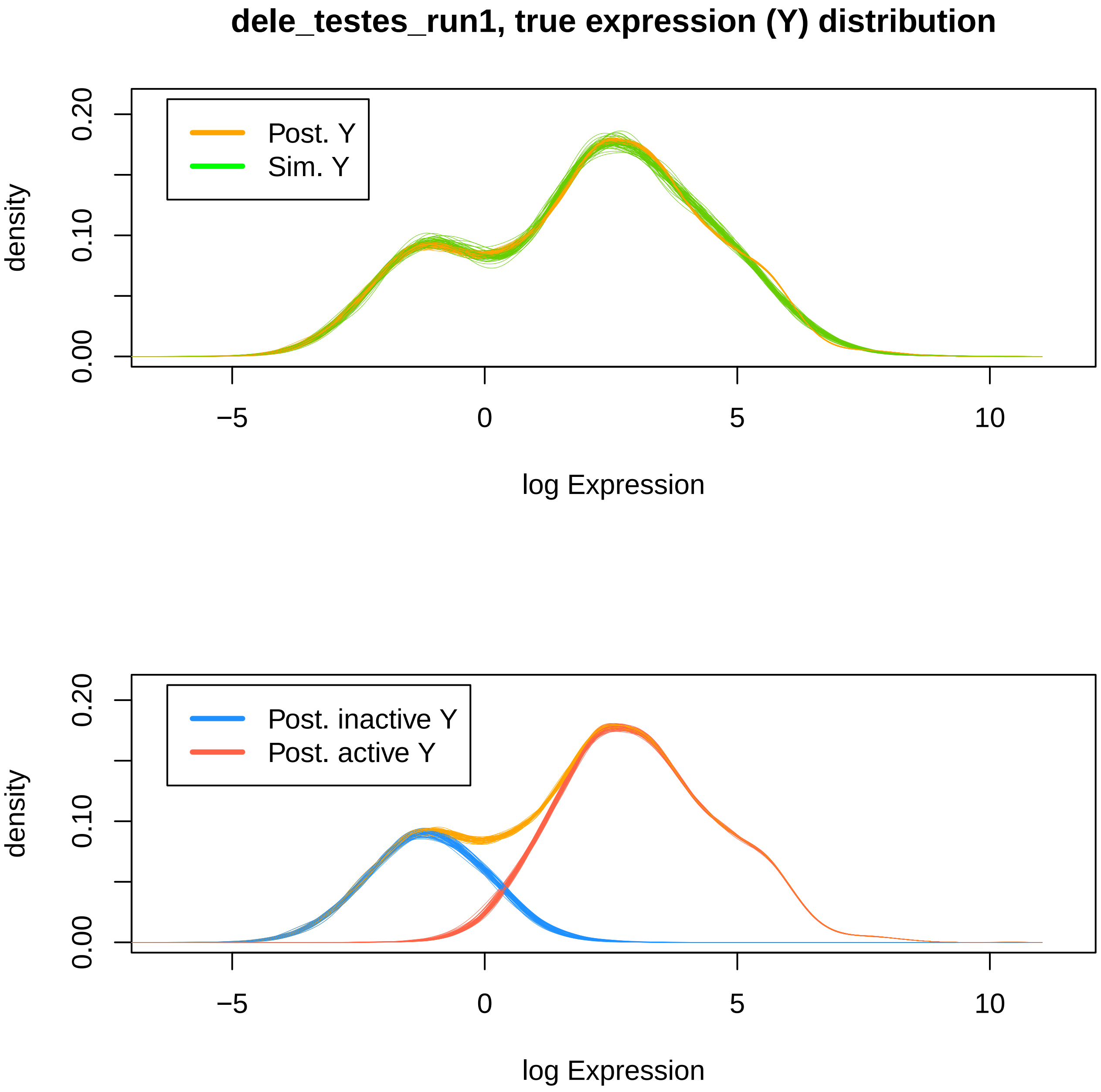
dele_testes_run1, true expression (Y) distribution
Post. Y
Sim. Y
density
0.00
0.10
0.20
−5
0
5
10
log Expression
Post. inactive Y
Post. active Y
density
0.00
0.10
0.20
−5
0
5
10
log Expression

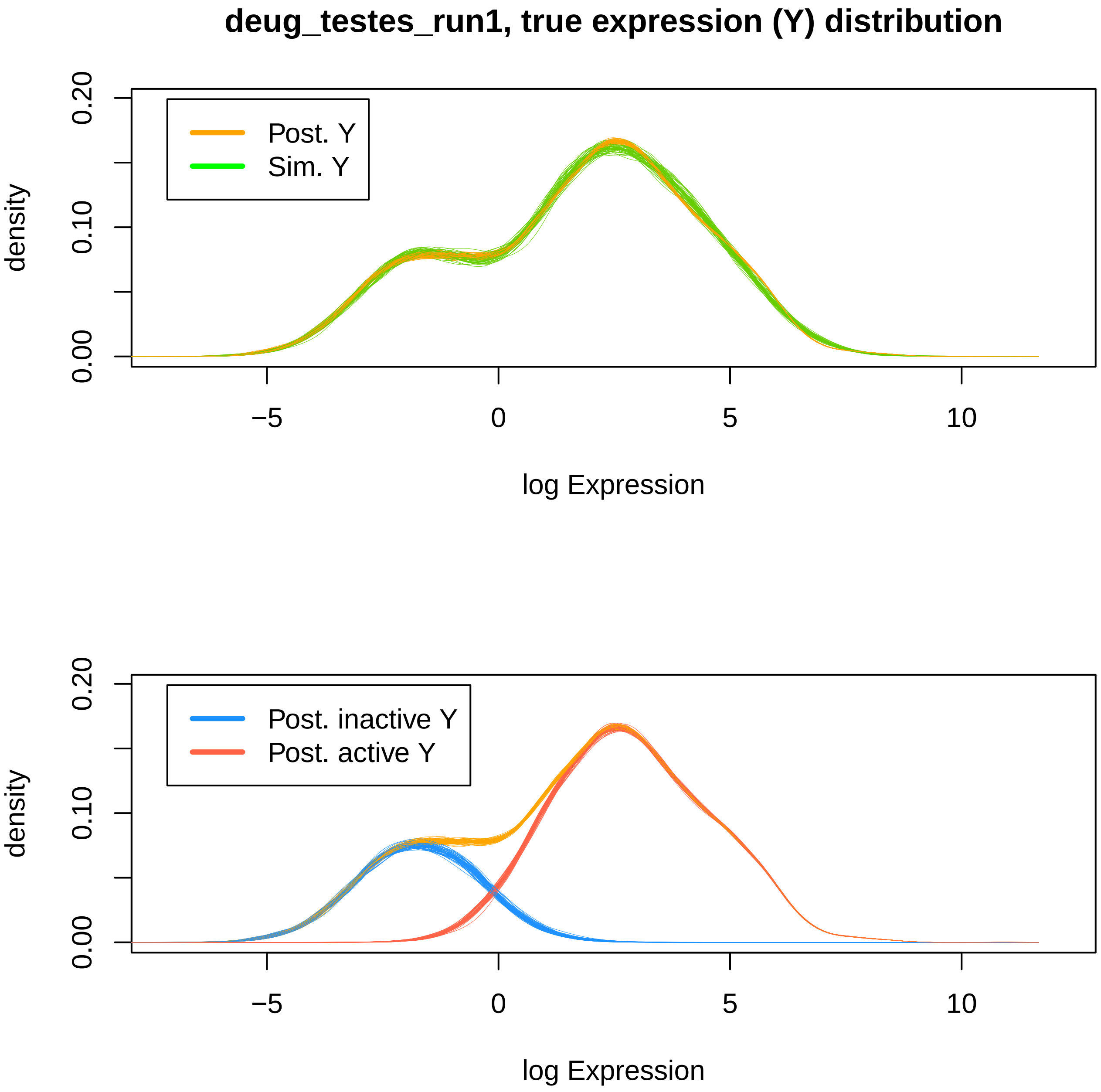

deug_testes_run1, true expression (Y) distribution
Post. Y
Sim. Y
density
0.00
0.10
0.20
−5
0
5
10
log Expression
Post. inactive Y
Post. active Y
density
0.00
0.10
0.20
−5
0
5
10
log Expression

dfic_testes_run1, true expression (Y) distribution

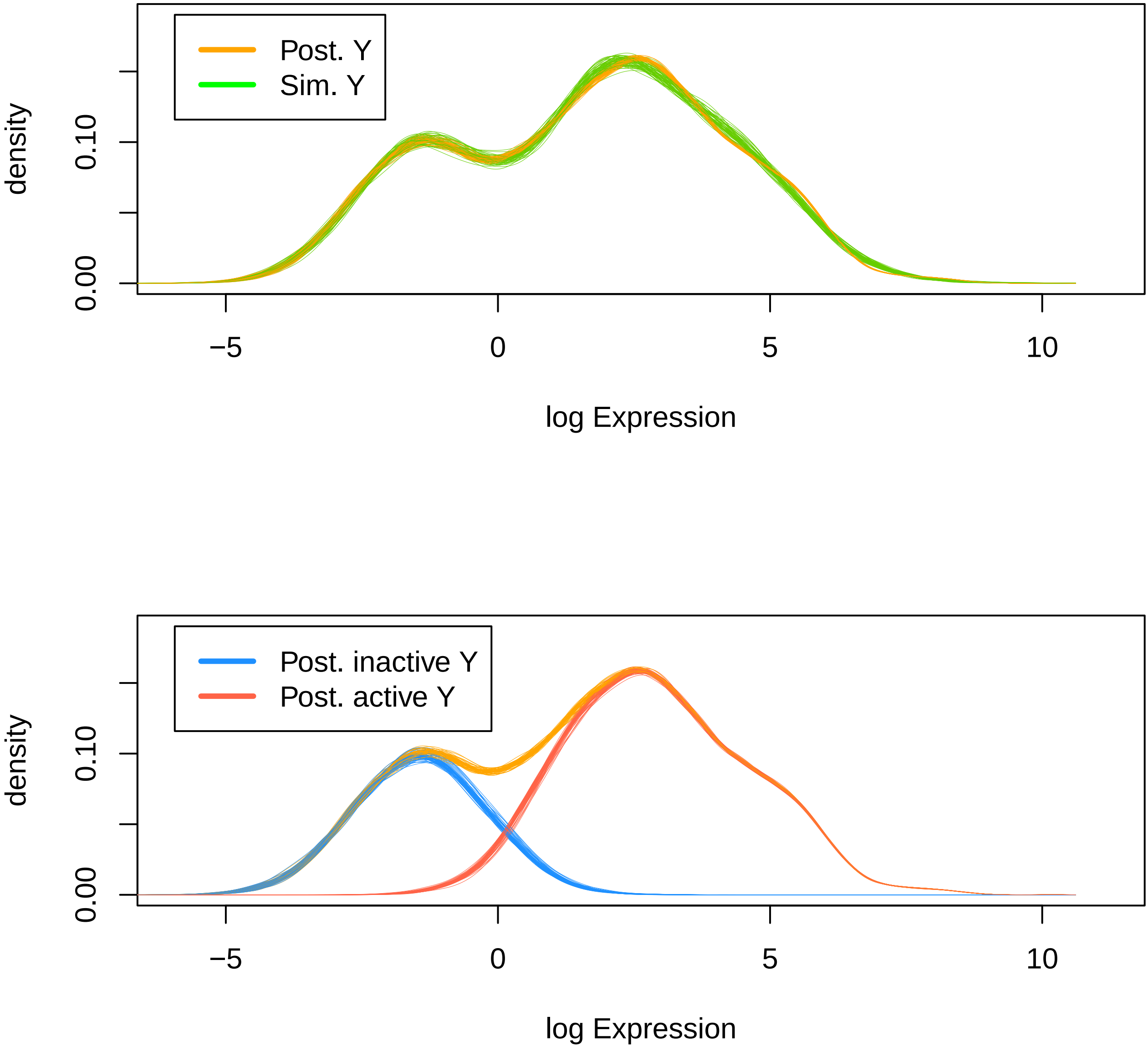

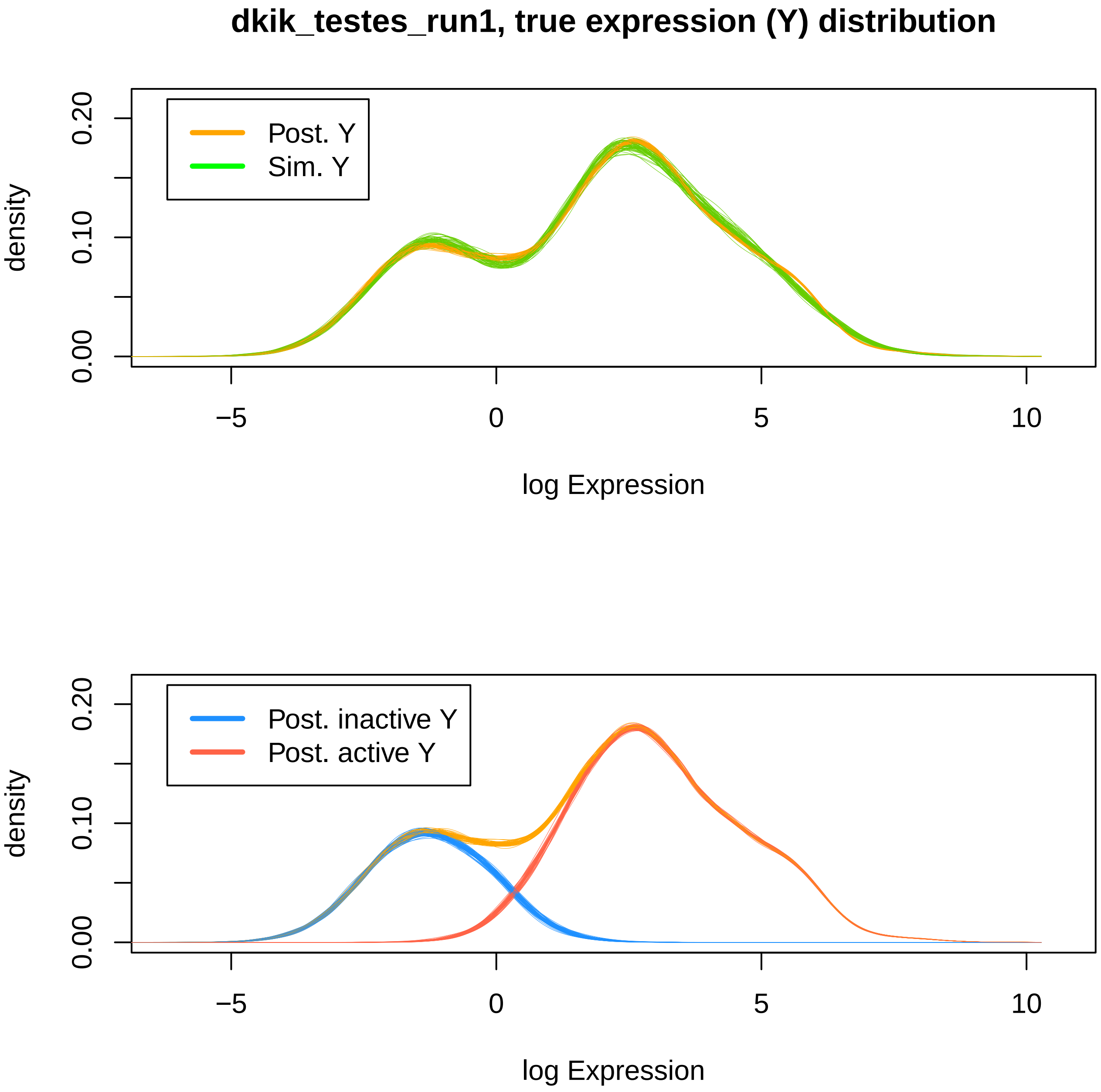
dkik_testes_run1, true expression (Y) distribution
Post. Y
Sim. Y
density
0.00
0.10
0.20
−5
0
5
10
log Expression
Post. inactive Y
Post. active Y
density
0.00
0.10
0.20
−5
0
5
10
log Expression

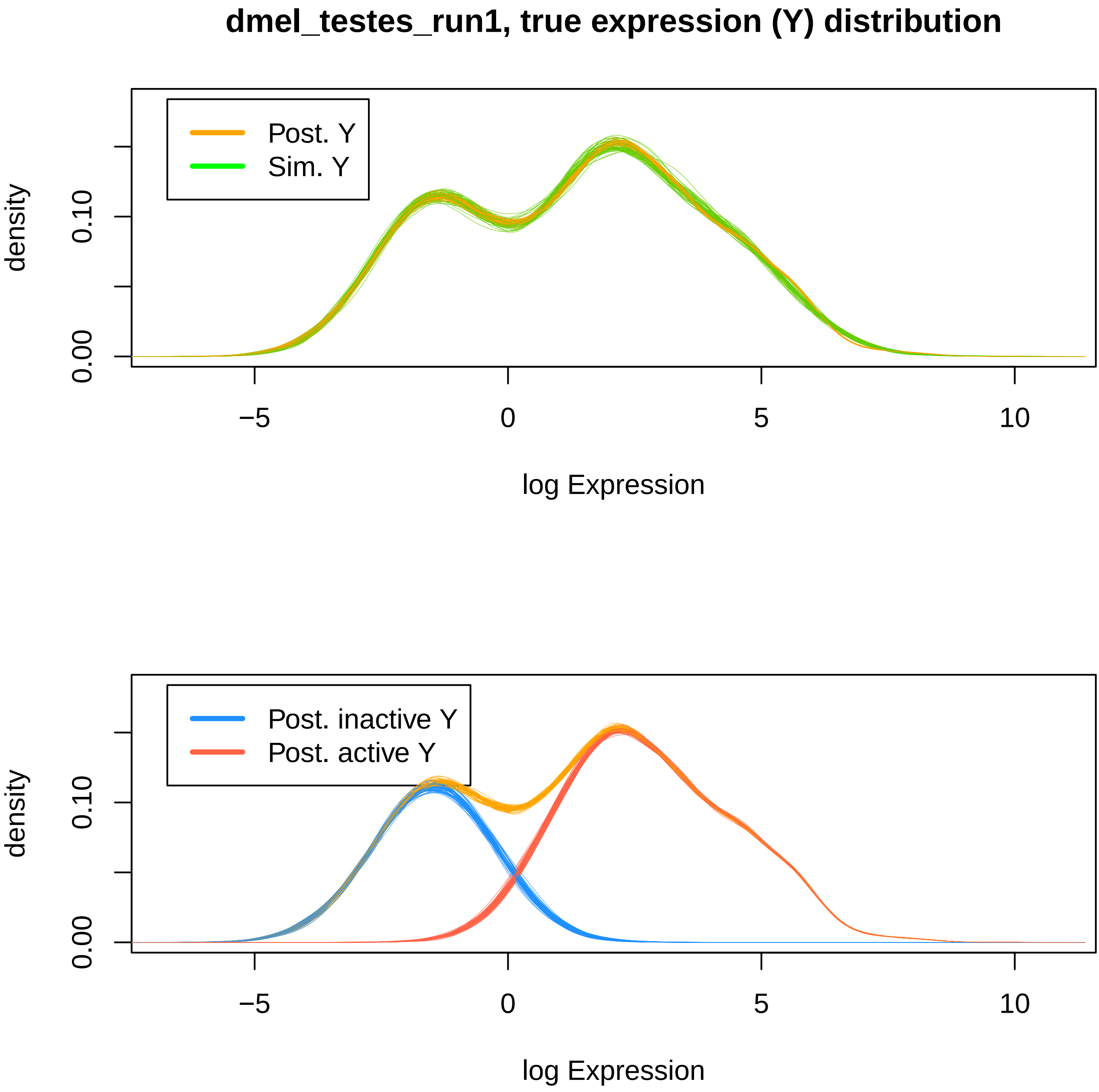

dmel_testes_run1, true expression (Y) distribution
Post. Y
Sim. Y
density
0.00
0.10
−5
0
5
10
log Expression
Post. inactive Y
Post. active Y
density
0.00
0.10
−5
0
5
10
log Expression

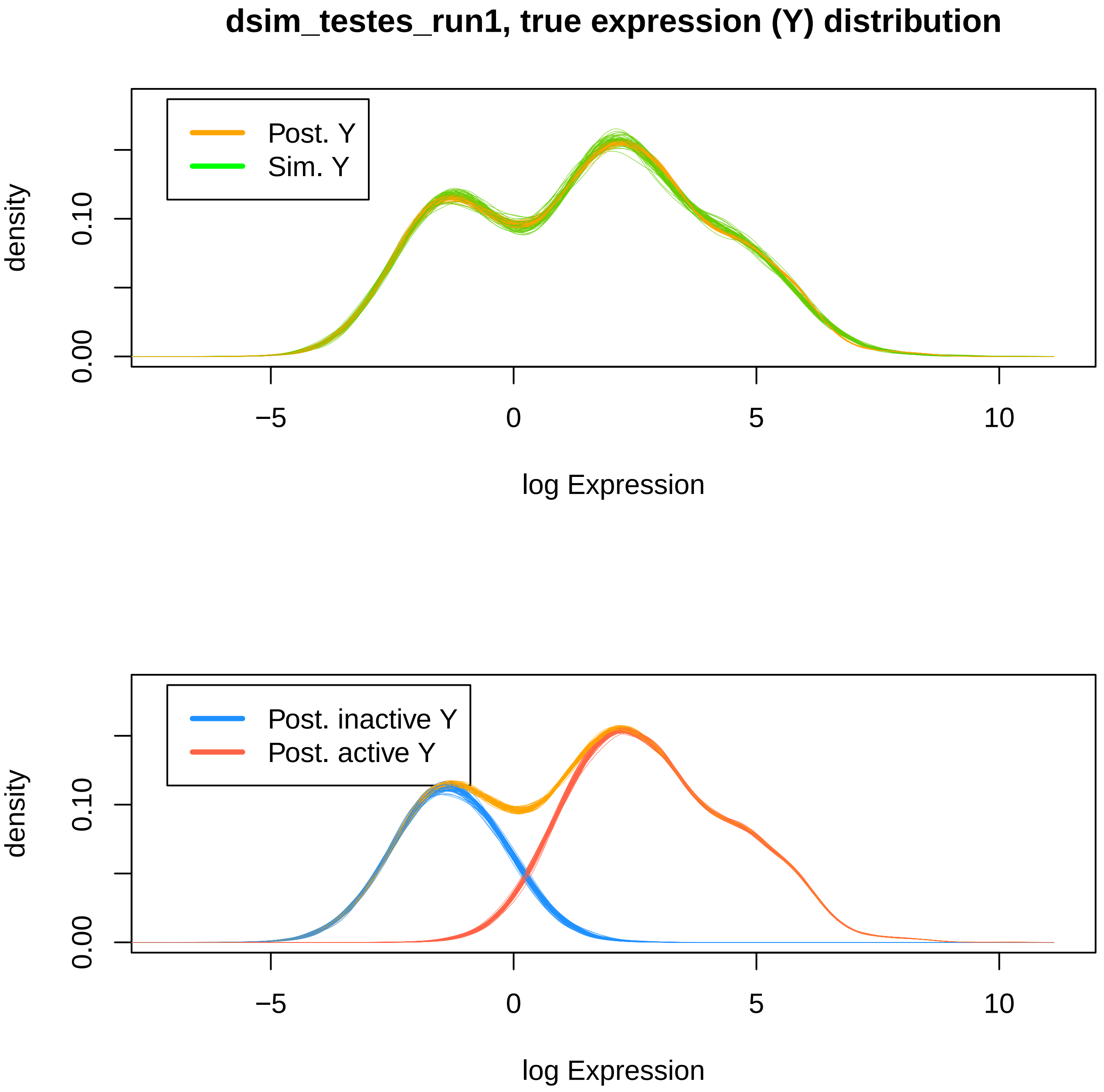

dsim_testes_run1, true expression (Y) distribution
Post. Y
Sim. Y
density
0.00
0.10
−5
0
5
10
log Expression
Post. inactive Y
Post. active Y
density
0.00
0.10
−5
0
5
10
log Expression

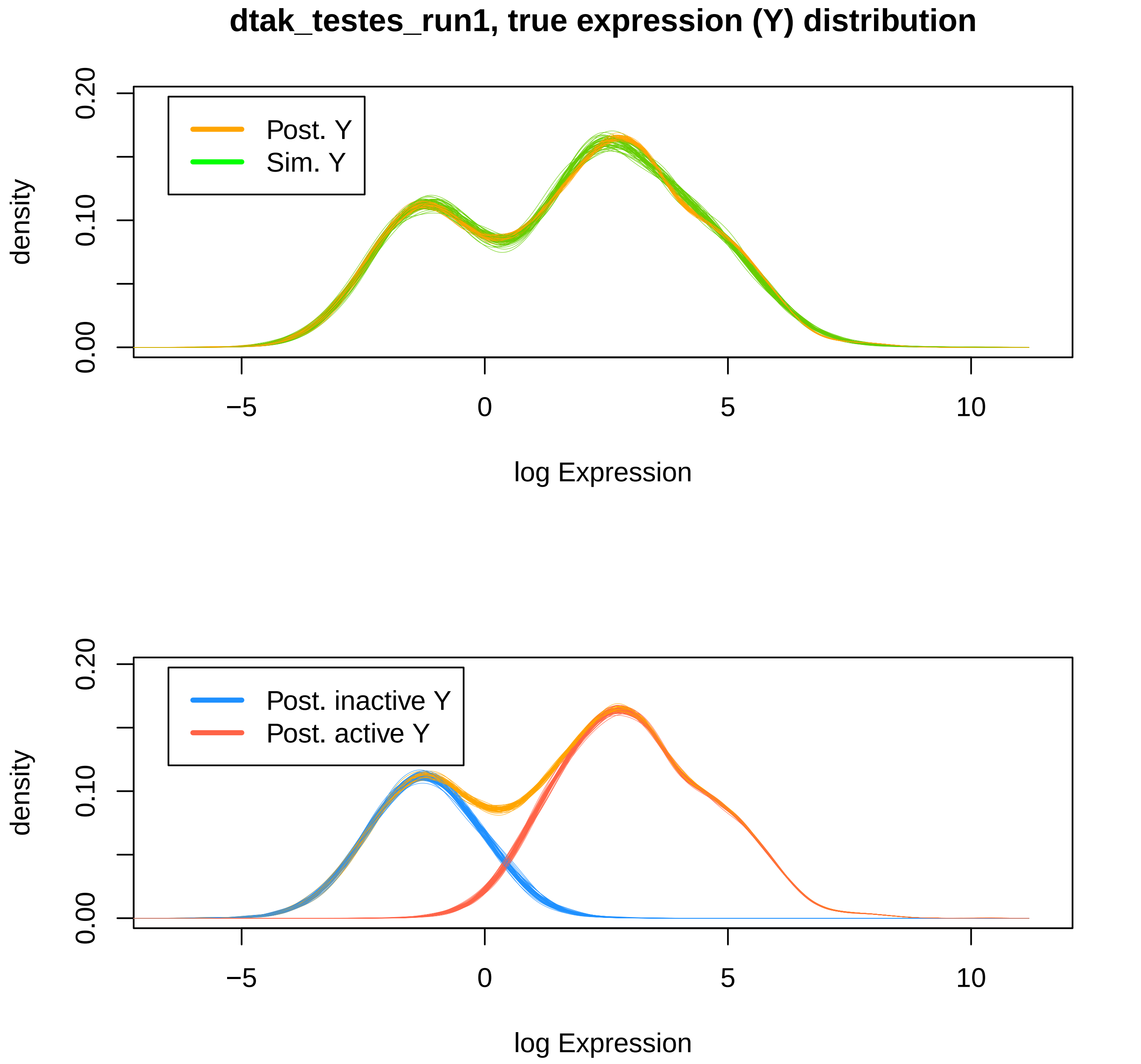
dtak_testes_run1, true expression (Y) distribution
Post. Y
Sim. Y
density
0.00
0.10
0.20
−5
0
5
10
log Expression
Post. inactive Y
Post. active Y
density
0.00
0.10
0.20
−5
0
5
10
log Expression

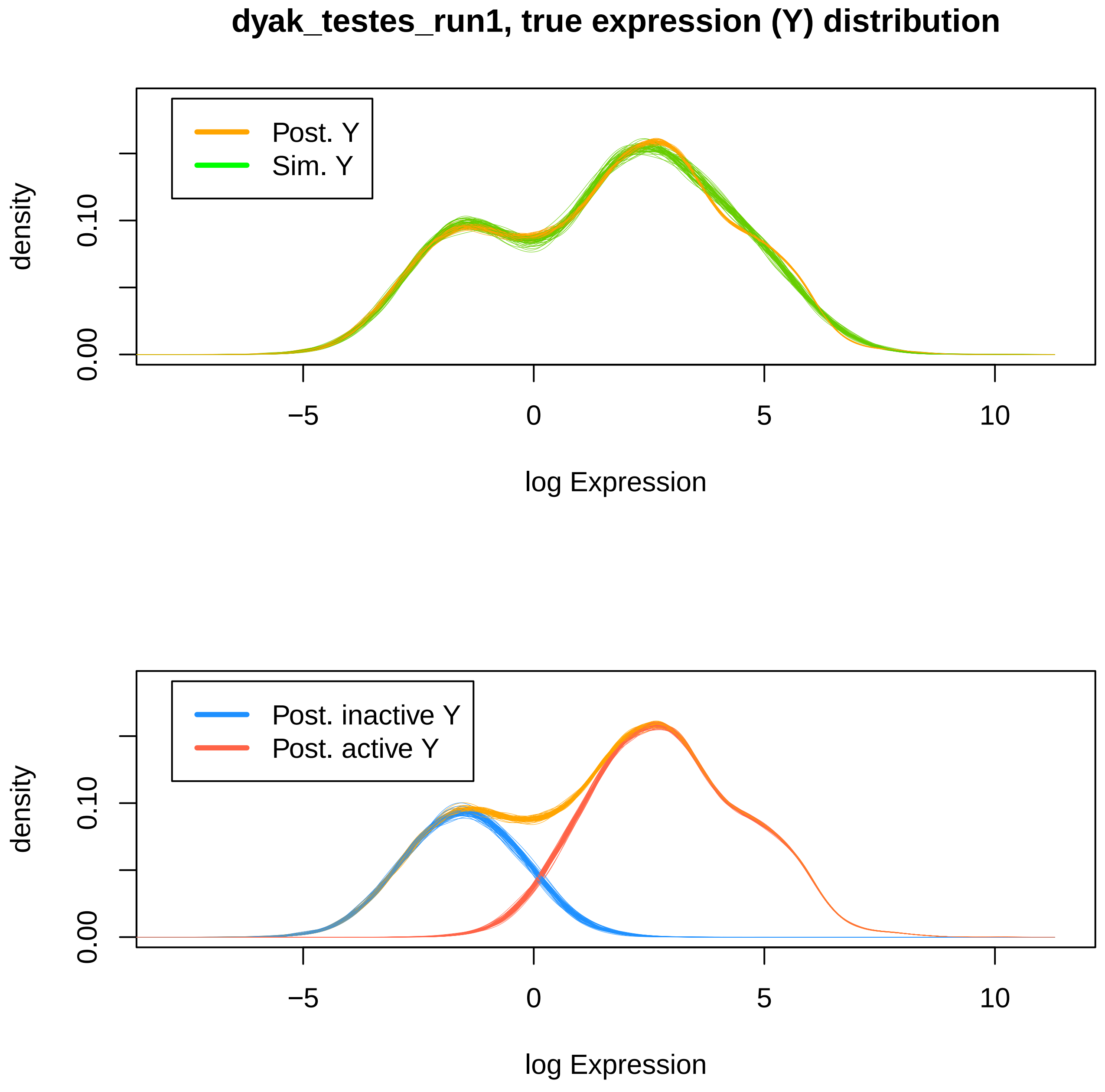
dyak_testes_run1, true expression (Y) distribution
Post. Y
Sim. Y
density
0.00
0.10
−5
0
5
10
log Expression
Post. inactive Y
Post. active Y
density
0.00
0.10
−5
0
5
10
log Expression
